# A Review of State of the Art in Phosphine Ligated Gold Clusters and Application in Catalysis

**DOI:** 10.1002/advs.202105692

**Published:** 2022-03-25

**Authors:** Rohul H. Adnan, Jenica Marie L. Madridejos, Abdulrahman S. Alotabi, Gregory F. Metha, Gunther G. Andersson

**Affiliations:** ^1^ Department of Chemistry, Faculty of Science Center for Hydrogen Energy Universiti Teknologi Malaysia (UTM) Johor Bahru 81310 Malaysia; ^2^ Department of Chemistry University of Adelaide Adelaide South Australia 5005 Australia; ^3^ Flinders Institute for NanoScale Science and Technology Flinders University Adelaide South Australia 5042 Australia; ^4^ Department of Physics Faculty of Science and Arts in Baljurashi Albaha University Baljurashi 65655 Saudi Arabia

**Keywords:** atomically precise clusters, gold–phosphine clusters, phosphine ligands, superatoms

## Abstract

Atomically precise gold clusters are highly desirable due to their well‐defined structure which allows the study of structure–property relationships. In addition, they have potential in technological applications such as nanoscale catalysis. The structural, chemical, electronic, and optical properties of ligated gold clusters are strongly defined by the metal–ligand interaction and type of ligands. This critical feature renders gold–phosphine clusters unique and distinct from other ligand‐protected gold clusters. The use of multidentate phosphines enables preparation of varying core sizes and exotic structures beyond regular polyhedrons. Weak gold–phosphorous (Au–P) bonding is advantageous for ligand exchange and removal for specific applications, such as catalysis, without agglomeration. The aim of this review is to provide a unified view of gold–phosphine clusters and to present an in‐depth discussion on recent advances and key developments for these clusters. This review features the unique chemistry, structural, electronic, and optical properties of gold–phosphine clusters. Advanced characterization techniques, including synchrotron‐based spectroscopy, have unraveled substantial effects of Au–P interaction on the composition‐, structure‐, and size‐dependent properties. State‐of‐the‐art theoretical calculations that reveal insights into experimental findings are also discussed. Finally, a discussion of the application of gold–phosphine clusters in catalysis is presented.

## Graphical abstract

Many unique properties of ligated gold clusters are defined by the metal–ligand interaction and type of ligand. These critical features render gold–phosphine clusters distinct from other ligated clusters. The gold–phosphorous bond gives rise to a rich chemistry that is manifested in synthesis, ligand exchange and reactivity, as well as remarkable physical properties such as unique geometry, fluxionality, chirality, and stability.

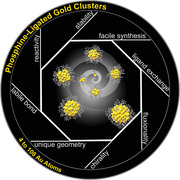

## 1 Introduction

Atomically precise gold (Au) clusters can be readily controlled in the assembly of nanomaterials and therefore have become a major focus in cluster chemistry research. Furthermore, their atomic precision permits structure–property relationships to be clearly determined. Due to their ultrasmall size, the properties and behaviors of gold clusters are remarkably different from individual atoms and bulk gold.^[^ ^1^ ^]^ Crucially, the size‐dependent properties of gold clusters offer the advantage of being able to study the effects of “one atom makes a difference” in fundamental science and applications.^[^ ^2^ ^]^ For ligated gold clusters, the metal–ligand interaction and bonding largely define and govern the chemical, physical, electronic, and optical properties. Hence, such features distinguish phosphine‐ligated from alkynyl‐protected or thiolate‐protected gold clusters. The nature of phosphine ligands determines the core size nuclearity, geometrical structure, fluxionality, chirality, stability, reactivity, and solubility leading to the rich chemistry of gold–phosphine clusters.

The metal–ligand bonds in gold–phosphine clusters are dative covalent bonds where the lone pair of electrons in phosphorous atoms contribute to Au–P bonding. The remarkable simplicity of Au–P bonds is owing to phosphine ligands having no terminal or bridging coordination modes. Early molecular orbital calculations by Mingos revealed that ligation with phosphines promotes hybridization of gold orbitals due to the contribution from the lone pair of electrons and results in stronger radial Au–Au bonding.^[^ ^3^ ^]^ Interestingly, Mingos et al. showed that small changes in phosphine ligands lead to drastic changes in structural and the physical properties of gold–phosphine clusters. For example, clusters of the same nuclearity and charge state, Au_6_(P(*p*‐tol)_3_)_6_^2+^ and Au_6_(PPh_3_)_6_^2+^, adopt octahedral and edge‐shared tetrahedral structures, respectively.^[^ ^4^ ^]^ Such differences raise intriguing questions about the relative stability and activation barrier of the two structures.

Since the first preparation and structural determination of Au_11_(PPh_3_)_7_(SCN)_3_ by Malatesta et al.,^[^ ^5^ ^]^ numerous other Au clusters have been prepared using monodentate and multidentate phosphine ligands.^[^ ^6^ ^]^ Striking and appealing features of gold–phosphine clusters include facile synthesis, simple purification, and ease of crystallization. In contrast to many thiolate‐protected Au clusters that require difficult and expensive purification^[^ ^7^ ^]^ that usually employs polyacrylamide gel electrophoresis (PAGE),^[^ ^8^ ^]^ size exclusion,^[^ ^9^ ^]^ or liquid chromatography,^[^ ^10^ ^]^ many gold–phosphine clusters can be simply purified by filtration, washing, precipitation or simple crystallization.^[^ ^6^, ^11^ ^]^ In some cases, crystallization serves as a dual role to purify and grow crystals. The ease of crystallization is advantageous for gold–phosphine clusters and contributes to rapid developments in their synthesis and single‐crystal X‐ray studies.^[^ ^12^ ^]^ A metathesis reaction of the counter anions with PF_6_^−^, BF_4_^−^ or SbF_6_^−^ can be applied to facilitate crystallization of clusters that are otherwise difficult to grow into high‐quality crystals.

In contrast to strong Au–S bonding, Au–P bonding is relatively weaker. Thus, gold–phosphine clusters have remarkably different chemistry, structural and physical properties to thiolate‐protected Au clusters. Labile Au–P bonding offers benefits in ligand exchange and removal. Ligand exchange with other organic ligands allows preparation of mixed‐ligand compositions and the study of their reactivity and stability.^[^ ^13^ ^]^ Importantly, it is a particularly useful process for introducing functionality in gold clusters for specific applications. The reactivity of gold–phosphine clusters has also been exploited to prepare new thiolate‐protected clusters via ligand exchange.^[^ ^14^ ^]^ Ligand removal is a critical step in activation of gold‐based catalysts. Removal of phosphine ligands from the gold core is readily achieved under mild conditions due to the labile Au–P bonding. For instance, Anderson et al. investigated the removal of triphenylphosphines in various PPh_3_‐ligated Au*_n_* (*n* = 8,9,11,101) clusters by calcination at moderate temperature (≤ 200 °C) under different conditions and chemical etching.^[^ ^15^ ^]^

Another exciting feature of gold–phosphine clusters is fluxionality. Fluxionality is the fast, dynamic intramolecular rearrangement of chemically equivalent configurations due to the stereochemical nonrigidity and low energy barrier between different configurations.^[^ ^16^ ^]^ The weaker peripheral Au–Au bonds compared to the radial Au–Au bonds give rise to nonrigidity and thus flexible skeletal rearrangements.^[^ ^17^ ^]^ This observation also suggests that Au–Au bonds have soft potential energy surfaces.^[^ ^18^ ^]^ Theoretical calculations showed that structural fluxionality determines the catalytic activity of clusters via substrate adsorption.^[^ ^19^ ^]^ Spectroscopic evidence for fluxional behavior is often manifested by the presence of only a single resonance in ^31^P[^1^H] solution NMR spectra of many monophosphine‐ligated Au clusters.^[^ ^16^, ^20^ ^]^ It was first observed that Au_9_(P(p‐C_6_H4OMe)_3_)_8_ produced two skeletal isomers (butterfly and crown structures) upon crystallization.^[^ ^21^ ^]^ Structural isomerization can be induced by evaporation with anions (NO_3_^−^, Cl^−^, Keggin) or by applying pressure.^[^ ^22^ ^]^ More recently, it has been shown that structural isomerization can be hindered by doping the Au_9_ cluster with a single Pd atom which is due to induced bond stiffening.^[^ ^23^ ^]^

Advances in characterizations allow precise determination of extraordinary properties of gold clusters and development of structure–property relationships. For example, the accurate structure of Schmid's Au_55_ (first synthesized in 1981) has finally been resolved using aberration‐corrected scanning tunneling electron microscope in 2015.^[^ ^24^ ^]^ The use of synchrotron‐based spectroscopy such as far‐infrared, X‐ray photoelectron (XPS), and absorption (XAS) spectroscopy provides information about the nature of metal–ligand interaction. High‐resolution, high‐sensitivity mass spectrometry has revealed the reaction pathways in cluster synthesis by identifying the corresponding initial species and intermediates responsible for formation of size‐selective clusters. The characterization techniques discussed in this review provide a complete, unified view of the size‐, structure‐ and composition‐dependent properties of gold–phosphine clusters.

The continuous progress in density functional theory (DFT) and improvement of high‐performance computing (HPC) paved the way for the quantum chemical simulations of the different ground‐state properties of phosphine‐stabilized gold clusters. Calculations of the geometric and electronic structures, vibrational modes, and bond dissociation energies complement those observed experimentally, thus providing an atomic‐level insight into these observed properties. Likewise, calculations of optical properties and simulations of steady‐state absorption and fluorescence spectra enable excited state dynamics studies of these Au clusters, thus advancing the understanding of optical properties such as the effect of ligands on spectral shifts and phosphorescence. The section dedicated to quantum chemical simulations of phosphine‐stabilized Au clusters reviews several studies that have reported key theoretical findings with respect to different properties of gold–phosphine clusters.

Research on atomically precise gold–phosphine clusters bloomed between 1970 and the mid‐1990s. Thereafter it was forgotten as attention shifted to thiolate‐protected Au clusters. The revival of interest in gold–phosphine clusters is due to the work of many researchers in diverse areas, such as catalysis by supported Au clusters, spectroscopic and microscopic characterizations, and theoretical calculations. In particular, the work of Simon and co‐workers in reproducing some of these clusters and providing complete characterizations led to a revival in interest in gold–phosphine clusters.^[^ ^25^ ^]^ Laskin's group utilizes mass spectrometry to investigate phosphine ligand effects in gold clusters.^[^ ^26^ ^]^ Pettibone and Hudgens have recently made significant breakthroughs in defining the reaction pathways and mechanism in diphosphine‐ligated Au cluster systems.^[^ ^27^ ^]^ Häkkinen and co‐workers have provided theoretical and computational descriptions of the electronic structures, doping, and catalysis of Au clusters.^[^ ^28^ ^]^

It is important to recognize that apart from phosphine ligands, gold clusters can also be stabilized by other ligands such as thiolates. In general, the synthesis of many phosphine‐ligated Au clusters is relatively simple; the synthesis often involves direct chemical reduction of gold precursors (e.g., Au(PPh_3_)Cl, Au(PPh_3_)NO_3_) without the addition of a phase transfer agent (TOABr) or an extra ligand that is often employed in thiolate‐protected counterparts. Importantly, manipulation of reaction conditions (solvent, precursor/reduction ratio, reducing agent) allows the synthesis of different clusters (refer to **Table** **1** in subsection 2.1). The presence of phenyl rings in phosphine ligands (due to the CH–*π* interaction) facilitates crystallization into single crystals for structural determination. Diverse gold–sulfur (Au–S) bonding motifs are ubiquitous in thiolate‐protected systems while direct gold–phosphorous (Au–P) bonding dominates gold–phosphine clusters. Häkkinen pointed out that the strength of the Au–S bond is comparable to that of the Au–Au bond.^[^ ^29^ ^]^ The stronger Au–S bond gives enhanced stability of thiolate‐protected clusters in solution and on surfaces while labile Au–P bonding is the basis for the enhanced reactivity of gold–phosphine clusters toward a variety of substrates, in ligand exchange or removal, and in intercluster conversion. Dynamical aspects such as fluxionality and ligand mobility, and chirality have been observed in both types of clusters. However, the energetics and mechanism are different and must be evaluated case‐by‐case.

**Table 1:** Summary of the synthesis of different phosphine‐ligated Au clusters reported to date

Gold clusters	Precursor	Reducing/additive agent	Solvent	Refs.
Au_4_(PPh_3_)_4_(µ‐I)_2_	Au_9_(PPh_3_)_8_(NO_3_)_3_	KI	Me_2_CO	[6e]
Au_5_(dppm^−^)^a)^_3_(dppm)(NO_3_)_2_	Au_2_(dppm)(NO_3_)_2_, dppm	NaBH_4_	EtOH	[6g]
Au_2_Ag_2_(PPh_3_)_2_(C_10_H_6_NO)_4_	Au(C_10_H_6_NO), Ag(C_10_H_6_NO)	PPh_3_	DCM/MeOH	[39]
[Au_3_Ir(PPh_3_)_5_(NO_3_)]PF_6_	Au(PPh_3_)NO_3_, [Ir_2_(µ‐H)_3_(H)_2_(PPh_3_)_4_]PF_6_		Me_2_CO	[40]
[Au_4_Ir(H)_2_(PPh_3_)_6_]BF_4_	[Au_3_Ir(PPh_3_)_5_(NO_3_)]BF_4_	H_2_	DCM	[41]
Ru_3_Au(PPh_3_)Cl(CO)_10_	Au(PPh_3_)Cl, Ru_3_(CO)_12_		DCM	[42]
Au_4_Ru_2_(PPh_3_)_2_(SR)_8_	Au(PPh_3_)Cl, Ru(PPh)_3_Cl_2_, SR	NaBH_4_	EtOH	[43]
[Au_5_Re(H)_4_(PPh_3_)_7_](PF_6_)_2_	Au(PPh_3_)NO_3_, ReH_7_(PPh_3_)_2_		DCM	[44]
Au_5_Cu_6_(dppf)_2_(SR)_6_BPh_4_	HAuCl_4_, CuCl_2_, dppf, SR	BTBA	MeOH	[45]
Au_6_(PPh_3_)_6_(NO_3_)_2_	Au_8_(PPh_3_)_8_(NO_3_)_2_	K[Ag(CN)_2_]	MeOH	[6f]
[Au_6_(Ph_3_)_6_]^2+^	Au(PPh_3_)Cl	NaBH_4_/NH_3_ solution	EtOH	[46]
PdAu_6_(PPh_3_)_7_(NO_3_)_2_	Au(PPh_3_)NO_3_, PdCl_2_(PPh_3_)_2_	NaBH_4_	DCM/MeOH	[47]
PtAu_6_(PPh_3_)_7_(NO_3_)_2_	[PtAu_2_(PPh_3_)_4_(NO_3_)]NO_3_	H_2_	DCM	[48]
Au_6_(dppp)_4_(NO_3_)_2_	Au_9_(PPh_3_)_8_(NO_3_)_3_	dppp	DCM	[6h]
[Au_7_(PPh_3_)_7_]^+^	Au metal	PPh_3_	C_7_H_8_	[11b]
[Au_7_(dppp)_4_](BF_4_)_3_	Au_6_(dppp)_4_(BF_4_)_2_	AgBF_4_ or AgNO_3_	MeOH	[49]
Au_8_(PPh_3_)_8_(NO_3_)_2_	Au_9_(PPh_3_)_8_(NO_3_)_3_	PPh_3_	DCM	[11]^a)^
Au_8_(PPh_3_)_7_(NO_3_)_2_	Au_8_(PPh_3_)_8_(NO_3_)_2_	[RhC1(C_8_H_14_)_2_]_2_	DCM	[11a,c]
[Au_8_(PPh_3_)_7_]^2+^	Au(PPh_3_)_2_Cl	NaBH_4_	DCM	[50]
[Au_8_(PPh_3_)_6_I]PF_6_	Au_9_(PPh_3_)_8_(NO_3_)_3_	Bu_4_NI	MeOH	[11b]
[Au_8_(dppp)_4_](NO_3_)_2_	Au_9_(PPh_3_)_8_(NO_3_)_3_	dppp	DCM/C_7_H_8_	[51]
[Au_8_(dppp)_4_Cl_2_](NO_3_)_2_	Au_6_(dppp)_4_(NO_3_)_2_	Au(PPh_3_)Cl	MeOH/CHCl_3_	[51]
Au_9_(PPh_3_)_8_Cl	Au(PPh_3_)_3_Cl	PPh_3_BH_3_	MeOH/CH_2_Cl_2_	[52]
Au_9_(PPh_3_)_8_(NO_3_)_3_	Au(PPh_3_)NO_3_	NaBH_4_	EtOH	[11a]
Au_9_(PPh_3_)_8_(BF_4_)_3_	Au(PPh_3_)Cl	Ti(*η*‐C_7_H_8_)_2_	C_7_H_8_/EtOH	[35a]
[Au_9_H(PPh_3_)_8_]^2+^	Au_9_(PPh_3_)_8_(NO_3_)_3_	NaBH_4_	EtOH	[53]
[Au_9_Ag_12_(dppm)_6_Cl_6_(SR)_4_]^3+^	HAuCl_4_, AgNO_3_, dppm, SR	NaBH_3_CN	MeOH	[54]
PdAu_8_(PPh_3_)_8_Cl_2_	Au(PPh_3_)Cl, Pd(PPh_3_)_4_	NaBH_4_	EtOH	[55]
PdAu_8_(PPh_3_)_8_(NO_3_)_2_	Au(PPh_3_)NO_3_, Pd(PPh_3_)_4_	NaBH_4_	DCM/EtOH	[56]
PtAu_8_(PPh_3_)_8_(NO_3_)_2_	Au(PPh_3_)NO_3_, Pt(PPh_3_)_3_	H_2_	Me_2_CO	[57]
Au_8_Ag_3_(PPh_3_)_7_Cl_3_	Au(PPh_3_)Cl, AgSbF_6_	NaBH_4_	DCM/MeOH	[58]
Au_8_Ag_17_(PPh_3_)_10_Cl_10_	AuCl_3_, AgNO_3_	NaBH_4_	DCM/MeOH	[59]
[Au_8_Ag_57_(dppp)_4_(SR)_32_Cl_2_]Cl	HAuCl_4_, AgNO_3_, dppp, SR	NaBH_4_ or BTBA	MeOH/DCM	[60]
Au_10_(PPh_3_)_5_(C_6_F_5_)_4_	Au_9_(PPh_3_)_8_(NO_3_)_3_	NBu_4_[Au(C_6_F_5_)_2_]	DCM	[61]
Au_10_(PPhCy_2_)_6_Cl_3_(NO_3_)	Au(PPhCy_2_)(NO_3_)	NaBH_4_	EtOH	[62]
Au_10_(PPh_3_)_7_[(S_2_C_2_(CN)_2_]_2_	Au_9_(PPh_3_)_8_(NO_3_)_3_	Na_2_S_2_C_2_(CN)_2_	MeOH	[63]
PdAu_10_(PPh_3_)_8_Cl_2_	Au(PPh_3_)Cl, Pd(PPh_3_)_4_	NaBH_4_	EtOH	[64]
[PdAu_9_(TFPP)_7_Br_2_]^+^	HAuCl_4_, H_2_PdCl_4_, TFPP	NaBH_4_	MeOH/DCM	[65]
[HPdAu_10_(PPh_3_)_8_Cl_2_]Cl	PdAu_8_(PPh_3_)_8_(NO_3_)_2_, Au(PPh_3_)Cl	NaBH_4_	EtOH/THF	[56]
[PtAu_10_(PEt_3_)_10_](PF_6_)_2_	Au(PEt_3_)NO_3_, Pt(PEt_3_)_3_	H_2_	THF	[66]
Au_11_(PPh_3_)_7_Cl_3_	Au(PPh_3_)Cl	NaBH_4_	EtOH	[25b]
Au_11_(PPh_3_)_7_Cl_3_	Au(PPh_3_)Cl	NaBH_4_	THF	[67]
Au_11_(PPh_3_)_7_I_3_	Au metal	PPh_3_	C_7_H_8_	[11d]
Au_11_(PPh_3_)_7_Br_3_	HAuCl_4_.xH_2_O, PPh_3_	NaBH_4_	EtOH/C_7_H_8_	[68]
Au_11_(PPh_2_Py)_7_Br_3_	Au(PPh_2_Py)Cl	NaBH_4_	EtOH	[69]
Au_11_(PPh_3_)_8_Cl_3_	Au(PPh_3_)Cl	NaBH_4_	EtOH	[70]
Au_11_(PPh_3_)_8_Cl_3_	Au(PPh_3_)Cl	NaBH_4_	DCM	[67]
Au_11_(PMe_2_Ph)_10_(PF_6_)_3_	Au(PMe_2_Ph)Cl	Ti(*η*‐C_7_H_8_)_2_	C_7_H_8_/EtOH	[35a]
Au_11_(BINAP)_4_(C≡CPh)_2_Cl	Au(SMe_2_)Cl, BINAP, HC≡CPh	NaBH_4_	DCM/MeOH	[71]
[Au_11_(DPEphos)_4_Cl_2_]Cl	Au_2_(DPEphos)Cl_2_	NaBH_4_	MeOH	[72]
[Au_11_(Xantphos)_4_Cl_2_]Cl	[Au_2_(Xantphos)Cl_2_	NaBH_4_	MeOH	[72]
[Au_11_(dppp)_5_]^3+^	Au_2_(dppp)Cl_2_	NaBH_4_	EtOH	[73]
Au_11_(dppe)_6_(SbF_6_)_3_	Au_2_(dppe)Cl_2_	NaBH_4_	EtOH	[74]
[Au_11_Cu(PPh_3_)_7_(SPy)_3_]^+^	Au(PPh_3_)Cl, Cu(OAc)_2_, Cu(NO_3_)_2_, SPy, PPh_3_	NaBH_4_	MeOH/DCM	[75]
[PtAu_12_(dppe)_5_Cl_2_](PF_6_)_2_	Au_2_(dppe)(NO_3_)_2_, Pt(cod)(NO_3_)_2_	NaBH_4_	THF/EtOH	[76]
[IrAu_12_(dppe)_5_Cl_2_]Cl	Au_2_(dppe)Cl_2_, [Ir(cod)Cl]_2_	NaBH_4_	DCM/EtOH	[76]
Au_12_Ag_7_(PMe_2_Ph)_10_(NO_3_)_9_	Au(PMe_2_Ph)(NO_3_), AgNO_3_	NaBH_4_	EtOH	[77]
[PtAg_12_Au_12_(PPh_3_)_10_Cl_7_]Cl	Au_8_Pt(PPh_3_)_8_(NO_3_)_2_, Ag_4_(PPh_3_)_4_Cl_4_	NaBH_4_	EtOH	[78]
[Pt_3_Ag_21_Au_12_(PPh_3_)_12_Cl_8_]^+^	Au(PPh_3_)Cl, Pt_3_Ag_33_(PPh_3_)_12_Cl_8_]^+^		DCM	[79]
[Au_13_(PMe_2_Ph)_10_Cl_2_](PF_6_)_3_	[Au_11_(PPhMe_2_)_10_]^3+^	NEt_4_Cl	EtOH	[38]
[Au_13_(dppe)_5_Cl_2_]Cl_3_	Au_2_(dppe)Cl_2_	NaBH_4_	DCM/EtOH	[80]
[Au_13_(dppm)_6_]Cl_5_	Au_2_(dppm)_2_Cl_2_	NaBH_4_	DCM/MeOH	[81]
[PdAu_13_(PPh_3_)_3_(SR)_7_]^+^	HAuCl_4_, (NH_4_)_2_PdCl_4_	NaBH_4_	C_7_H_8_/H_2_O/ MeOH	[82]
[Au_13_Cu_4_(PPh_3_)_4_(SPy)_8_]^+^	Au(PPh_3_)Cl, Cu(OAc)_2_, SPy	NaBH_4_	DCM/MeOH	[83]
[Au_13_Ag_12_(PPh_3_)_10_Cl_8_]SbF_6_	Au(PPh_3_)Cl, AgSbF_6_	NaBH_4_	CH_3_Cl/MeOH	[84]
Au_14_(PPh_3_)_8_(NO_3_)_4_	Au(PPh_3_)NO_3_	NaBH_4_	EtOH	[85]
[Au_18_(dppm)_6_Cl_4_]Cl_3_PF_6_	Au_2_(dppm)_2_Cl_2_	NaBH_4_/ ^n^BuNPF_6_	THF/MeOH	[86]
[Au_18_Ag_20_(PPh_3_)_14_Cl_12_]Cl_2_	HAuCl_4_, AgSbF_6_	NaBH_4_	EtOH	[87]
Au_19_(BINAP)_4_(C≡CPh)Cl_4_	HAuCl_4_.4H_2_O, BINAP, HC≡CPh	NaBH_4_	DCM/MeOH	[71]
[Au_19_(C≡CPh)_9_(Hdppa)_3_](SbF_6_)_2_	AuC≡CPh, Au_2_Hdppa(SbF_6_)_2_	NaBH_4_	DCM/EtOH	[88]
[Au_19_Cu_30_(C≡CPh)_22_(PPh_3_)_6_Cl_2_](NO_3_)_3_	PhC≡CAu(PPh_3_), CuCl, Cu(NO_3_)_2_	NaBH_4_	DCM/EtOH	[89]
[Au_20_(PPh_3_)_8_]^2+^	Au(PPh_3_)Cl	NaBH_4_	C_7_H_8_/H_2_O	[90]
Au_20_(PP_3_)_4_Cl_4_	Au_4_PP_3_Cl_4_	NaBH_4_	DCM/EtOH	[91]
[Au_20_(PPhpy_2_)_10_Cl_4_]Cl_2_	Au(PPhpy_2_)Cl	NaBH_4_	EtOH	[92]
[Au_20_(PPh_3_)_12_H_3_](SbF_6_)_3_	Au(PPh_3_)SbF_6_	NaBH_4_/EtONa/2,2‐bpa	DCM/EtOH	[20b]
Au_22_(dppo)_6_	Au_2_(dppo)Cl_2_	NaBH_4_	DCM/EtOH	[93]
Au_22_(dppee)_7_	Au_2_(dppee)Cl_2_	NaBH_4_	DCM/EtOH	[94]
[Au_22_H_3_(dppee)_7_]^3+^	Au_2_(dppee)Cl_2_	NaBH_4_	DCM/EtOH	[95]
[Au_22_H_4_(dppo)_6_]^2+^	Au_2_(dppo)Cl_2_	NaBH_4_	DCM/EtOH	[96]
[Au_23_(PPh_3_)_6_(C≡CPh)_9_](SbF_6_)_2_	Au(PPh_3_)SbF_6_, AuC≡CPh	NaBH_4_	DCM/EtOH	[97]
[Au_24_(PPh_3_)_4_(C≡CPh)_14_](SbF_6_)_2_	Au(PPh_3_)SbF_6_, AuC≡CPh	NaBH_4_	CHCl_3_/MeOH/EtOH	[98]
[Au_24_(dppb)_6_Cl_4_]Cl_2_	Au_2_(dppb)Cl_2_	NaBH_4_/N(CH_3_)_4_Cl	EtOH	[99]
[Au_24_Pd(PPh_3_)_10_(SR)_5_Cl_2_]Cl	Au(PPh_3_)Cl, Pd(PPh_3_)_4_, SR	NaBH_4_	EtOH	[100]
[Au_25_(PPh_3_)_10_(SR)_5_Cl_2_](SbF_6_)_2_	[Au_11_(PPh_3_)_8_Cl_2_]Cl	SR	CHCl_3_	[101]
AuCu_24_H_22_(PPh_3_)_12_	HAuCl_4_, Cu(acac)	NaBH_4_	MeOH/DCM	[102]
Pd_2_Au_23_(PPh_3_)_10_Br_7_	HAuBr, PdCl_2_	NaBH_4_	H_2_O/C_7_H_8_	[103]
Pd_2_Au_23_(TFPP)_10_Br_7_	[PdAu_9_(TFPP)_7_Br_2_]^+^		DCM	[65]
Pd_28_Au_2_(CO)_26_(PEt_3_)_10_	Au(SMe_2_)Cl, Pd_10_(CO)_12_(PEt_3_)_6_		Me_2_CO/HOAc	[104]
[Au_28_(PPh_3_)_9_(SR)_4_]^2+^	Au(PPh_3_)Cl, SR	NaBH_4_	THF/H_2_O	[105]
Au_32_(PR_3_)_12_Cl_8_ ^b)^	Au(PR_3_)Cl^b)^	NaBH_4_	EtOH	[106]
[Au_32_(PPh_3_)_8_(dpa)_6_](SbF_6_)_2_	Au(PPh_3_)_2_SbF_6_, Au(dpa), MeONa	NaBH_4_	DCM/MeOH	[107]
[Au_37_(PPh_3_)_10_(SR)_10_Cl_2_]^+^	HAuCl_4_, PPh_3_, SR	NaBH_4_	H_2_O/C_7_H_8_	[108]
[Au_39_(PPh_3_)_14_Cl_6_]Cl_2_	HAuCl_4_, PPh_3_	NaBH_4_	EtOH	[109]
[Au_40_(dppm)_4_(C≡CPh)_20_](SbF_6_)_4_	dppm(AuO_2_C_2_F_3_)_2_, AuC≡CPh	NaBH_4_	DCM/EtOH	[110]
Au_54_(PEt_3_)_18_Cl_12_	Et_3_PAuCl	NaBH_4_	EtOH	[37]
Au_55_(PPh_3_)_12_Cl_6_ ^c)^	Au(PPh_3_)Cl	B_2_H_6_ gas	C_6_H_6_	[111]
Au_70_(PPh_3_)_12_S_20_	(Ph_3_P)AuSC(SiMe_3_)_3_	L‐Selectride	Et_2_O	[112]
Au_101_(PPh_3_)_21_Cl_5_ ^c)^	HAuCl_4_, PPh_3_	NaBH_4_	C_7_H_8_/H_2_O	[113]
Au_108_(PPh_3_)_16_S_24_	Au(PPh_3_)Cl, HSC(SiMe_3_)_3_	NaBH_4_	THF/H_2_O	[114]

^a)^ dppm^−^ anion is a dppm ligand with a loss of one proton;

^b)^ PR_3_ = PEt_3_, ^n^PPr_3_, ^n^PBu_3_;

^c)^ Not atomically‐precise but average formula.

Currently, there are numerous review papers that discuss thiolate‐protected atomically precise Au clusters, particularly by Jin et al.,^[^ ^1^, ^30^ ^]^ Tsukuda et al.,^[^ ^31^ ^]^ Chakraborty and Pradeep,^[^ ^32^ ^]^ and others.^[^ ^28^, ^33^ ^]^ However, we are not aware of any in‐depth reviews that exclusively and comprehensively consider phosphine‐ligated atomically precise gold clusters.^[^ ^34^ ^]^ Brief reviews describing the synthetic methods and crystal structures of phosphine‐ligated Au clusters were provided in the early 1980s by Hall and Mingos^[^ ^18^, ^35^ ^]^ and Steggerda et al.,^[^ ^36^ ^]^ predominantly on monophosphine‐stabilized Au clusters. While the first research dates back to the 1970s, new findings from experimental and theoretical studies of gold–phosphine clusters continue to reveal novel characteristics. Over almost four decades there has been a tremendous amount of new work reported in the literature, ranging from synthesis and characterization methods to theoretical calculations and applications and so it is timely to present an updated review on recent progress and key developments in gold–phosphine clusters.

In this review, we first present an overview, general features, and the merits of gold–phosphine clusters. In Section 2, we cover the chemistry of these Au clusters including synthetic methods, etching process, and ligand exchange with other ligands. Next, various properties, including electronic structures, optical (absorption and photoluminescence) properties, and chirality, are discussed. State‐of‐the‐art characterization tools, including synchrotron techniques, are discussed so that the reader may understand the structure–property relationships described in the following section. In Section 5, we provide an in‐depth discussion on quantum chemical calculations of the structures and properties of gold–phosphine clusters. The quantum chemical calculations reveal that Au–P bonding has substantial effects in determining the geometry and governing the electronic structures, charge transfer, metal–ligand interface, and optical properties. Finally, we highlight applications of these clusters in catalysis ranging from oxidation and hydrogenation reactions to photocatalysis. To close, we present a perspective on the challenging issues and opportunities for future work. This review article covers the scientific literature from 1969 until October 2021.

## 2 Chemistry of Phosphine‐Ligated Au Clusters

### 2.1 Synthetic Methods

Wet chemical synthesis is often preferred by chemists because it only requires a simple and cost‐effective set up and gives relatively high yields. Typically, this method requires ligands to stabilize the metal core against aggregation in solution and solid state. In general, there are three synthetic methods used to prepare phosphine‐ligated Au clusters: reduction of gold precursor compounds, intercluster conversion of Au clusters, and metallic Au evaporation. The former two methods are widely used owing to their simplicity and scalability. The latter is hardly used nowadays even though it generally produces high yields. Table 1 summarizes preparations of gold–phosphine clusters including precursors, reducing agents, and solvents reported to date.

It is noticeable from Table 1 that triphenylphosphine (PPh_3_) tends to form low nuclearity clusters (Au*_n_*, *n* < 13) except for some large clusters such as Au_39_, Au_55,_ and Au_101_. Notably, both Au_55_ and Au_101_ clusters are not truly atomically precise in nature. Larger clusters can be made with the use of multidentate phosphines or in combination with thiols owing to the stronger bonding via multiple chelating sites or the Au–S bond, respectively. More recently, seminal works by Kenzler et al. showed that it is feasible to synthesize large nuclearity Au clusters using less steric trialkylphosphine. The authors noted that reduction of the precursor AuPEt_3_Cl by NaBH_4_ afforded two different clusters, with Au_32_ and Au_54_ cores, depending on the work‐up (extraction and layering) procedure.^[^ ^20^, ^37^ ^]^ Previously, Mingos and co‐workers successfully achieved tridecagold using the less steric PMePh_2_ ligand.^[^ ^38^ ^]^ Understanding the nature of ligands provides insights for rational design of size‐selective Au clusters, and their structure and properties. These works motivated further investigations using other precursors and/or ligands to achieve novel clusters.

Reduction of Au compounds, HAuCl_4_ and Au(PPh_3_)X (where X = Cl^−^, I^−^, CN^−^, SCN^−^, NO_3_^−^), by sodium borohydride (NaBH_4_) is the oldest and simplest chemical synthesis of gold–phosphine clusters. The simplicity of this method makes it attractive for the preparation of various size‐selective gold–phosphine clusters.^[^ ^85^, ^90^ ^]^ The nature of the final Au clusters obtained depends on the type of precursor, type of anion and ratio of reducing agent to gold precursor. In the case of triarylphosphine‐ligated Au clusters, while the complete mechanism of the formation of size‐selective Au clusters is not fully understood, there exists a general trend that makes the prediction of synthesis of Au clusters useful. For example, reduction of Au(I) compounds containing coordinating anions (X = I^−^, CN^−^, SCN^−^) by NaBH_4_ favors the formation of undecagold with the generic formula Au_11_L_7_X_3_, while noncoordinating anions (X = NO_3_^−^, ClO_4_^−^, PF_6_^−^) afford nonagold [Au_9_L_8_]^3+^.^[^ ^115^ ^]^

Reducing agents strongly influence the formation of final clusters. While NaBH_4_ is ubiquitous in cluster synthesis, other less common reducing agents have also been explored. Di(toluene)titanium [Ti(*η*‐C_7_H_8_)_2_] was found to be an effective reducing agent that produced yields over 80%.^[^ ^116^ ^]^ Notably, the reduction of Au(PPh_3_)Cl by Ti(*η*‐C_7_H_8_)_2_ affords Au_9_(PPh_3_)_8_^3+^ as opposed to Au_11_(PPh_3_)_7_^3+^ obtained by NaBH_4_ reduction. Weak reducing agents such as borane‐*tert*‐butylamine (BTBA) and 9‐borabicyclo[3.3.1]nonane (9‐BBN) have also been employed in cluster synthesis.^[^ ^26^, ^117^ ^]^ Kenzler et al. employed L‐selectride (LiBH(sec‐Bu)_3_) in the synthesis of a large Au_70_(PPh_3_)_12_S_20_ cluster, extending the borane‐type reducing agents.^[^ ^112^ ^]^ However, the minute yield (1–1.5%) becomes means its use is not prevalent. The same group also reported a novel bimetallic [Au_9_Ga(PPh_3_)_8_Cl_2_]^2+^ cluster using gallium cyclopentadienyl (GaCp) that serves as a reducing agent and a gallium source.^[^ ^118^ ^]^ The successful synthesis of new clusters encourages further exploration of new reducing agents, which in turn expands the range of possible gold–phosphine clusters.

**Figure 1:**
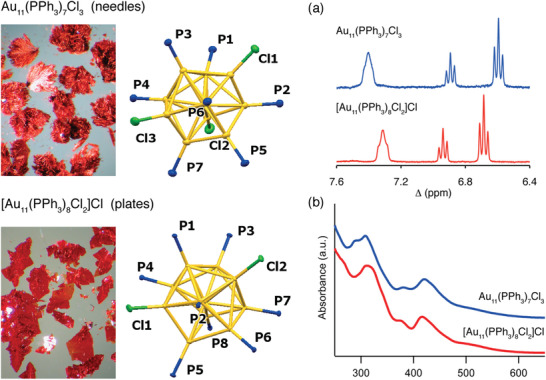
High quality crystals of Au_11_(PPh_3_)_7_Cl_3_ (orange needles) and [Au_11_(PPh_3_)_8_Cl_2_]Cl (red plates) clusters together with their corresponding a) ^1^H NMR spectra and b) UV–visible absorption spectra. Reproduced with permission.^[^ ^67^ ^]^ Copyright 2014, American Chemical Society.

Undecagold was first reported in 1969, however the lack of precise structural determination led to several proposed formulae such as Au_11_(PPh_3_)_7_Cl_3_, Au_11_(PPh_3_)_8_Cl_3,_ and [Au_11_(PPh_3_)_8_Cl_2_]^+^ until the structure was determined by Simon and co‐workers in 2013.^[^ ^25b^ ^]^ This discrepancy results from the formation of isostructural undecagold clusters, Au_11_(PPh_3_)_7_Cl_3_ and [Au_11_(PPh_3_)_8_Cl_2_]Cl, using the same synthetic method.^[^ ^70^, ^119^ ^]^ Recently, Hutchison and co‐workers showed that this synthetic protocol produces a mixture of Au_11_(PPh_3_)_7_Cl_3_ and [Au_11_(PPh_3_)_8_Cl_2_]Cl, and the separation of the components is difficult.^[^ ^67^ ^]^ Such difficulty renders crystallization difficult and leads to ambiguous structural characterization by single‐crystal XRD. The authors outlined the synthetic protocols to obtain different undecagold clusters directly via reduction of Au(PPh_3_)Cl with NaBH_4_ by varying the amount of NaBH_4_ and type of solvents used. The use of tetrahydrofuran (THF) as a solvent and fivefold excess of NaBH_4_ in ethanol affords exclusively Au_11_(PPh_3_)_7_Cl_3_ while the use of 0.25 equivalent NaBH_4_ in ethanol and dichloromethane yields [Au_11_(PPh_3_)_8_Cl_2_]Cl. **Figure** **1** shows high‐quality crystals grown separately which produce orange needles and red plates corresponding to isostructural Au_11_(PPh_3_)_7_Cl_3_ and [Au_11_(PPh_3_)_8_Cl_2_]Cl clusters, respectively.

While synthesis in one‐phase solution offers simplicity and cost‐effectiveness, sometimes it is not possible to use this method for other clusters due to inevitable constraints in synthetic chemistry and thus, biphasic solution synthesis becomes a viable alternative. Wu and Jin first reported a biphasic synthesis of the Au_11_(PPh_3_)_8_Br_3_ cluster via reduction of HAuCl_4_ by NaBH_4_ in an ethanol–toluene solution.^[^ ^68^ ^]^ Tetraoctylammonium bromide (TOAB) was used as a phase‐transfer reagent and a supply of Br^−^ anions producing intermediate Au(PPh_3_)Br that led to the exclusive formation of Au_11_(PPh_3_)_8_Br_3_ clusters owing to their thermodynamic stability. Similar reactions using this method have been employed to prepare [Au_20_(PPh_3_)_8_]^2+^ and Au_101_(PPh_3_)_21_Cl_5_ clusters previously.^[^ ^90^, ^113^ ^]^

A successful preparation of the [Au_8_(PPh_3_)_7_]^2+^ cluster via direct synthesis was demonstrated by Huang et al.^[^ ^50^ ^]^ Reduction of Au(PPh_3_)_2_Cl in DCM by NaBH_4_ over 168 hours produced exclusively octagold [Au_8_(PPh_3_)_7_]^2+^ as opposed to undecagold [Au_11_(PPh_3_)_8_Cl_2_]^+^ obtained from Au(PPh_3_)Cl. Such a difference demonstrates a critical role of Au precursors in cluster synthesis. The same group also reported a novel method to synthesize the [Au_6_(Ph_3_)_6_]^2+^ cluster by introducing ammonia etching after the reduction of Au(PPh_3_)Cl by NaBH_4_.^[^ ^46^ ^]^ Ammonia acts to remove [Au(PPh_3_)]^+^ fragments in larger Au clusters and merge them into atomically monodisperse [Au_6_(PPh_3_)_6_]^2+^ clusters.

One of the most interesting and versatile clusters, Au_9_(PPh_3_)_8_(NO_3_)_3_, is still widely investigated despite first being prepared in the 1970s by reducing Au(PPh_3_)NO_3_ with NaBH_4_ in EtOH.^[^ ^11a^ ^]^ Interestingly, Simon and co‐workers recently obtained a tiny fraction of Au_14_(PPh_3_)_8_(NO_3_)_4_ clusters crystalized as fragile, light‐green platelets in a concentrated reaction mixture of Au_9_(PPh_3_)_8_(NO_3_)_3_ clusters.^[^ ^85^ ^]^ The skeletal symmetry can be approximated to *D_2h_*, similar to the Au_9_ cluster. However, unlike the Au_9_ cluster where NO_3_^−^ acts as a counter ion, in Au_14_ the Au(NO_3_) acts as a ligand with the short Au–O bond length (2.1 Å) fulfilling the configuration of the superatom model. Nonetheless, the low yield remains a challenging problem for further characterization of other properties. More recently, Shen et al. successfully isolated and crystallized metastable Au_9_(PPh_3_)_8_Cl via rapid extraction with a solvent of poor solubility (ether).^[^ ^52^ ^]^ The cluster is the first to display a body‐centered cubic (bcc) core framework where it is fixed by strong intermolecular van der Waals interactions between PPh_3_ ligands.

Interaction between Au and H atoms is of great interest because theoretical calculations and experimental evidence suggested that the H 1s electron mimics the 6s electron in an Au atom, i.e., a H atom is electronically equivalent to an Au atom. Hence, incorporation of a H atom in Au clusters does not change the geometric and electronic structure appreciably.^[^ ^120^ ^]^ Tsukuda and co‐workers synthesized several hydride‐adduct Au clusters by further addition of NaBH_4_ to the existing clusters.^[^ ^53^, ^56^, ^121^ ^]^ The inclusion of hydride from BH_4_^−^ was confirmed by isotope labeling with NaBD_4_. In contrast to the metastable [HAu_9_(PPh_3_)_8_]^2+^ cluster, mixed‐metal [HPdAu_10_(PPh_3_)_8_Cl_2_]^+^ and [HPdAu_8_M_2_(PPh_3_)_8_Cl_2_]^+^ (M = Ag, Cu) clusters are stable due to favorable affinity of H to Pd. The location of the H atom cannot be unambiguously established by XRD but it has been suggested that it migrates between the bridging and terminal sites close to the central Pd atom and Au surface atoms based on DFT calculations and NMR spectroscopy.^[^ ^56^, ^121^ ^]^

The stable and robust [H_3_Au_20_(PPh_3_)_12_](SbF_6_)_3_ cluster was recently synthesized by reduction of Au(PPh_3_)SbF_6_.^[^ ^20b^ ^]^ The structure consists of two Au_9_ and Au_11_ kernels fused through trigonal atoms from each unit. The stability is attributed to the electron‐withdrawing nature of the hydrides and the bridging hydrides at the joint edges resulting in short Au–Au distances. Similarly, the structure of [Au_22_H_3_(dppee)_7_]^3+^ prepared from Au_2_(dppee)Cl_2_ has been suggested to consist of two Au_11_ kernels formed by a triple bond (Au_11_≡Au_11_) with bridging hydrides.^[^ ^95^ ^]^ The largest hydride‐doped cluster reported to date, [Au_22_H_4_(dppo)_6_]^2+^, was realized in relatively high yield as an intermediate in the preparation of neutral Au_22_(dppo)_6_ which was formed by gradual loss of H in solution.^[^ ^96^ ^]^ Theoretical calculations revealed enhanced reactivity and catalytic activity of H‐doped Au clusters.^[^ ^120^, ^122^ ^]^ The hydrogen loss pathways in Au clusters open up the possibility of studying the mechanism of gold‐catalyzed hydrogen‐related reactions such as hydrogen evolution and hydrogenation reactions.

Heterometallic Au clusters have been shown to exhibit superior properties in terms of chemical/catalytic reactivity, structural stability, and modulation of electronic and optical properties. Synthesis of heterometallic clusters usually involves simultaneous co‐reduction of metal precursors or addition of foreign metal precursors to the existing clusters. Common precursors containing precious noble metals (Pt, Pd) are metal–phosphine complexes such as Pt(PPh_3_)_4_ and Pd(PPh_3_)_4_ (refer to Table 1). Tsukuda and co‐workers recently improved the yield of [PdAu_8_(PPh_3_)_8_]Cl_2_ to 80% with excellent purity by kinetic control of the reduction step in a modified synthesis starting with the Pd(PPh_3_)_4_ and Au(PPh_3_)Cl precursors;^[^ ^55^ ^]^ the previously reported yield was 58%.^[^ ^123^ ^]^ A similar yet simpler synthetic protocol affords a larger homolog, PdAu_10_(PPh_3_)_8_Cl_2_, which exhibits higher stability than Au_11_(PPh_3_)_8_Cl_2_ and near‐IR photoluminescence at 950 nm.^[^ ^64^ ^]^ A new mixed‐ligand [PdAu_24_(PPh_3_)_10_(SR)_5_Cl_2_]Cl cluster was recently obtained by addition of a thiolate (SR) ligand to the metal precursors.^[^ ^100^ ^]^ Such reported works highlight the rich chemistry of gold–phosphine where a range of clusters can be prepared by manipulating the conditions/reagents of similar preparative methods.

**Figure 2:**
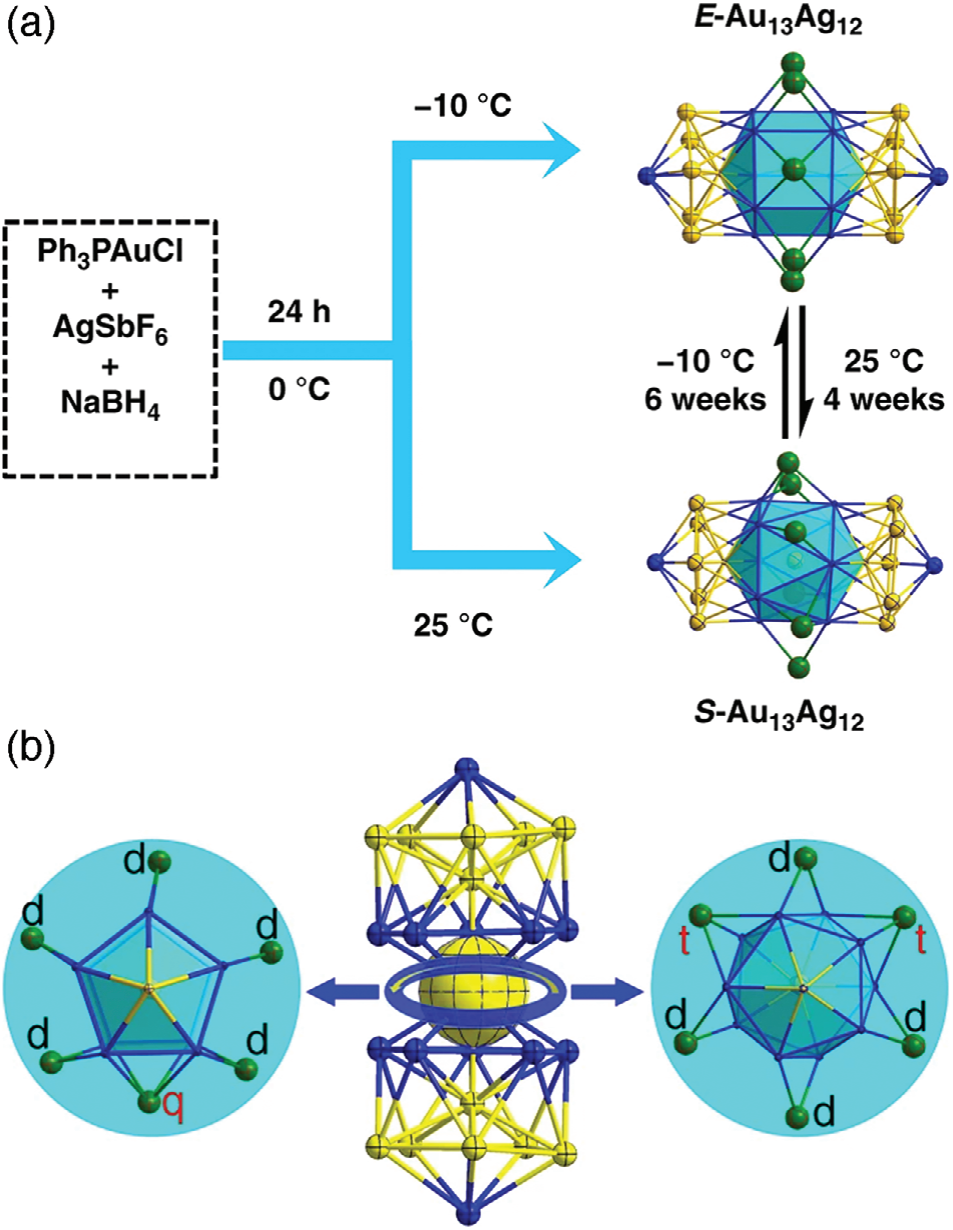
Schematic representation of a) synthesis of conformational isomers E‐ and S‐[Au_13_Ag_12_(PPh_3_)_10_Cl_8_]SbF_6_ clusters, and b) the corresponding top view of E‐(left) an S‐(right) isomers. Reproduced with permission under Creative Commons Attribution 4.0 International License.^[^ ^84^ ^]^ Copyright 2020, Nature Publishing Group.

The study of quantum size effects, phase transition, and structural transformation in bimetallic AuAg clusters is of particular interest, because these clusters have similar electronic configurations.^[^ ^31e^ ^]^ A new cluster, Au_8_Ag_3_(PPh_3_)_7_Cl_3_, was recently prepared by simultaneous reduction of Au(PPh_3_)Cl and AgSbF_6_ in dichloromethane (CH_2_Cl_2_).^[^ ^58^ ^]^ The three chlorine atoms are ligated to the Ag atoms due to favorable charge transfer in a trigonal C_3_ symmetry, and the cluster retains the incomplete icosahedral structure. Using a similar preparation with chloromethane (CH_3_Cl) as the solvent, Jin and co‐workers synthesized rod‐like [Au_13_Ag_12_(PPh_3_)_10_Cl_8_]SbF_6_ composed of two Au_7_Ag_6_ units fused at a shared Au vertex.^[^ ^84^ ^]^ Remarkably, the cluster selectively crystallizes into different conformational isomers depending on the crystallization temperature, with the E‐Au_13_Ag_12_ isomer produced at ‐10 °C and the S‐Au_13_Ag_12_ at 25 °C (**Figure** **2**). The rotational isomerization is completely reversible by manipulating the temperature. More recently, a new rod‐like Au_8_Ag_17_(PPh_3_)_10_Cl_10_ has been obtained by simultaneous reduction of AuCl_3_ and AgNO_3_ precursors.^[^ ^59^ ^]^

Mixed‐ligand clusters offer an opportunity to study metal–ligand interfacial chemistry and its impacts on the reactivity, stability, and electronic and optical properties. Importantly, preparation of high nuclearity clusters such as [Au_25_(PPh_3_)_10_(SR)_5_Cl_2_](SbF_6_)_2_ and [Au_37_(PPh_3_)_10_(SR)_10_Cl_2_]Cl is impossible to obtain by using phosphine ligands alone, unless assisted by thiolate ligands. It is reasonable to expect that the strong coordination ability of sulfur (S) would impart substantial influences on Au kernels. It has been shown that reduction of Au_2_(dppm)_2_Cl_2_ affords open icosahedral [Au_13_(dppm)_6_]Cl_5_, while in the presence of an S^2−^ precursor it produces the core+exo heart‐shaped [Au_8_(dppm)_4_S_2_]Cl_2_ cluster.^[^ ^81^ ^]^ The S^2−^ plays a dual role in the synthesis: a) to etch the Au surface atoms resulting in a smaller cluster and b) to coordinate with the exo Au atoms resulting in a heart‐shaped geometry. A surprising discovery by Jin et al. revealed that a facile synthesis of [Au_9_Ag_12_(dppm)_6_(SR)_4_X_6_]^3+^ (X = Cl/Br) by reducing HAuCl_4_ and AgNO_3_ in the presence of dppm and thiols (SR = SAdm, S‐^t^Bu) affords achiral and chiral isomers which can be separated by methanol and further enantiomeric separation by HPLC.^[^ ^124^ ^]^ These findings open a new window to the synthesis of novel clusters with unprecedented structures and properties.

Recently, inclusion of alkynyl ligands has expanded the catalog of stabilizing ligands for metal clusters. Unlike phosphines and thiolates, the bonding modes between alkynyl ligands and the metal kernel may involve *σ* and/or *π* bonding which gives stronger protection.^[^ ^125^ ^]^ Moreover, the *π*‐conjugated units impart strong perturbation to the electronic structures and optical properties.^[^ ^126^ ^]^ Successful preparations of a series of mixed phenylalkynyl‐phosphine costabilized Au clusters (Au_19_, Au_23_, Au_24_, Au_38_,Au_40_) have been demonstrated by reducing gold–phosphine complexes and AuC≡CPh.(refer to Table 1). The core nuclearity is controlled by the type of gold complexes and the ratio of gold precursors. Based on the synthetic parameters and observed trends, it is suggested that higher AuC≡CPh‐to‐Au(PPh_3_)^+^ ratios could produce higher nuclearity Au clusters.^[^ ^125^ ^]^ In addition, ligation with alkylnyl ligands leads to diverse staple motifs at the gold–ligand interface. For example, V‐shaped and L‐shaped motifs have been found in Au_19_ and Au_24_, respectively.

The use of multidentate phosphine ligands has expanded the variety of gold–phosphine clusters. Compared to PPh_3_, diphosphine ligands exhibit stronger binding due to their bidentate nature, i.e., multiple chelating sites, of the ligands. Owing to their bidentate nature, diphosphine‐ligated clusters are less labile and less fluxional than their PPh_3_‐ligated counterparts.^[^ ^127^ ^]^ Diphosphines have been shown to enable direct synthesis of size‐selective Au clusters and to tune the nuclearity by varying structural flexibility in a way that is otherwise impossible using monodentate phosphines (PPh_3_, PPh_2_Me, and PMe_2_Ph). In particular, different diphosphines were found to afford the same core size nuclearity and closed‐shell electronic structure with different core structures, e.g., [Au_11_(dppp)_5_]^3+^, [Au_11_(dppe)_6_]^3+^, [Au_11_(depp)_4_Cl_2_]^+^, which enables the study of structure–property relationships.^[^ ^74^, ^128^ ^]^

Of importance, Bertino et al. found that the Au core size can be tuned by changing the spacer length, (—CH_2_—)*_n_* in bis(diphenylphosphino)*_n_*–alkane ligands.^[^ ^129^ ^]^ The efficient suppression of broad size distribution by diphosphines *L^n^* (*L^n^* = 1,*n*‐bis(diphenylphosphino)*n*‐ane) highlights the importance of steric and chelating effects on the selective formation of gold–phosphine clusters.^[^ ^117b^ ^]^ A small spacer, e.g.*, n* = 3 in diphosphine has less flexibility and efficiency at binding to multiple Au sites and is prone to produce monodisperse Au clusters in high abundance.^[^ ^27^, ^130^ ^]^ Additionally, the ratio of [L]/[PPh_3_] was found to efficiently tune the selectivity of core size nuclearity. Pettibone and Hudgens obtained nearly monodisperse octagold ([Au_8_L^6^_4_]^2+^), nonagold ([Au_9_L^6^_4_Cl]^2+^) and decagold ([Au_10_L^6^_4_]^2+^ and Au_10_L^6^_5_]^2+^) when the ligand ratio was [L^6^]/[PPh_3_] = 4, 0.4 and 8, respectively.^[^ ^27a^ ^]^

Furthermore, bidentate phosphine ligands may sometimes lead to unique geometry that is not characterized by the conventional closed polyhedra and display remarkably different electronic and optical properties.^[^ ^74^, ^131^ ^]^ The generic Au core structure of this type is described by [core+*exo*]‐type Au clusters. The first example is pentagold Au_5_(dppm^−^)_3_(dppm)(NO_3_)_2_ that exhibits an extra Au atom coordinated to the corner of an edge of a tetrahedral core.^[^ ^6g^ ^]^ This unique Au atom is also bonded to a carbon atom becoming the first Au cluster to exhibit an Au—C bond. Konishi and co‐workers have successfully prepared and structurally characterized several Au clusters (Au_6_–Au_8_, Au_11_) with core‐exo geometry using 1,3‐bis(diphenylphosphino)propane (dppp) and 1,2‐bis(diphenylphosphino)ethane (dppe) ligands.^[^ ^132^ ^]^ The authors established a generic structural description of the Au core as [Au_core_+Au_exo_] where the Au_core_ is polyhedral and identified [Au_4_+2Au], [Au_6_+Au], [Au_6_+2Au] and [Au_9_+2Au] for [Au_6_(dppp)_4_]^2+^, [Au_7_(dppp)_4_]^3+^, [Au_8_(dppp)_4_Cl_2_]^2+^ and [Au_11_(dppe)_6_]^3+^ clusters.^[^ ^49^, ^133^ ^]^

In addition, it is possible to produce high nuclearity Au clusters using multidentate phosphines. Previously, there was a gap in gold–phosphine clusters from Au_14_ to Au_38_ (inclusively) using monodentate phosphines (PPh_3_, PPh_2_Me, PMe_2_Ph).^[^ ^38^, ^109^, ^134^ ^]^ New, high nuclearity Au clusters produced via reduction of the corresponding Au precursors by NaBH_4_ such as Au_18_(dppm)_6_Cl_4_, Au_20_(dppe), Au_20_(PP_3_)_4_Cl_4_ and Au_22_(dppo)_6_ have been recently reported. Some new properties have been identified from these clusters, such as high charge states (Au_18_), large number of uncoordinated sites (Au_22_) and polymorphism (Au_22_). The stability of these clusters is explained by the elongated geometrical structure, rather than the closed‐shell electronic structure based on the jellium model.

Intercluster conversion has been a successful strategy to prepare size‐selective Au clusters that are otherwise inaccessible with direct synthesis from the gold salt or complex precursors. Classic examples include preparation of Au_4_(µ‐I)_2_(PPh_3_)_4_, Au_6_(dppp)_4_(NO_3_)_2_ and Au_8_(PPh_3_)_8_(NO_3_)_2_ clusters from the parent cluster Au_9_(PPh_3_)_8_(NO_3_)_3_ reported in the 1980s (refer to Table 1).^[^ ^6^, ^11^ ^]^ The synthesis of Au_8_(PPh_3_)_8_(NO_3_)_2_ is performed exclusively by reacting Au_9_(PPh_3_)_8_(NO_3_)_3_ with excess PPh_3_. Intercluster conversion of thiolate‐protected Au clusters into phosphine‐ligated versions under the action of PPh_3_ led to the surprising realization of the ability of PPh_3_ to etch the surface of Au clusters protected by stronger Au–S bonding. More recently, Wu and co‐workers demonstrated the etching ability of PPh_3_ to successfully convert a series of thiolate‐protected Au clusters, including 3 nm particles, into PPh_3_‐ligated Au_11_ intermediates in reverse ligand exchange.^[^ ^135^ ^]^ However, no intercluster conversion was observed for other ligand‐protected (PVP, citrate) Au clusters, thus indicating the unique action of PPh_3_ on Au—S bonds.^[^ ^135b^ ^]^

**Figure 3:**
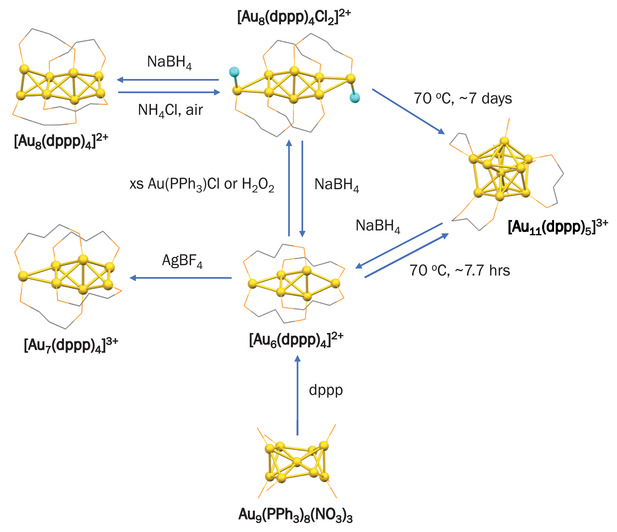
Reaction pathways of intercluster conversion of several diphosphine‐ligated Au clusters.

Reversible conversion offers a versatile route to rational synthetic design by manipulating the synthetic conditions. Early attempts showed such reversibility was possible when PPh_3_‐ligated nonagold was transformed into octagold by adding suitable reagents.^[^ ^115a^ ^]^ A reversible isomerization of [Au_8_(dppp)_4_Cl_2_](NO_3_)_2_ and [Au_8_(dppp)_4_](NO_3_)_2_ was achieved under redox‐mediated conditions. [Au_8_(dppp)_4_Cl_2_]^2+^ yields [Au_8_(dppp)_4_]^2+^ by NaBH_4_ reduction and the reverse reaction proceeds aerobically when adding NH_4_Cl.^[^ ^51^, ^136^ ^]^ Both novel octagold clusters contain edge‐shared tetrahedron Au motifs owing to the chelating ability of the dppp ligand to bind to multiple Au sites which determines the geometry. Under oxidative/reductive conditions, Huang and co‐workers demonstrated a reversible intercluster conversion among [Au_6_(dppp)_4_]^2+^, [Au_8_(dppp)_4_Cl_2_]^2+^, and [Au_11_(dppp)_5_]^3+^ clusters.^[^ ^73^, ^137^ ^]^ In general, NaBH_4_ reduction affords smaller and lower‐charge state clusters, while heating at 70°C reverses the process to yield the electronically stable [Au_11_(dppp)_5_]^3+^ cluster. The reaction pathways in intercluster conversion of diphosphine‐ligated Au clusters are illustrated in **Figure** **3**.

### 2.2 Etching Step

The success of preparing numerous size‐selective gold–phosphine clusters demonstrates the richness of gold–phosphine chemistry, but also poses an intriguing question: if we can produce so many varying size‐selective Au clusters, what factors determine the selectivity and stability of their formation? Using DFT calculations to investigate the growth mechanism, Guidez et al. noted that the complexity in predicting the formation of Au clusters is attributed to the poor selectivity in the product formation due to the reaction being exothermic.^[^ ^138^ ^]^ Scalar relativistic DFT calculations by Hong et al. concluded that selectivity in Au cluster syntheses is more complex than the stability factor alone.^[^ ^130^ ^]^ Numerous efforts to investigate the mechanism of the synthesis, roles of ligands, and reaction conditions have led to key findings about the growth and formation of gold–phosphine clusters and thus advanced the knowledge of gold cluster chemistry for rational synthesis design.

Fast reduction of Au precursors by relatively strong reducing agents such as NaBH_4_ and LiBH_4_ (cf. ascorbic acid, trisodium citrate) yields large Au clusters with varying cluster sizes and therefore postsynthetic purification is often necessary to isolate specific products. In contrast, weak reducing agents can yield monodisperse Au particles due to the slow reduction, nucleation, and growth which influence the size. Kinetic control greatly influences reduction of Au precursors, with parameters such as reducing agent, solvent, temperature, and concentration governing the reduction step. In the case of borohydride reduction, the kinetics of reaction is influenced by the solvent or the solvent mixture, which consequently affects the reaction rate of NaBH_4_ decomposition which liberates H_2_ gases as a reducing agent.^[^ ^139^ ^]^ Another viable way to tune the reducing ability is to add NaOH to retard the hydrolysis of NaBH_4_.^[^ ^140^ ^]^ Weak reducing agents such as borane‐*tert*‐butylamine (BTBA) and 9‐borabicyclo[3.3.1]nonane (9‐BBN) have been employed to produce narrow size distributions of phosphine‐stabilized Au clusters.^[^ ^26^, ^117^ ^]^

A critical question arises as to why monodisperse, specific size‐selective Au clusters are formed in a fast reduction environment since such conditions generally lead to large, polydisperse clusters of varying sizes.^[^ ^117b^ ^]^ It seems that an unprecedented etching step is most likely to happen at some stage during cluster synthesis. An early work by Duan and Nie reported that polyethylenimine (PEI) could etch gold nanoparticles to form fluorescent Au_8_ clusters, however the mechanism was not elucidated.^[^ ^141^ ^]^ For phosphine‐ligated Au clusters, remarkable findings by Pettibone and Hudgens revealed that competing cycles of growth and etching around stable clusters are responsible for formation of nearly monodisperse Au clusters, and that the PPh_3_ ligand serves a dual role as a stabilizing and etching agent during synthesis. This cyclic growth and etching process was unknown previously.^[^ ^27^, ^142^ ^]^ The etching role of PPh_3_ explains the formation of [Au_8_(PPh_3_)_7_]^2+^ from [Au_9_(PPh_3_)_8_]^3+^ because adding PPh_3_ leads to removal of a [Au(PPh_3_)]^+^ fragment.^[^ ^11b^ ^]^ A similar size convergence from polydisperse AuPd alloy clusters into PdAu_8_(PPh_3_)_8_Cl_2_ was observed by Tsukuda and co‐workers.^[^ ^55^ ^]^

**Figure 4:**
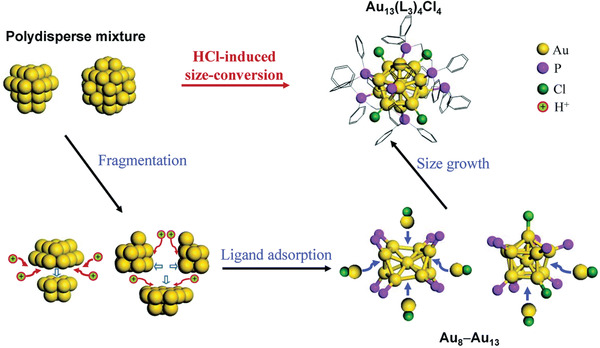
Schematic illustration of HCl‐induced Au_13_ synthesis involving three steps: fragmentation of larger initial Au clusters into intermediates, ligand adsorption stabilizing the intermediates and size growth of the intermediates into monodisperse Au_13_. Reproduced with permission.^[^ ^144a^ ^]^ Copyright 2015, Royal Society of Chemistry.

Size convergence into monodisperse [Au_13_(dppe)_5_Cl_2_]Cl_3_ was achieved by etching a polydisperse mixture of Au clusters with HCl.^[^ ^80^ ^]^ It is suggested that the cooperative action of H_3_O^+^ and Cl^−^ (from HCl) weakens the coordination of phosphine ligands to Au atoms, leading to formation of thermodynamically stable tridecagold whose stability originates from the closed electronic and geometric shell.^[^ ^143^ ^]^ Detailed studies using a combination of in situ X‐ray/UV–visible absorption spectroscopy and time‐dependent mass spectrometry revealed that the HCl‐induced synthesis of Au_13_ involves three key steps: i) fragmentation of nascent, larger and polydisperse (Au_15_–Au_65_) clusters into metastable intermediates (Au_8_–Au_13_) ii) rapid stabilization of these intermediates by ligands (phosphines and chloride), followed by iii) the growth of these intermediates into monodisperse Au_13_ clusters by incorporating the existing Au(I)–Cl complexes (**Figure** **4**).^[^ ^144^ ^]^

More recently, Huang et al. discovered that ammonia (NH_3_) could act as an etching agent to convert a polydisperse PPh_3_‐ligated Au*_x_* (*x* = 6–11) cluster mixture into monodisperse [Au_6_(PPh_3_)_6_]^2+^.^[^ ^46^ ^]^ NH_3_ acts to deplete [Au(PPh_3_)]^+^ fragments by forming a stable complex [NH_4_AuPPh_3_Cl]^+^. The formation of hexagold clusters proceeds via a two‐step process. Removal of [Au(PPh_3_)]^+^ causes structural instability and the cluster reorganizes itself to regain stability. Shorter Au—Au bonds in [Au_6_(PPh_3_)_6_]^2+^ compared to other larger clusters (Au*_x_*, *x* > 6) indicate the cluster's stability over others against etching and therefore [Au_6_(PPh_3_)_6_]^2+^ is the major product in the synthesis.

Thiols are reported to be an effective etching agent to convert polydisperse Au*_n_* (PPh_3_)*_m_* X*_y_* (X = halide) clusters into stable size‐specific clusters. It has been found that the type of thiol ligands and synthetic conditions determine the structure and composition of the final clusters.^[^ ^145^ ^]^ According to Lin et al., etching PPh_3_‐stabilized Au nanoparticles with alkyl thiols produces bi‐icosahedral [Au_25_(PPh_3_)_10_(SR)_5_X_2_]^2+^ (X = Cl/Br) nanorods while aromatic thiols yield Au_25_(SR)_18_ nanospheres due to the differences in steric and electronic properties of thiols.^[^ ^146^ ^]^ A proposed mechanism involves detachment of Au_3_(PPh_3_)_2_(SR)X fragments, as observed in the ESI mass spectra, upon etching by thiolate and size focusing into thermodynamically stable Au_25_ clusters.^[^ ^147^ ^]^ In contrast, the gold(I) complex Au_2_L^5^(SR) was found to be the thiol‐etched product of diphosphine‐ligated Au clusters.^[^ ^148^ ^]^ These findings not only give insights into etching processes and the reactivity of clusters towards different thiols but also extend the catalog of cluster syntheses.

### 2.3 Ligand Exchange

Ligands have multitudinous roles in cluster chemistry. First, they modulate the electronic structure and/or provide electronic stabilization by filling the superatomic electron shells.^[^ ^149^ ^]^ Second, they contribute to the stability of clusters by steric stabilization and prevent aggregation in both solution and solid state. Third, they regulate the solubility and dispersion of clusters in specific solvents. An appropriate choice of ligands to passivate the metal core permits tuning the solubility of clusters or nanoparticles in organic and aqueous media.^[^ ^150^ ^]^ Fourth, they introduce functionalities for specific purposes such as binding to biomolecules and heavy metals for sensing applications.^[^ ^151^ ^]^ Fifth, ligands play a critical role in defining optical properties such as absorption and photoluminescence.^[^ ^152^ ^]^ Chirality in Au clusters can also be achieved by introducing chiral ligands.^[^ ^153^ ^]^ Finally, they allow development of new synthetic strategies for preparing otherwise inaccessible clusters by direct chemical reduction.^[^ ^14^ ^]^ The nature of stabilizing ligands determines the chemical reactivity, solubility, ability to bind to analytes and self‐assembly.^[^ ^154^ ^]^ Bonding between metals and ligands defines the reactivity of clusters in dissociation, fragmentation, and addition reactions.

Complexity in cluster synthesis, control over the core size and dependency on ligands becomes a major issue to passivate specific ligands at the surface of Au clusters during synthesis. Thus, ligand exchange is a viable and convenient approach to incorporate functionality and introduce new synthetic strategies to produce clusters with tailored properties for applications such as chemical sensing of metal ions, anions, and biological molecules, and for fluorescent labeling and imaging. Ligand exchange on gold–phosphine clusters leads to three scenarios: 1) the Au core remains unchanged and total substitution of the ligands occurs, 2) the Au cluster converts into other Au clusters, and 3) the Au cluster grows into a larger nanoparticle. According to the thermodynamic analysis from the Gibbs‐Thomson equation, the final size of Au clusters after ligand exchange depends on the strength of ligand–gold interactions.^[^ ^155^ ^]^ A weaker ligand–gold interaction results in larger particles because of the reduced surface energy of larger particles.

Gold–phosphine clusters have the advantage of facile ligand exchange/removal owing to weaker Au–P bonding.^[^ ^156^ ^]^ These clusters are usually soluble in dichloromethane (DCM), methanol (MeOH), or ethanol (EtOH). To achieve solubility in polar (e.g., water) or nonpolar (e.g., hexane) solvents, ligand exchange must be performed. For biology‐related applications, which are necessarily performed in aqueous solution, phosphine ligands are usually exchanged with ligands containing water‐soluble functional groups_._^[^ ^150b^ ^]^ Typically, clusters exchanged with glutathione and carboxylic acid functionalized thiols are soluble and stable in water. Early evidence of a complete ligand exchange was observed in Au_4_(PPh_3_)_4_(µ‐I)_2_ by exchanging PPh_3_ with bis(diphenylphosphino)methane (dppm) in tetrahydrofuran (THF), resulting in a slightly distorted tetrahedral structure of Au_4_(dppm)_3_I_2_ due to the strain effect imparted by dppm.^[^ ^157^ ^]^

Several factors that influence the relative strength of Au–P interactions are steric hindrance, cluster size, charge state, charge donating ability, and proton affinity of phosphines. While these competing factors complicate the understanding of ligated Au clusters, it is feasible to determine the dominant influence. Detailed investigations using a series of substituted monophosphines (PMe_3_, PPhMe_2_, PPh_2_Me, PCy_3_, PPhCy_2_, PPh_2_Cy) in ligand exchange with PPh_3_‐ligated Au clusters revealed the relative Au–P bond strength.^[^ ^26b^ ^]^ The relative ligand binding energy has been established to increase in the order: PMe_3_ < PPhMe_2_ < PPh_2_Me < PPh_3_ < PPh_2_Cy < PPhCy_2_ < PCy_3_. By comparing these findings and using theoretical calculations of Au–P binding energies^[^ ^158^ ^]^ and proton affinities of the ligands,^[^ ^159^ ^]^ the primary effect in driving the ligand exchange process has been ascribed to the electron‐donating ability of the substituted monophosphines.

Steric effects play a substantial role in the kinetics of ligand exchange and in dictating the final composition and size. Systematic studies on different positions of the methyl group in tri(tolyl)phosphine (ortho‐, metha‐, para‐) ligands by Parrish et al. showed that tri(m‐tolyl)phosphine and tri(p‐tolyl)phosphine exchanged effectively with PPh_3_ ligands in Au_8_(PPh_3_)_7_^2+^ whereas tri(o‐tolyl)phosphine remained unreactive.^[^ ^160^ ^]^ DFT calculations revealed that tri(o‐tolyl)phosphine has the largest steric interaction between the ligands and the gold core, rendering the ligand exchange of PPh_3_ least favorable among the tri(tolyl)phosphines.

Additionally, core size nuclearity and bound ligands influence the final size, stability, and structure of the clusters resulting from the exchange.^[^ ^161^ ^]^ Undecagold clusters are an ideal model studying the mechanism and dynamics of ligand exchange, owing to the stable electronic configuration and the molecular‐like electronic structure. Through a systematic and comprehensive study, Hutchison and co‐workers revealed that Au_11_(PPh_3_)_8_Cl_3_ retained a similar core size, whereas Au_11_(PPh_3_)_7_Cl_3_ (or a mixture of both isostructures) afforded Au_25_ clusters after ligand exchange with glutathione, thus highlighting remarkably different reactivities of these clusters toward thiols.^[^ ^67^ ^]^ Another undecagold homologue, Au_11_(PPh_3_)_7_Br_3_, on an Al_2_O_3_ surface exhibits different results depending on the incoming ligands. It retains its core size with L‐glutathione (GSH), and leaches and decomposes into smaller clusters when exposed to phenylethanethiol (2‐PET).^[^ ^162^ ^]^ Interestingly, ligand exchange with alkanethiols in Au_11_(PPh_3_)_7_Cl_3_ promotes surface trap energy states that induce photoluminescence in the visible region.^[^ ^119^ ^]^

The mechanism of ligand exchange provides invaluable insights for understanding the cluster reactivity and bonding between ligands and the surface of Au clusters. In Au_11_(PPh_3_)_8_Cl_3_, bound PPh_3_ is liberated as free PPh_3,_ and the core remains intact, followed by subsequent adsorption of thiols.^[^ ^163^ ^]^ However, the larger counterparts, Au_55_(PPh_3_)_14_Cl_6_ and Au_101_(PPh_3_)_21_Cl_5,_ show a different process involving a three‐stage mechanism. Using ^31^P spectroscopy and trapping experiments, Hutchison and co‐workers identified a three‐stage mechanism: i) Au(PPh_3_)Cl is fragmented from the cluster surface, ii) assisted direct removal of the PPh_3_ by Au(PPh_3_)Cl in the form of polyphosphine Au complexes AuCl(PPh_3_)_n_, and finally iii) adsorption and reorganization of thiols into a more crystalline state.^[^ ^164^ ^]^ In the final stage, the rearrangement of thiols to form chemically adsorbed thiolate (Au‐SR) and Au atoms in the form of Au(PPh_3_)Cl may explain a longer reaction time in ligand exchange.

Reverse ligand exchange with PPh_3_ was demonstrated by Wang and Murray using phenylethanethiolate‐protected Au_38_(SC2Ph)_24_ clusters.^[^ ^165^ ^]^ Surprisingly, the exchange of thiols with phosphines is rapid, and the Au(I)SC2Ph complex is released from the gold core instead of phenylethanethiolate (SC2Ph), disulfide, or anionic species. Such a counterintuitive finding was thought unlikely because Au–S bonding is stronger than Au–P. Importantly, PPh_3_ has been shown to replace some thiols in the core–shell Au_25_(SR)_18_^−^ and structurally convert it into the bi‐icosahedral [Au_25_(PPh_3_)_10_(SR)_5_Cl_2_]^2+^ cluster.^[^ ^135a^ ^]^ Notably, not all thiols are replaced by PPh_3_ ligands. The interaction energy between ligands and the Au core alone is not enough to explain the dynamics and degree of ligand exchange. Additionally, the ratio of PPh:SR is crucial in determining the outcome. A small ratio does not lead toward ligand exchange and/or intercluster conversion, and an excessively large ratio leads to cluster decomposition.^[^ ^135a^ ^]^

## 3 Properties of Phosphine Ligated Au Clusters

### 3.1 Electronic Structures

The electronic structures of Au clusters are known to be completely different from that of bulk gold. When the particle size approaches the Fermi wavelength of an electron, the chemical, electronic and optical properties of these small clusters deviate significantly from bulk gold due to the particle's finite of dimension. As the size reduces, the metallic state characterized by a continuous band disappears and discrete energy levels emerge indicating a nonmetallic state. As early as 1962, Kubo presented an examination on the electronic properties of fine metallic particles using the one‐electron approximation to metallic electrons.^[^ ^166^ ^]^ Furthermore, the detailed analysis revealed that the energy levels of metallic particles with finite size are quantized, and such quantum size effects also dominate the thermodynamics and magnetic properties such as heat capacity and spin paramagnetism.

The quantization of energy levels becomes distinct when the energy spacing is much larger than the thermal energy (25.7 meV) at room temperature. The average energy level spacing, *δ* is given by^[^ ^167^ ^]^

(1) $$\delta \approx \frac{E_{F}}{N}$$

where *E*_F_ is the Fermi energy (5.52 eV for Au) and *N* is the total number of valence electrons in the particle.^[^ ^1^ ^]^ In the case of Au, *N* is 215. Since one Au atom has one valence electron, this is equivalent to 215 Au atoms, or a critical size of around 2 nm.^[^ ^30a^ ^]^ Although this model neglects the effects of ligand stabilization and electron–electron interaction, it nonetheless gives a qualitative insight on the size‐dependent energy level spacing. Studies by Jin and co‐workers using thiolate‐protected Au clusters revealed that the metallic‐to‐nonmetallic transition occurs around 2.2 nm (the Au_246_ cluster).^[^ ^168^ ^]^ This critical size is important to distinguish between metallic and nonmetallic behavior and allows the study of the structural transition from face‐centered cubic (fcc) to non‐fcc and other related phenomena.^[^ ^169^ ^]^

Theoretical studies and large‐scale simulations have been repeatedly utilized to understand electronic stabilities of gold clusters. Häkkinen reviewed the origin of shell structures in confined quantum systems and summarized the key points to solve the electron–electron interactions in the electron gas model.^[^ ^170^ ^]^ To simplify the Hamiltonian, the ionic core structure can be neglected when there are weak ion core–valence electron interactions and electrons are confined in a potential well which forms delocalized orbitals surrounding the positively charged ionic core. Complete exclusion of the detailed ionic structure is justified by a combined effect of screening by inner core electrons and the Pauli exclusion principle, and experimentally observed properties.^[^ ^171^ ^]^ This approximation of electron gas in a medium of averaged ion density is termed the jellium model. Nowadays, the implementation of the jellium model in current DFT code packages such as GPAW^[^ ^172^ ^]^ paved the way to solving the Kohn‐Sham equations for any shape of the fixed jellium density. The symmetry analyses of the KS wavefunctions are performed by decomposing *ψ_i_(r)* into their respective angular momenta. The major limitation of the jellium model is the approximated ionic structure. There are two requirements that must be met for this model to be applied in cluster studies according to Brack:^[^ ^171^ ^]^ a) strong delocalization of the valence electrons, and b) the ionic background must be responsive to any perturbations. Following these requirements, the jellium model is mainly applicable to alkali, alkali‐earth, and noble metals where the valence electrons are contained in the outermost s‐orbital.

The stability of metal clusters has been explained using a “noble‐gas superatom” analogy in which the valence electrons of the cluster atoms can delocalize to the ligands to achieve the noble‐gas electronic configuration, i.e., shell‐closing of the superatomic orbitals.^[^ ^173^ ^]^ The jellium model is used to explain the stability of magic number of metal atoms in clusters.^[^ ^174^ ^]^ In this model, metal clusters are considered to be positively charged spheres with electronic shells filled by the valence electrons provided by the constituent individual metal atoms.^[^ ^175^ ^]^ It has successfully explained the stability of magic numbers of sodium clusters, Na*_n_* (*n* = 8, 20, 40, and 58)^[^ ^174^ ^]^ and Al_13_.^[^ ^176^ ^]^ The electrons fill the orbitals in an analogous way to atoms. However, the ordering of the energy levels is different due to the fact that charge is distributed differently in atoms compared with the jellium model of clusters.^[^ ^176^, ^177^ ^]^ The analogous Aufbau principle for metal clusters is 1S^2^ | 1P^6^ | 1D^10^ | 2S^2^ 1F^14^ | 2P^6^ 1G^18^ |… corresponding to 2, 8, 18, 34, 58,… valence electrons in closed electronic supershells,^[^ ^173^, ^177^ ^]^ although stability has also been shown for 20‐ and 40‐electron clusters, depending on mean‐field potentials.^[^ ^175^ ^]^ The observed exceptional electronic stability in metal clusters with total electron count as above is due to the corresponding electron shell closures, as well as high ionization threshold.

**Figure 5:**
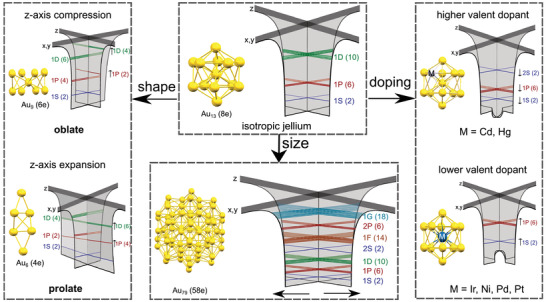
The effect of size, shape, and doping on the isotropic jellium potentials proposed by Tsukuda and co‐workers.^[^ ^31^, ^178^ ^]^

This description works for bare metal clusters, but does not take into account contribution from protecting ligands. Walter et al. put forth a unifying superatomic concept (SAC) to describe the stability of ligated Au clusters based on the closed electronic shell of noble‐gas superatoms.^[^ ^173a^ ^]^ The closed‐shell electron counts, n* can be described by

(2) $$n^{*}=N_{\mathrm{Au}}-N_{L}-N_{L^{\prime }}-q$$

where *N*_Au_ is the number of valence electrons from Au atoms, *N*_L_ and *N*_L_ *_’_* are the number of Au valence electrons withdrawn by ligands L and L’, and *q* is the net charge on the Au cluster. Using this equation, Au_11_(PPh_3_)_7_Cl_3_, [Au_11_(dppp)_5_]^3+^ and [Au_13_(dppe)_5_Cl_2_]^3+^ clusters have 8 delocalized electrons (8e), as phosphine ligands are not electron withdrawing, but halide ligands are. An extensive list of the then known ligated Au clusters and their respective free‐electron count was reported by Häkkinen in 2015.^[^ ^170^ ^]^ Moreover, the description of the electronic structure of gold and mixed‐gold clusters based on the Jellium model (see **Figure** **5**) has been discussed extensively by Tsukuda and co‐workers recently.^[^ ^31^, ^178^ ^]^

Inevitably, not all experimentally synthesized Au clusters exhibit magic numbers of free valence electrons described by the superatom concept. In 2015, Mingos published a perspective on the structural and bond patterns of the metal core in Au clusters.^[^ ^179^ ^]^ Prolate or oblate distortions of some gold clusters with nonspherical geometries, cause the removal of the degeneracy of the P subshells due to a Jahn‐Teller effect, as shown in Figure 5. The triply degenerate 1P orbitals in the spherical geometries are split into two: 1P_z_ is destabilized compared to 1P_x_ and 1P_y_ for oblate clusters and vice versa for prolate clusters. Known prolate Au clusters with 1S^2^ 1P^2^ closed subshell electronic structures are [Au_6_(dppp)_4_]^2+^ and [Au_6_(PPh_3_)_6_]^2+^.^[^ ^6^, ^180^ ^]^ On the other hand, oblate gold phosphine clusters with 1S^2^ 1P^4^ closed subshell electronic structures are [Au_7_(PPh_3_)_7_]^+^, [Au_8_(PPh_3_)_7_]^2+^ and [Au_9_(PPh_3_)_8_]^3+^.^[^ ^6^, ^181^ ^]^

Most of the reported ligand‐stabilized Au clusters have geometries based on distinct icosahedral and cuboctahedral core building blocks. This can be explained using the united cluster approach for Au clusters, which is an extension to Mulliken's united atom model.^[^ ^182^ ^]^ An isolated gold core structure with either icosahedral or cuboctahedral symmetry can be described as spherical, that is, its skeletal molecular orbitals (MOs) have the same nodal properties as the atomic orbitals of Au. Within this theorem, the MOs of the condensed polyspherical clusters of gold atoms may be described as linear combinations of the single cluster MOs. This has been observed in the cases of Au_25_ and Au_37_, with Au_13_ as its building block.^[^ ^183^ ^]^ Yang and co‐workers reported that the superatomic bonding of 2 × Au_11_ (8e) provides stability to [Au_20_(PPhpy_2_)_10_Cl_4_]^2+^ with 14e valence electrons,^[^ ^184^ ^]^ which does not satisfy the superatom model but can be classified under the united cluster model. The electronic structure description of the [Au_14_(PPh_3_)_8_(NO_3_)_4_] cluster showed a partially filled d shell (10e) based on SAC, whereas the united cluster model describes it as a pair of bicapped trigonal bipyramids linked by the strong inner Au–Au bond.^[^ ^85^ ^]^ Cheng et al. proposed the super valence bond (SVB) model, which is seemingly analogous to the united cluster approach.^[^ ^185^ ^]^ Subsequently, they developed the superatom network (SAN) model to deduce the high stability of ligated Au clusters with low symmetries.^[^ ^186^ ^]^ Recently, a grand unified model (GUM) was introduced to provide a universal description of the stabilities for all ligand‐stabilized Au clusters.^[^ ^187^ ^]^ Here, the structural evolution of the gold core is not expressed as the addition of Au atoms. Instead, the cores of the ligated gold clusters are formed from triangular Au_3_ (2e), and tetrahedral Au_4_ (2e), elementary blocks that satisfy the duet rule of the valence shell. Expanding the proposed elementary blocks, Muñoz‐Castro described the 18‐electron Au_22_^4+^ core of the Au_70_S_20_(PPh_3_)_12_ cluster as a tetrahedral aggregation of Au_6_ units.^[^ ^188^ ^]^

Modifying the electronic structure of the Au clusters can be achieved by doping with different metal atoms. The effect of doping with lower valent atoms, such as Pt, Pd, and Ni, at the central position of the icosahedral core of Au_13_ results in the reduction of the effective potential, which then causes destabilization of the superatomic orbitals (SOs).^[^ ^76^, ^189^ ^]^ On the other hand, the more attractive effective potential at the center of the icosahedral core stabilizes these orbitals when higher valent atoms are used instead. This in turn changes the ordering of filling of the SOs to 1S^2^ | 1P^6^ | 2S^2^ | 1D^10^ | 2P^6^ | 1F^14^ |… corresponding to 2, 8, 10, 20, 26, 40, … valence electrons.^[^ ^190^ ^]^ Metal atoms such as Pd, Pt, Ni, and Ir have shown central positioning in the Au_13_ core,^[^ ^191^ ^]^ however, the location of the dopant is not always reported to be at the central position. Higher valent atoms such as Hg and Cd prefer the surface sites,^[^ ^191^ ^]^ which does not follow the simplified qualitative jellium model in Figure 5. Yet, stabilization of the orbitals was observed using differential pulse voltammetry for Cd‐ and Hg‐doped inner Au_13_ cores of the Au_25_ clusters.^[^ ^191^, ^192^ ^]^ Omoda et al. proposed that this could be attributed to the deformation of the 1S and 1P orbitals, forming an electronically dipolar superatom.^[^ ^190^ ^]^ Additionally, doping by hydrogen atoms produced no changes in the adiabatic electron affinity between Au*_n_*_+1_ and HAu*_n_*. Tsukuda and co‐workers demonstrated that the adsorption of hydride (H^−^) onto both PPh_3_‐ligated Au_9_ and PdAu_8_ clusters generated spherical metallic cores of the otherwise oblate‐shaped Au_9_ core.^[^ ^53^, ^56^ ^]^ Cirri et al. reported that the halides, Cl^−^ and Br^−^, showed similar electronic effects as hydrides in perturbing the electronic structure of [Au_9_(PPh_3_)_8_]^3+^.^[^ ^193^ ^]^

Understanding of the electronic structure of Au clusters comes from both computational and experimental spectroscopic methods. DFT already provides an effective description of the densities of states for these Au clusters. Additionally, the relativistic effects in the electronic levels and molecular orbitals of Au clusters have been investigated using the four‐component methodology of the Dirac equation. Muñoz‐Castro and Arratia‐Perez demonstrated the variation and splitting of energy levels when spin–orbit corrections (SOC) are employed.^[^ ^194^ ^]^ Interestingly, the spin–orbit term lowers the isomerization barrier for the D_2h_ ↔ O_h_ core rearrangement in the Au_6_ cluster.^[^ ^195^ ^]^

### 3.2 Optical (Absorption and Photoluminescence) Properties

The optical properties of Au clusters and nanoparticles are remarkably different. While Au nanoparticles display a localized surface plasmon resonance (LSPR), Au clusters exhibit molecular‐like, distinct peaks because of the discrete energy levels arising from their quantum size effects.^[^ ^196^ ^]^ As a consequence, Au clusters feature excitonic characteristics with long‐lived excited states and power‐independent electron dynamics.^[^ ^197^ ^]^ The transition from the nanoparticle to the cluster regime has been shown experimentally to occur at around 2.2 nm for gold, corresponding to 246 Au atoms.^[^ ^168^ ^]^ Importantly, absorption peaks in the visible spectrum are often ascribed to metal core electronic transitions.

In general, the optical properties of gold–phosphine clusters are profoundly defined by the core geometry, size nuclearity, and charge state, but less sensitive to changes in phosphine ligands and counter anions (halide, nitrate etc.).^[^ ^134^, ^193^ ^]^ For example, PPh_3_‐ligated PdAu_8_ with Cl^−^ and NO_3_^−^ anions have similar absorption peaks (entries 5 and 6 in **Table** **2**). Theoretical calculations also confirmed that different anions (halide, bromide, and chloride) show similar electronic structures, and thus optical spectra, for [Au_9_(PPh_3_)_8_]^3+^.^[^ ^193^ ^]^ More recently, Cirri et al. demonstrated similarities in the gas‐phase and solution‐phase spectra of [Au_9_(PPh_3_)_8_]^3+^.^[^ ^198^ ^]^ Such unique features provide a fingerprint for gold–phosphine clusters and allow identification using absorption spectroscopy.^[^ ^199^ ^]^ Table 2 summarizes the absorption peaks of several gold–phosphine clusters together with their HOMO–LUMO optical gap.

**Table 2:** Summary of absorption peaks in UV–visible spectrum for gold–phosphine clusters

Gold–phosphine clusters	Absorbance [nm]	HOMO–LUMO gap [eV]	PL [nm]	Refs.
Au_5_Cu_6_(dppf)_2_(SR)_6_BPh_4_	379, 492, 617	1.73		[45]
Au_6_(PPh_3_)_6_(NO_3_)_2_	319, 331, 453, 476			[6f]
Au_8_(PPh_3_)_8_(NO_3_)_2_	375, 415, 450	1.97	734	[25c]
Au_9_(PPh_3_)_8_(NO_3_)_3_	315, 352, 375, 443	1.78^a)^	630	[25a]
Au_9_(PPh_3_)_8_Cl	296, 340, 377, 433, 538, 588			[52]
[PdAu_8_(PPh_3_)_8_]Cl_2_	299, 310, 347, 415, 463, 525	1.66^a)^, 1.95		[55]
[PdAu_8_(PPh_3_)_8_](NO_3_)_2_	299, 311, 347, 413, 460, 526			[56]
Au_11_(PPh_3_)_7_Cl_3_	240, 308, 420	2.15^a)^, 2.0		[25, 67, 303]
Au_11_(PPh_3_)_7_Br_3_	293, 309, 383, 422, 515			[162]
Au_11_(PPh_3_)_7_I_3_	293, 420	1.87		[200]
[Au_11_(PPh_3_)_8_Cl_2_]Cl	240, 312, 416	2.09^a)^		[67, 303]
Au_8_Ag_3_(PPh_3_)_7_Cl_3_	417, 500, 656	1.69, 1.67^b)^	405, 434, 454	[58]
PdAu_10_(PPh_3_)_8_Cl_2_	n/a		950	[64]
Au_6_(dppp)_4_(NO_3_)_2_	326, 432, 587		834	[51, 131]
Au_7_(dppp)_4_(BF_4_)_3_	556		642	[49]
[Au_8_(dppp)_4_Cl_2_]Cl_2_	322, 390, 508		597	[34, 51, 131]
Au_8_(dppp)_4_(NO)_2_	308, 520, 590			[34, 51]
[Au_8_(dppm)_4_S_2_]Cl_2_	327, 342		591	[81]
Au_11_(dppe)_6_(SbF_6_)_3_	316, 390, 471, 663			[74, 131]
[Au_11_(DPEphos)_4_Cl_2_]Cl	285, 309, 456	1.83^b)^		[72]
[Au_11_(Xantphos)_4_Cl_2_]Cl	323, 362, 427	1.26^b)^		[72]
[Au_13_(PMe_2_Ph)_10_Cl_2_](PF_6_)_3_	296, 338, 428			[134a]
[Au_13_(PMePh_2_)_9_Cl_3_](PF_6_)_2_	300, 340, 420			[134a]
[Au_13_(dppe)_5_Cl_2_]Cl_3_	304, 359, 493	1.90	766	[34, 80, 253]
[Au_13_(dppp)_4_Cl_4_]Cl	340, 430		780	[34, 201]
[Au_13_Cu_2_(PPh_3_)_6_(SPy)_6_]^+^	337, 406, 475, 575		1006	[83]
[Au_13_Cu_4_(PPh_3_)_4_(SPy)_8_]^+^	258, 339, 403, 463		965	[83]
[PdAu_13_(PPh_3_)_3_(SR)_7_]^+^	350, 390, 410, 460	2.70	810	[82]
E‐[Au_13_Ag_12_(PPh_3_)_10_Cl_8_]SbF_6_	361, 418, 497	2.17		[84]
S‐[Au_13_Ag_12_(PPh_3_)_10_Cl_8_]SbF_6_	330, 423, 510, 657	1.72		[84]
[Au_18_(dppm)_6_Cl_4_]Cl_3_PF_6_	325, 410, 443, 490, 616			[86]
[Au_19_(C≡CPh)_9_(Hdppa)_3_](SbF_6_)_2_	277, 388, 548, 934	1.13		[88]
[Au_20_(PPhpy_2_)_10_Cl_4_]Cl_2_	344, 493	2.24		[92]
[Au_20_(PP_3_)_4_]Cl_4_	272, 360, 488, 541	1.33		[202]
[Au_20_(PPh_3_)_12_H_3_](SbF_6_)_3_	357, 414, 488, 554, 608, 715	1.5		[20b]
Au_22_(dppo)_6_	456	1.9	700	[34, 93]
[Au_22_H_3_(dppee)_7_]^3+^	312, 381, 459			[95]
[Au_23_(PPh_3_)_6_(C≡CPh)_9_](SbF_6_)_2_	273, 380, 525, 600	2.02		[97]
[Au_24_(PPh_3_)_4_(C≡CPh)_14_](SbF_6_)_2_	266, 425, 630	1.75	925	[98]
[Au_24_(dppb)_6_Cl_4_]Cl_2_	433, 590, 692, 806			[99]
Au_25_(PPh_3_)_10_(SPh)_5_Cl_2_	426, 680	1.98		[147]
[Au_28_(PPh_3_)_9_(SR)_4_]^2+^	450, 580, 725	1.3		[105]
Au_32_(PEt_3_)_12_Cl_8_	480600, 662, 727			[106]
[Au_32_(PPh_3_)_8_(dpa)_6_](SbF_6_)_2_	370, 455, 535, 584, 631, 724, 978			[107]
Au_54_(PEt_3_)_18_Cl_12_	398, 438, 467, 525, 595732			[37]
Au_55_(PPh_3_)_12_Cl_6_	Featureless with tiny band *ca*. 510			[203]
Au_101_(PPh_3_)_21_Cl_5_	Featureless with tiny band *ca*. 520			[113]

^a)^ Electrochemical energy gap;

^b)^ DFT‐simulated energy gap.

**Figure 6:**
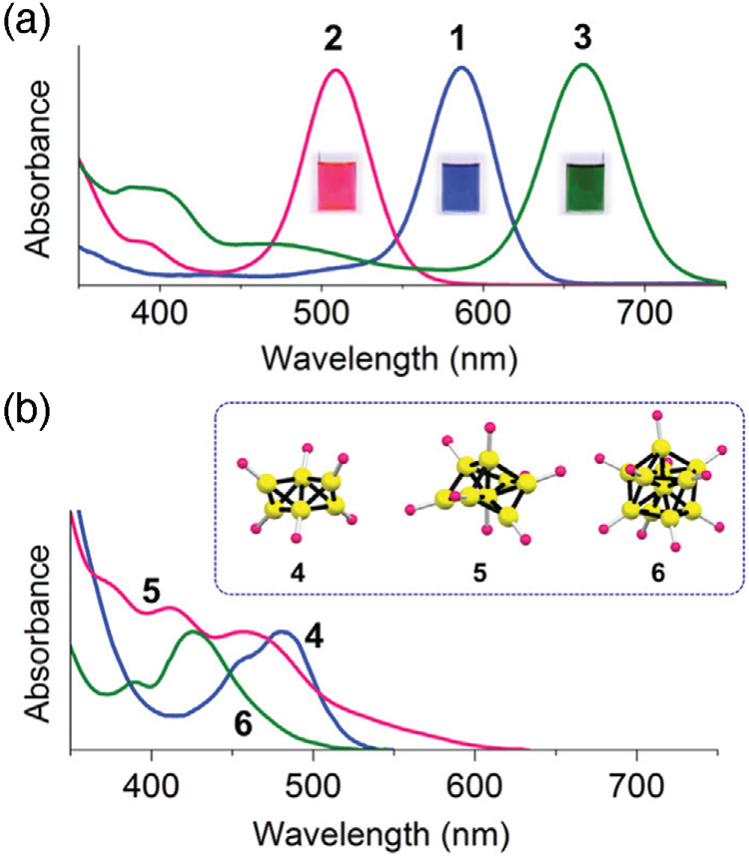
UV–visible spectra of [core+exo]‐type Au clusters for Au_6_ (**1**), Au_8_ (**2**) and Au_11_ (**3**) in (a), and closed‐polyhedra clusters for Au_6_ (**4**), Au_8_ (**5**) and Au_11_ (**6**) in (b). Reproduced with permission.^[^ ^131b^ ^]^ Copyright 2013, American Chemical Society.

In contrast to the conventional polyhedral clusters, Au clusters with [core+exo]‐type structures have quite different optical properties due to substantial contributions from the exo Au atoms. The [core+exo]‐type Au clusters usually feature the characteristic of an intense, isolated single peak in the visible region (see **Figure** **6**).^[^ ^131b^ ^]^ Konishi and co‐workers reported that attachment of just one exo Au atom to a polyhedral core, as in the case of [Au_7_(dppp)_4_](BF_4_)_3_, generates the unique absorption profile.^[^ ^49^ ^]^ Clusters of the same core nuclearity and charge state but different geometries, for example [Au_11_(dppe)_6_]^3+^ with two exo Au atoms, displays an intense peak at 663 nm while icosahedral [Au_11_(dppp)_5_]^3+^ lacks such a feature. The genesis of the intense, isolated peak is attributed to the HOMO–LUMO transition from the polyhedral core to the exo atoms, i.e., transition in the core → exo direction, where the HOMO and LUMO are localized, respectively. However, the HOMO–LUMO energy gaps do not scale with the core (size) nuclearity, indicating that the core geometry fundamentally determines the optical properties.

The photoluminescence properties of metal clusters have become the subject of growing interest as an alternative to toxic quantum dots. Au clusters with conventional polyhedral structures typically display negligible photoluminescence (PL), except for some systems (refer to Table 2). However, near‐IR luminescence in some Au clusters, in both phosphine‐ligated^[^ ^75^, ^83^, ^98^ ^]^ and thiolate‐protected analogs^[^ ^204^ ^]^ has been reported. The Au_9_(PPh_3_)_8_(NO_3_)_3_ cluster exhibits two weak, broad PL peaks at 579 and 853 nm in the solid state.^[^ ^25a^ ^]^ A near‐IR PL peak around 1080 nm has also been observed for Au_11_(PPh_3_)_7_Cl_3_. Heteroatom doping of Au clusters can modulate the electronic structure and thus the optical properties.^[^ ^205^ ^]^ For example, doping Ag atoms in undecagold to afford Au_8_Ag_3_(PPh_3_)_7_Cl_3_ results in three PL peaks 405, 434, and 454 nm with enhanced intensity.^[^ ^58^ ^]^ Similarly, PdAu_10_(PPh_3_)_8_Cl_2_ also shows a strong emission at 950 nm.^[^ ^64^ ^]^

In contrast to conventional polyhedral clusters, many [core+exo]‐type Au clusters exhibit PL in the visible region. A single PL peak was observed for [Au_7_(dppp)_4_](BF_4_)_3_ and [Au_8_(dppp)_4_Cl_2_](PF_6_)_2_ at 642 and 597 nm, respectively. Ab initio calculations suggested phosphorescence as the dominant emission mechanism.^[^ ^206^ ^]^ While absorption profiles are less sensitive to different phosphine ligands with the same core moiety, the PL properties are markedly affected and different.^[^ ^152^ ^]^ Using [Au_6_(dppp)_4_]^2+^ and [Au_6_(dppb)_4_]^2+^ as examples, Shichibu et al. demonstrated that the former exhibited dominant phosphorescence at 834 nm while the latter showed both fluorescence and phosphorescence at 655 and 900 nm, respectively.^[^ ^131a^ ^]^ Time‐resolved PL measurements revealed that the visible emission has a short lifetime (<100 ps) and near‐IR emission in the order of microseconds. Interestingly, phosphorescence can be induced by aggregation of clusters in solution and solid state, as has been found in [Au_6_]^2+^ and [Au_8_]^4+^.^[^ ^207^ ^]^ Restricted motions/vibrations in aggregates are believed to induce triplet states that are responsible for phosphorescence. In fact, different modes of aggregation due to stacked orientation and ordered packing give rise to different optical responses.^[^ ^207b^ ^]^

The steady‐state absorption and fluorescence spectra provide an overview of the photophysical processes that molecules undergo upon light irradiation. On the other hand, transient absorption spectroscopy (TAS) provides a set of techniques for probing and characterizing the electronic properties of short‐lived excited states (transient states).^[^ ^208^ ^]^ When photons with energy greater than the HOMO–LUMO gap of Au nanoclusters are irradiated, the excitons dissociate and form free electrons and holes.^[^ ^209^ ^]^ The subsequent relaxation of the electrons and holes occurs on the subpico‐ to microsecond time scales. Time‐resolved techniques such as TAS have been used extensively to probe relaxation dynamics in heavy metal nanoclusters, which may be utilized in energy harvesting applications.^[^ ^210^ ^]^ TAS can probe the difference between the absorption of ground and excited states in the visible and infrared (IR) region, and does not require the sample to be luminescent as most Au nanoclusters (NCs) have low quantum yields. To determine the relaxation time constants, global fitting of the kinetics probed at all wavelengths is carried out. There have been several studies that propose relaxation mechanisms with different timescales for phosphine‐stabilized Au clusters.^[^ ^211^ ^]^

Smith et al. performed TAS on both Au_55_(PPh_2_P(m‐C_6_H_4_SO_3_Na))_12_Cl_16_ and Au_13_(dppm)_6_(NO_3_)_4_ which were both pumped at 390 nm and probed at 780 nm.^[^ ^212^ ^]^ The larger cluster exhibited a single exponential decay with a time constant of 1 ps while the smaller cluster required fitting with both fast (1 ps) and slow (300 ps) components. The size dependence of the electronic relaxation time is influenced by both weakened electron‐phonon coupling and enhanced surface collisions for smaller clusters. In this study, they concluded that the slower decay of Au_13_ is characteristic of molecular behavior. Jin and co‐workers reported ≈100 ps exciton localization between the two vertices of the triicosahedral [Au_37_(PPh_3_)_10_(SC_2_H_4_Ph)_10_X_2_]^+^ cluster which they assigned to structural distortion induced by strong excited state coherent vibrations.^[^ ^213^ ^]^ However, this phenomenon was not observed for the dimer [Au_25_(PPh_3_)_10_(SC_2_H_4_Ph)_5_Cl_2_]^2+^, which contains one vertex between two Au_13_ icosahedral units. Solvent has also been found to play an important role in TAS studies. The solvent stabilization of the intramolecular charge transfer state (ICT) in [Au_20_(PPhpy_2_)_10_Cl_4_]Cl_2_ was found to be faster in methanol than ethanol and propanol.^[^ ^214^ ^]^ The transient absorption study of [Au_9_(PPh_3_)_8_]^3+^ in methanol and dichloromethane showed relatively fast S_1_ relaxation decay times of 2 ps and 45 ps which were assigned to intersystem crossing (S_1_ → T_1_) and nonradiative relaxation (S_1_ → S_0_), respectively.^[^ ^215^ ^]^

### 3.3 Chirality

Chirality plays a critical role in asymmetric catalysis, drug design, chiral molecular recognition/sensing, and chiroptical materials. A chiral compound has a pair of enantiomers with non‐superimposable mirror images. Chirality can be quantified geometrically using the Hausdorff chirality measure to classify and determine the degree of chirality.^[^ ^216^ ^]^ Chirality in clusters can be introduced by preparing intrinsically chiral Au cores or imparting chiral modifiers (ligands, ion‐pairing) onto an achiral core to create a chiral environment.^[^ ^217^ ^]^ The chiroptical activity of chiral clusters is studied and verified using circular dichroism (CD) spectroscopy. Enantiomeric pairs display Cotton effects and a mirror image relationship in a CD spectrum. For atomically precise Au clusters, the total structure can be established completely by single‐crystal X‐ray diffraction, which enables precise determination of the genesis of chirality.

**Figure 7:**
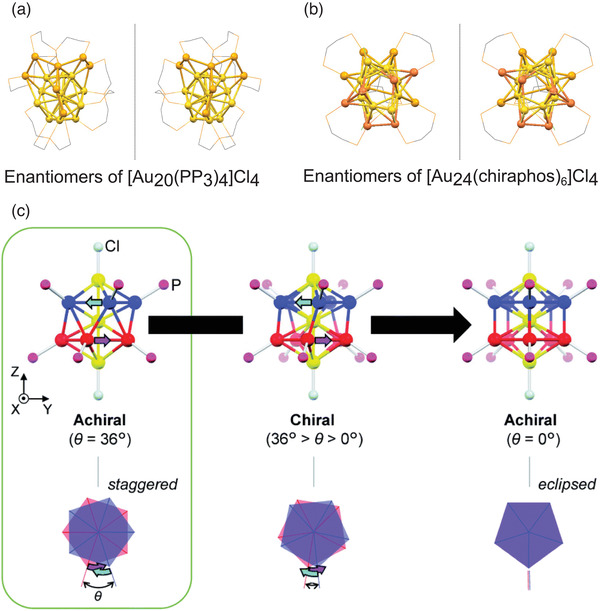
Core framework of enantiomeric pairs of a) Au_20_(PP_3_)_4_Cl_4_ and b) Au_24_(chiraphos)_6_Cl_4_ clusters. c) Achiral gold atoms are shown in yellow, chiral gold strands/motifs in orange and/or salmon. Hydrogen and phenyl rings are omitted for clarity. Torsional twist of the two pentagonal, equatorial Au_5_ rings in the Au_13_ core, characterized by the torsional angle, *θ*, leading to the achiral or chiral nature. Reproduced with permission.^[^ ^219^ ^]^ Copyright 2021, Royal Society of Chemistry.

The first intrinsic chiral core cluster protected by achiral phosphine ligands was observed in Au_20_(PP_3_)_4_Cl_4_ in 2014. A successful synthesis and structural determination revealed the crystal structure consisting of an icosahedral Au_13_ unit and an outer surface tripodal Au_7_ motif with a local C_3_ symmetry as illustrated in **Figure** **7**a.^[^ ^202^, ^218^ ^]^ The chirality is associated with the Au_7_ motif capping the icosahedral core. The Au_20_ cluster exists in both enantiomeric forms leading to a racemic mixture in the synthetic preparation requiring isolation of enantiopure clusters. Recently, a successful separation of enantiopure right‐handed Au_20_ (R‐Au_20_) clusters was achieved by Zhu et al. utilizing *α*‐cyclodextrin (*α*‐CD) with enantiomer excess value up to 97%.^[^ ^91^ ^]^ Extensive molecular docking and quantum chemical calculations revealed that R‐Au_20_ inside *α*‐CD gives a more stable adduct due to the larger number of O∙∙∙H bonds inside the cavity. Such preference allows efficient separation of both enantiomers where R‐Au_20_ is isolated as R‐Au_20_(*α*‐CD) leaving L‐Au_20_ in the solution.

A chiral core can also be formed using chiral phosphine ligands where the outer gold motif forms a chiral geometry. Importantly, the use of chiral ligands may direct preparation of enantiopure gold clusters which is termed enantioselective synthesis. This method circumvents the typical issue of the existence of enantiomeric pairs in the crystalline samples which hinders precise characterization of enantiopure clusters. Konishi and co‐workers reported an enantioselective synthesis of novel, enantiopure Au_24_ frameworks using chiral 2,3‐bis(diphenylphosphino)butane or chiraphos.^[^ ^99^ ^]^ The crystal structure consists of an achiral, square antiprism‐like Au_8_ core with a duplex of helical hexagold strands, shown in Figure 7b). The intrinsic chirality of the Au_24_ framework is determined by the helical hexagold atoms ligated to chiraphos where (R,R)‐ and (S,S)‐chiraphos give right‐ and left‐handed strands, respectively.

Very recently, the preparation of enantiopure icosahedral Au_13_ clusters stabilized by chiral 1,2‐bis[(2‐methoxyphenyl)phenylphosphino]ethane or OMe‐dppe was achieved using enantioselective synthesis.^[^ ^220^ ^]^ The highly symmetric Au_13_ kernel is distorted by a helical ligand arrangement inducing chirality and contributing to the chiroptical activity in the core‐based transitions. The chirality of the Au_13_ cluster is dictated by the chirality of the ligands where (R)‐ and (L)‐OMe‐dppe ligands yield right‐ and left‐handed Au_13_ clusters, respectively. Distortion of the gold core can also be achieved using achiral ligands. Similarly, a small torsional distortion between two equatorial, pentagonal Au_5_ rings induced by achiral diphosphine (dppe) give rise to chirality of the Au_13_ core.^[^ ^219^ ^]^ The torsional angle that gives rise to chirality lies between 0° and 36° (Figure 7c). A recent synthesis of [Au_9_Ag_12_(dppm)_6_(SR)_4_X_6_]^3+^ (X = Cl/Br) by Zhu and co‐workers yielded both achiral and chiral isomers.^[^ ^124^ ^]^ The authors showed that the icosahedral Au_5_Ag_8_ kernels are the same for both structural isomers while the arrangement of the four surface Au atoms on the kernel distinguishes the achiral and chiral kernel structures.

Chiral bidentate phosphine (2,2’‐bis(diphenylphosphino)‐1,1’‐binaphthyl) (BINAP) is commonly used in enantioselective synthesis of chiral clusters and nanoparticles. Similar to chiraphos, chirality of BINAP is transferred to the clusters where the handedness of Au clusters depends on that of BINAP. Importantly, BINAP plays two key roles in inducing chirality in metal clusters. First, it causes structural deformation of the Au core upon ligation,^[^ ^221^ ^]^ and second, it enhances the optical rotation strength in the CD spectra due to diffuse *π*‐electrons in chiral binaphthyl close to the Au core.^[^ ^222^ ^]^ Tsukuda and co‐workers reported that the optical activity in the visible region for Au_8_ and Au_11_ clusters originates from the core‐based transitions, and is magnified by the chirality of BINAP.^[^ ^222a^ ^]^ For BINAP‐ligated Au_9_ and Au_10_ clusters, the optical activity has been ascribed to ligand‐to‐metal charge transfer (LMCT) with Au_10_ showing the largest anisotropy (g) value reported for gold clusters so far.^[^ ^223^ ^]^

## 4 Characterization of Au Clusters

State‐of‐the‐art characterization techniques are indispensable in studying the structural and fundamental properties of atomically precise Au clusters. Previously, reliance on elemental analysis had led to an incorrect assignment of molecular formula for the pentagold cluster;^[^ ^134b^ ^]^ it was later corrected to be undecagold. Apparently, no single characterization method can provide complete and conclusive information about material properties and characteristics. Thus, very often multiple, complementary techniques are required to gain information about their structure and properties. Progress in gold–phosphine clusters has benefited from numerous characterization tools, including spectroscopy, microscopy and single‐crystal X‐ray crystallography. This section discusses in detail several of the key techniques employed to characterize the size‐dependent and unique properties of gold–phosphine clusters which enables our understanding of these properties.

### 4.1 UV–Visible Spectroscopy

**Figure 8:**
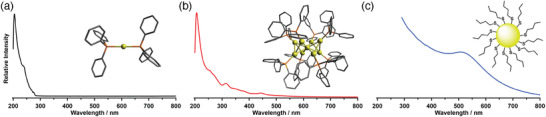
UV–visible spectra of a) Au(PPh_3_)_2_Cl, b) Au_9_(PPh_3_)_8_(NO_3_)_3_ and c) 5 nm thiolate‐protected plasmonic Au nanoparticles. Reproduced with permission.^[^ ^198^ ^]^ Copyright 2020, American Chemical Society.

UV–visible spectroscopy is a fundamental tool for studying the optical properties of Au clusters and nanoparticles and is utilized to distinguish between nanoparticles and clusters. Au clusters display molecular‐like optical properties with distinct peaks, while Au nanoparticles show plasmonic behaviors with a broad LSPR band. As discussed previously in subsection 3.2, the transition from the molecular cluster to the plasmonic nanoparticle for Au has been determined experimentally to occur around 2.2 nm. Thus, UV–visible spectroscopy enables the distinction between the cluster and nanoparticle regimes. Quantization of energy levels in Au clusters gives rise to discrete electronic transitions which manifest as distinct peaks in the UV–visible spectrum. For instance, **Figure** **8** shows the UV–visible spectrum of Au_9_(PPh_3_)_8_(NO_3_)_3_ clusters (size ≈0.8 nm) which consists of a few distinct peaks, while Au nanoparticles (size ≈5 nm) shows a broad localized surface plasmon resonance (LSPR) around 520 nm.^[^ ^198^ ^]^

Minute yet discernable differences can be observed for Au clusters with different phosphine ligands and counter anions. For example, Hutchison and co‐workers found a slight distinction in the absorbance spectra of Au_11_(PPh_3_)_7_Cl_3_ and [Au_11_(PPh_3_)_8_Cl_2_]Cl, as shown in Figure 1a. Similarly, tiny differences in the absorbance spectra of tridecagold clusters stabilized by different monophosphine ligands, [Au_13_(PMe_2_Ph)_10_Cl_2_](PF_6_)_3_ and [Au_13_(PMe_2_Ph)_9_Cl_3_](PF_6_)_2_, (see Table 2) are also observed.^[^ ^67^ ^]^

In situ UV–visible spectroscopy has been pivotal in studying kinetics of reduction and nucleation of Au clusters.^[^ ^224^ ^]^ A combination of UV–visible spectroscopy and mass spectrometry allows the study of the evolution of size‐selective Au clusters during synthesis by identifying peaks corresponding to specific gold–phosphine clusters, thus elucidating the reaction mechanism or pathway. Pettibone and Hudgens elucidated the mechanism of formation of Au*_x_* (*x* = 8, 9) clusters via borohydride reduction.^[^ ^142^ ^]^ The authors found that fast reduction initially produced large nanoparticles as signified by the appearance of an LPSR band and gradual formation of Au clusters as evidenced by the distinct, multiple peaks in the absorption spectra. Such information enables the authors to unravel a cyclic process of growth and etching responsible for size‐selective formation of Au clusters.

Determination of the energy gap in the HOMO–LUMO transition is also made possible using UV–visible spectroscopy. The bandgap can be estimated by plotting the absorbance versus energy, and the tangent to the curve that intercepts with the energy axis (x‐axis) represents the HUMO–LUMO transition. The wavelength is converted into energy using the equation *E*(eV)  =  1240/*λ*(nm). Simon and co‐workers measured the optical absorption of Au_11_(PPh_3_)_7_Cl_3_ and estimated the energy gap of the first electronic transition to be ≈2.0 eV,^[^ ^25c^ ^]^ which matched closely with the theoretical calculation of the simplified Au_11_(PH_3_)_7_Cl_3_ structure by Häkkinen and co‐workers.^[^ ^173a^ ^]^ The energy of other optical transitions can be estimated by deconvolution of the spectral profile.^[^ ^225^ ^]^

Finally, for solid samples like supported Au cluster catalysts, UV–visible diffuse reflectance spectroscopy (UV–vis DRS) is useful for studying the evolution and aggregation of Au clusters. Liu et al. measured the optical absorbance of PPh_3_‐stabilized Au_11_ clusters supported on SBA‐15.^[^ ^226^ ^]^ The characteristic peak of Au_11_ clusters at 415 nm (in solution) is red‐shifted and broadened upon deposition onto the SBA‐15 support due to the change in the electronic structure of the Au_11_ cluster when interacting with the support.^[^ ^227^ ^]^ Additionally, the disappearance of sharp and distinct peaks and the emergence of the LSPR band are indicative of the size evolution from clusters (<2.2 nm) into nanoparticles (>2.2 nm).

### 4.2 Electron Microscopy

Progress of nanoscale science and technology is heavily dependent on state‐of‐the‐art characterization techniques. Very often, a method by which one can directly “see” nanoscale materials provides conclusive evidence of their existence compared to indirect methods—*seeing is believing*. Transmission electron microscopy (TEM) and scanning transmission electron microscopy (STEM) are widely used techniques for imaging an electron‐transparent sample at the atomic scale. The difference between the two techniques is that STEM uses a small electron beam that is prefocused before imaging the sample, while the large electron beam used for TEM is focused after transmission through the sample.^[^ ^228^ ^]^ The high‐angle annular dark‐field (HAADF) method in STEM is predicated on detecting the incoherent scattering electrons, referred to as Z‐contrast microscopy. STEM has higher resolution imaging and analytical capabilities due to the use of aberration correctors. Advances in aberration corrector electron optics allow direct imaging at the atomic level with high resolution beyond 50 pm.^[^ ^229^ ^]^ Due to its high atomic number, gold can be easily imaged at high contrast using TEM. With aberration correction, imaging of Au clusters at the atomic resolution is now possible.^[^ ^230^ ^]^ Information that can be obtained from TEM and STEM imaging includes particle size, shape, lattice fringe, crystallographic structure, defects, and grain boundaries.

TEM images of Schmid's Au_55_ cluster (the average formula is Au_55_(PPh_3_)_12_Cl_6_)) revealed the particle size to be 1.4 ± 0.4 nm.^[^ ^231^ ^]^ Initial assignments based on EXAFS attributed the Au core structure to have cuboctahedral geometry,^[^ ^232^ ^]^ however, an icosahedral structure was also suggested to be plausible by powder X‐ray diffraction.^[^ ^233^ ^]^ Recently, thorough and systematic studies using aberration‐corrected STEM by Jian et al. discovered that Au_55_ clusters contain both hybrid icosahedron–cuboctahedron and amorphous structures, resolving the contradictory studies previously reported.^[^ ^24^ ^]^ Based on the intensity representing the number of clusters as a function of core size, the authors found that the four most intense peaks occurred at 41 ± 2, 47 ± 1.5, 50 ± 1.5 and 54 ± 1.5 Au atoms, with the last peak being assigned to Au_55_(PPh_3_)_12_Cl_6_.

**Figure 9:**
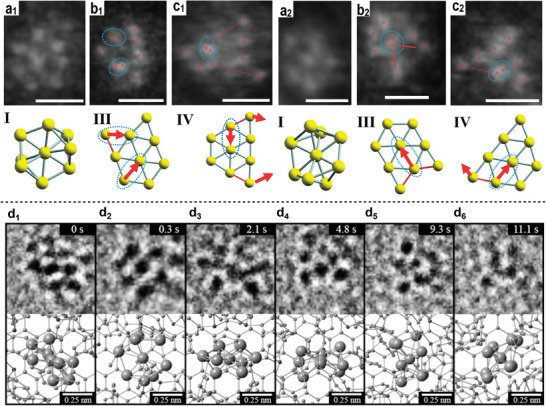
a–c) STEM‐HAADF images of Au_9_ supported on TiO_2_ clusters with different structures. Reproduced with permission.^[^ ^237^ ^]^ Copyright 2016, American Institute of Physics. d) Dynamic motion of Au_9_ covalently bonded to the surface of graphene. Reproduced with permission.^[^ ^238^ ^]^ Copyright 2015, Wiley VCH.

The fluxionality of Au clusters on surfaces renders precise structural determination difficult,^[^ ^234^ ^]^ particularly because electron beams can affect the geometry and induce fluctuation between isomers.^[^ ^235^ ^]^ Wang and Palmer observed that the structure of Au_20_ clusters fluctuates between tetrahedral and disordered structures.^[^ ^236^ ^]^ Deposition of Au clusters on surfaces also reduces the degree of freedom. A study by Al Qahtani et al. investigated the structure of Au_9_(PPh_3_)_8_(NO_3_)_3_ clusters on TiO_2_ nanosheets.^[^ ^237^ ^]^ They observed three atomic configurations using STEM (**Figure** **9**a–c); one 3D structure and two pseudo‐2D structures. The 3D structure was attributed to Au_9_ protected with ligands, while the pseudo‐2D structures were attributed to de‐ligated clusters with intrinsic fluxionality.

Rourke and co‐workers used STEM to image Au_9_(PPh_3_)_8_(NO_3_)_3_ clusters attached to sulfur‐functionalized graphene oxide (GOSH).^[^ ^238^ ^]^ Time‐dependent rotations of Au_9_ cluster covalently bonded to the surface of GOSH are shown in Figure 9d. This result shows the effect of the electron beam on a single Au_9_ cluster inducing rotation of the cluster, without any lateral displacement, during the imaging. This also demonstrates that Au_9_ clusters attached to GOSH are robustly bound even under the effect of an intense STEM electron beam.

### 4.3 Photoelectron Spectroscopy (PES)

Photoelectron spectroscopy (PES) is an important and powerful tool to investigate the surface properties of materials down to a few nanometers in depth. The surface sensitivity of PES is due to the limitation of the emitted electron mean free path, where an electron excited by a photon will lose energy before leaving the surface to reach the detector. PES is classified into two energy regimes: X‐ray photoelectron spectroscopy (XPS) and ultraviolet photoelectron spectroscopy (UPS).

XPS is a widely used technique to investigate the chemical composition and elemental concentration of a surface. The technique measures the kinetic energy of electrons emitted from the top layer (<5 nm) of the surface.^[^ ^239^ ^]^ It is employed to determine the electronic structure, elemental composition, and chemical environment of various elements present in the sample via the binding energy (BE), full width at half maximum (FWHM), and intensity of the corresponding elemental peak.

For gold, the core electrons usually analyzed in XPS originate from the Au 4f orbitals. The Au 4f doublet peak (4f_7/2_ and 4f_5/2_) in an XPS spectrum arises due to the quantum mechanical nature of the spin–orbit coupling. For bulk gold, the literature standard BE of the Au 4f_7/2_ peak is 84.0 eV with a separation of 3.67 eV to the Au 4f_5/2_ peak.^[^ ^240^ ^]^ The BEs for Au clusters are significantly different from the bulk value. Such differences are attributed to the initial and final state effects, which strongly influence the peak position and FWHM.^[^ ^241^ ^]^

The initial state effect reflects the oxidation state of the metal. The BE is positively charged for oxidized metal due to the loss of charge to oxygen, which reduces the core state energy, leading to an increase in the electron BE.^[^ ^242^ ^]^ The final state effect is due to the difficulty of the charged atom being relaxed by residual electrons following excitation by an X‐ray beam.^[^ ^243^ ^]^ When an electron is excited, the positively charge hole remains in a charged state for a finite time and the residual electrons redistribute to screen the positively charge hole.^[^ ^242^, ^244^ ^]^ The initial and final state effects lead to a shift toward a higher BE, referred to as a positive BE shift. Several examples using XPS to study the change in the chemical state of Au and Au cluster size are given below.^[^ ^245^ ^]^

**Table** **3** summarizes the BE of Au 4f_7/2_ for different gold compounds and several atomically precise gold–phosphine clusters reported to date. Upon formation of oxidized species such as Au^+^ and Au^3+^, the Au 4f_7/2_ peak shifts to 85–87 eV, depending on the composition of the gold compound, due to the initial state effect.^[^ ^246^ ^]^ For this reason, XPS can be used to trace the formation of Au metal nanoparticles from Au precursors during the synthesis by measuring the change in the BE of the Au 4f peak. For example, Visco et al. probed the oxidation state of Au prepared by different synthetic methods and pretreatment using XPS.^[^ ^247^ ^]^ The authors found that thermal treatment under conditions such as vacuum, H_2_, and H_2_ followed by O_2_, were required to transform gold hydroxide (from reaction of HAuCl_4_ with OH^−^) into metallic gold (Au^0^) as manifested by the simultaneous decrease and increase of the Au^3+^ and Au^0^ peak areas, respectively.

**Table 3:** BE of Au 4f_7/2_ for different gold compounds and several gold–phosphine clusters reported up‐to‐date

Compound	Au 4f_7/2_ [eV]	Calibrated against	Excitation energy	Refs.
Au metal	84.0	C 1s peak at 284.8 eV	Mg‐K*α* (1253.6 eV)	[240b]
HAuCl_4_	87.3–87.6	C 1s peak at 285 eV	Al‐Kα (1486.6 eV)	[247]
Au_2_O_3_	85.5–86.3	C 1s peak at 285 eV	Al‐Kα (1486.6 eV)	[247]
AuCl	85.8–86.0	C 1s peak at 285 eV	Al‐Kα (1486.6 eV)	[247]
Au(PPh_3_)Cl	85.3	No information provided	No information provided	[50]
Au(PPh_3_)_2_Cl	85.6	No information provided	No information provided	[50]
Au(PPh_3_)NO_3_	84.9	C 1s peak at 284.6 eV	Mg‐Kα (1253.6 eV)	[248]
Au_5_Cu_6_(Dppf)_2_(SAdm)_6_(BPh_4_)	84.7	No information provided	Al‐Kα (1486.6 eV)	[45]
Au_6_(PPh_3_)_6_(BF_4_)_2_	84.7	C 1s peak at 284.7 eV	Mg‐Kα (1253.6 eV)	[249]
[Au_8_(PPh_3_)_7_]^2+^	85.2	No information provided	No information provided	[50]
Au_8_(PPh_3_)_8_(NO_3_)_2_	85.1	C 1s peak at 285 eV	Synchrotron (690 eV)	[15]
Au_8_(TPPMS)_x_(NO_3_)_2_ with x = 7,8	84.5	C 1s peak at 284.8 eV	Al‐Kα (1486.6 eV)	[250]
Au_9_(PPh_3_)_8_(NO_3_)_3_	84.7–85.3	C 1s peak at 285 eV	Mg‐Kα (1253.6 eV) Synchrotron (625 eV) Synchrotron (690 eV)	[15, 237, 251]
Au_11_(PPh_3_)_8_Cl_3_	84.7	C 1s peak at 285 eV	Mg‐Kα (1253.6 eV)	[15, 50]
Au_11_(PPh_3_)_7_I_3_	84.5	No information provided	Mg‐Kα (1253.6 eV)	[200]
Au_13_(dppe)_5_Cl_2_Cl_3_	85.1–85.6	C 1s peak at 285 eV	Mg‐Kα (1253.6 eV)	[252]
[Au_13_(dppe)_5_Cl_2_]^3+^	84.4	C 1s peak at 284.8 eV	Al‐Kα (1486.6 eV)	[253]
Au_13_Ag_12_(PPh_3_)_10_Cl_8_	84.4	C 1s peak 284.6 eV	Al‐Kα (1486.6 eV)	[254]
[Au_19_Cu_30_(PPh_3_)_6_(C≡CPh)_22_Cl_2_](NO_3_)_3_	84.3	C 1s peak at 284.6 eV	Al‐Kα (1486.6 eV)	[89]
Au_20_(PP_3_)_4_Cl_4_	84.3	C 1s peak at 284.6 eV	Al‐Kα (1486.6 eV)	[202]
[Au_23_(PPh_3_)_6_(C≡CPh)_9_](SbF_6_)_2_	84.4	C 1s peak at 284.6 eV	Al‐Kα (1486.6 eV)	[97]
[Au_24_(PPh_3_)_4_(C≡CPh)_14_](SbF_6_)_2_	84.5	C 1s peak at 284.6 eV	Al‐Kα (1486.6 eV)	[98]
[Au_24_Pd(PPh_3_)_10_(SR)_5_Cl_2_]Cl	84.5	Ag 3d_5/2_ peak at 367.9 eV	Mg‐Kα (1253.6 eV)	[100]
[Au_25_(PPh_3_)_10_(SR)_5_Cl_2_](SbF_6_)_2_	84.9	Ag 3d_5/2_ peak at 367.9 eV	Mg‐Kα (1253.6 eV)	[100]
Au_25_(PPh_3_)_10_(SC_12_H_25_)_5_Cl_2_	84.3	C 1s peak at 284.8 eV	Al‐Kα (1486.6 eV)	[255]
[Au_32_(PPh_3_)_8_(dpa)_6_](SbF_6_)_2_	84.6	C 1s peak at 284.6 eV	Al‐Kα(1486.6 eV)	[107]
Au_55_(PPh_3_)_12_Cl_6_	84.3	No information provided	Al‐Kα (1486.6 eV)	[256]
Au_55_(PPh_3_)_12_Cl_6_	85.1^a)^	No information provided	Al‐Kα (1486.6 eV)	[257]
Au_101_(PPh_3_)_21_Cl_5_	83.8–83.7	C 1s peak at 285 eV	Mg‐Kα (1253.6 eV) Synchrotron (690 eV)	[15, 258]

^a)^ The binding energy published in ref. [257] for Au clusters is significantly different to other published binding energies for Au clusters of similar size, e.g. in refs. [15, 256].

The use of XPS is not limited to determining the chemical states and composition but can also be used to determine the relative size of Au clusters due to the final state effect. Haruta used the positive shift in the BE to assign small Au clusters as the most active sites in gold‐based catalysts; at the time of that study, access to high‐resolution TEM was extremely limited.^[^ ^259^ ^]^ Early XPS studies of phosphine‐ligated Au clusters were performed and reported by Battistoni et al.^[^ ^260^ ^]^ The authors observed a general trend of positive BE shifts of the Au 4f_7/2_ peak as the number of Au atoms in the cluster decreased. However, the interpretation of XPS spectra of Au clusters at that time attributed the variation to changes in the cluster geometry and ligand components.^[^ ^261^ ^]^ Similar contributions have been made by Van Attekum et al. using Au_11_, Au_9,_ and Au_8_ clusters stabilized by triarylphosphine ligands.^[^ ^262^ ^]^ The authors showed that the BE of Au 4f peaks shifted to higher BE and the width (FWHM) broadens as the gold cluster size decreases.

An early XPS report of the final state effect on phosphine‐stabilized Au clusters was done by Schmid and co‐workers on Au_55_(PPh_3_)_12_Cl_6_.^[^ ^256^ ^]^ The authors suggested that the Au 4f peak shifted to higher BE compared to bulk Au due to the final state effect, following what had been discussed in earlier publications.^[^ ^263^ ^]^ A number of other XPS studies on Au_55_ can be found here.^[^ ^264^ ^]^ However, several XPS studies have investigated the electronic structure of new phosphine‐ligated Au clusters according to final state effect which differ from the interpretation of Schmid and co‐workers for Au_55_(PPh_3_)_12_Cl_6_. These studies were performed on Au_13_,^[^ ^253^ ^]^ bimetallic Au/Ag,^[^ ^265^ ^]^ Au_5_Cu_6_^[^ ^45^ ^]^ and Au_8_^[^ ^266^ ^]^ clusters, and all observed a shift in the BE of Au 4f_7/2_ peak to higher BEs due to the formation of the small‐sized clusters.

XPS studies have been performed on supported Au clusters to investigate the effect of supporting metal oxides on the clusters. In a study by Goodman and co‐workers, Au_6_(PPh_3_)_6_(BF_4_)_2_ was deposited on a single crystal TiO_2_(110) surface using a solution deposition method.^[^ ^249b^ ^]^ The XPS of Au 4f_7/2_ peak experienced a shift of +0.4 eV after removal of phosphine ligands via electron‐stimulated desorption. This shift was due to the size reduction of Au clusters, which was observed by scanning tunneling microscopy (STM). The same group reported that different pre‐treatment procedures of Au_6_ deposited on TiO_2_ particles had a profound influence on the stability of Au clusters on the surface of TiO_2_, which played a crucial role on CO oxidation activity.^[^ ^249a^ ^]^

**Figure 10:**
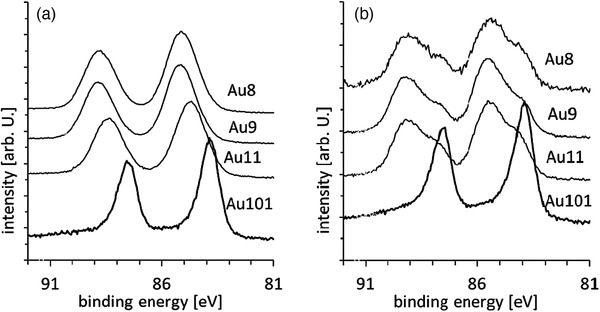
Au 4f peaks of TPP‐ligated Au*_x_* (*x* = 8,9,11 and 101) clusters on TiO_2_ (a) untreated and (b) calcined at 200 °C. Reproduced with permission.^[^ ^15a^ ^]^ Copyright 2013, Royal Society of Chemistry.

Recent studies by Anderson et al. utilized synchrotron XPS of Au_8_, Au_9_, Au_11_ and Au_101_ clusters supported on TiO_2_.^[^ ^15^ ^]^ **Figure** **10** shows the Au 4f spectra of untreated and calcined Au clusters.^[15a]^ The authors concluded that a positive shift in the BE and an increase in the FWHM of Au 4f_7/2_ peak were due to a decreasing number of Au atoms. Moreover, the as‐deposited Au clusters were not affected by the TiO_2_ substrate. However, calcination of the sample to remove the ligands lead to a degree of aggregation of Au clusters, as observed in the Au 4f spectra with two doublets of Au peaks. Al Qahtani et al. studied the aggregation of Au_9_ on TiO_2_ nanosheets and atomic layer deposition (ALD) TiO_2_.^[^ ^251c,d^ ^]^ They demonstrated that the Au 4f_7/2_ peak shifted to a lower BE after calcination due to aggregation of the Au_9_ clusters. This finding was supported by other techniques such as atomic force microscopy (AFM) and STM. One XPS study by Ruzicka et al. applied several pre‐treatment methods to TiO_2_ before depositing Au_9_ clusters to improve the Au cluster stability on the surface. They demonstrated that Au_9_ aggregation could be prevented, even under calcination, via pre‐treatment of the TiO_2_ with H_2_SO_4_, by observing a slight shift of the Au 4f_7/2_ peak to a higher BE after calcination.^[^ ^251e^ ^]^ Further reports of XPS studies performed to examine the electronic structure of gold–phosphine clusters by different groups are available here.^[^ ^28^, ^251^, ^252^, ^258^, ^267^ ^]^

In many cases, XPS is also useful to determine removal of phosphine ligands. Several XPS studies show that the P 2p peak shifts to higher BEs due to the removal of phosphine ligands from the Au core and subsequent surface oxidation upon calcination.^[^ ^15^, ^248^, ^249^, ^251^, ^252^, ^267^ ^]^ The use of XPS to study phosphine‐ligated Au clusters is not only limited to initial and final state effects. Ahmad et al. used XPS to estimate the concentration of Au on the surface of WO_3_ deduced from the electron mean free path.^[^ ^268^ ^]^

Very recently, XPS was used to determine the size of Au_9_(PPh_3_)_8_(NO_3_)_3_ clusters deposited on TiO_2_ film followed by photodeposition of a thin Cr_2_O_3_ layer.^[^ ^251f^ ^]^ Without the Cr_2_O_3_ layer, the XPS of Au 4f_7/2_ peak shifts to lower BE after the removal of phosphine ligands, indicating that the Au clusters have agglomerated. However, with the Cr_2_O_3_ layer the peak remains at higher BE. This demonstrates that Cr_2_O_3_ can act a protective layer to prevent cluster agglomeration after removal of the phosphine ligands.^[^ ^251f^ ^]^

UPS is a technique to study the electronic properties of a solid sample surface to a depth of 2–3 nm.^[^ ^239^ ^]^ It can be used to determine the valence band and the work function of a solid sample surface. It is an important technique to study the behavior of phosphine‐ligated Au clusters as clusters or metals. UPS has been employed to measure the electronic structure of individual phosphine‐protected Au clusters. A study by Boyen et al. investigated the valence band structure of the Au_55_(PPh_3_)_12_Cl_6_ cluster and compared it to Au_55_ after removal of ligands by exposure to X‐ray photons over a long period of time.^[^ ^264b^ ^]^ They concluded from the UPS valence band spectrum that Au_55_(PPh_3_)_12_Cl_6_ has an insulating behavior; however, after ligand removal the spectrum shifted toward the Fermi energy with a similar spectral profile to Au films. It was concluded that the Au_55_ cluster exhibited metallic behavior after ligand removal due to the large size of Au_55_ (1.4 nm). Recent research performed on the [Au_13_(dppe)_5_Cl_2_]^3+^ cluster determined that the valence band was 1.9 eV, which was confirmed by UV–visible spectroscopy and DFT simulations.^[^ ^253^ ^]^

Another powerful technique to measure the electronic structure is metastable impact electron spectroscopy (MIES) using metastable helium atoms (He*). The great benefit of MIES is that it only measures the electronic structure of the outermost layer of a sample. This is due to the He* deexcitation process that only occurs within a few Å of the surface, which leads to emission of an electron with kinetic energy that can be measured.

The first MIES study on Au clusters was performed on phosphine‐ligated Au clusters supported on ALD TiO_2_ and SiO_2_ via dip‐coating conditions and followed by heating to remove the ligands.^[^ ^269^ ^]^ The reaction between the phosphine‐ligated Au clusters and the two substrates after removal of the ligands was investigated using MIES and synchrotron XPS. It was found that the phosphine ligands react with the oxygen atoms of TiO_2_ after heating, leading to oxidation of the phosphine species. Nonagglomerated clusters were only found for samples deposited with a concentration between 0.02 × 10^‐3^ and 0.75 × 10^‐3^ m. In contrast, on SiO_2_ there was no sign of interaction between the phosphine ligands and substrate and the Au clusters were fully agglomerated to large nanoparticles after heating.

Krishnan et al. performed MIES studies on Au_9_(PPh_3_)_8_(NO_3_)_3_ and Au_13_(dppe)_5_Cl_2_Cl_3_ deposited with several concentrations onto defect‐rich ALD TiO_2_.^[^ ^252^, ^267^ ^]^ It was found that the formation of defects at the surface of ALD TiO_2_ strongly reduced the tendency of the Au clusters to agglomerate. A singular value decomposition (SVD) algorithm was applied to analyze a series of MIE spectra and separate them into reference spectra. They found reference spectra that represent i) titania and ii) Au clusters attached to the surface for both Au_9_ and Au_13_ deposited on ALD TiO_2_. An interesting finding was that the reference spectrum for Au_13_ shifted closer to the Fermi level compared to Au_9_. The authors suggested that the increase in the number of atoms forming the Au cluster from 9 to 13 leads to a shift of the electronic states toward the Fermi level.

### 4.4 X‐Ray Absorption Spectroscopy (XAS)

X‐ray absorption spectroscopy (XAS) is a widely used tool for determining the interatomic distance, metal–ligand bond length, and average coordination number that are otherwise impossible to obtain for noncrystalline compounds. Absorption spectra are measured by X‐ray excitation of a core electron to an unoccupied orbital in an atom. X‐ray absorption spectra tend to exhibit a sharp increase in absorption, called an edge. This absorption peak at the edge corresponds to a transition from the core level to the unoccupied valence states of an atom, which are sensitive to the local environment.

XAS is divided into two parts: the lower energy region, known as X‐ray absorption near edge structure (XANES), and the higher energy region, known as extended X‐ray absorption fine structure (EXAFS). XANES has two edges: rising edge (high‐energy edges) and pre‐edge (low‐energy edges). The result of low‐energy edges is usually referred to as near‐edge X‐ray absorption fine structure (NEXAFS). XANES is commonly employed to probe the oxidation states, symmetry, coordination environment, and density of states (DOS) while EXAFS is used to determine local atomic structure including bond length, coordination number, and type of ligands. The versatility of XAS is made possible by simultaneous measurements with other techniques such as UV–visible and infrared spectroscopy, and small‐angle X‐ray scattering. The Au L_3_‐edge is typically used to record EXAFS and XANES spectra in the range of 11 880 and 12 000 eV.

In the absence of single crystals, EXAFS is a powerful tool to establish a plausible structure. This is particularly true for Schmid's Au_55_ cluster where the power sample is amorphous and cannot be grown into single crystals due to decomposition. Early works to investigate the structure and bonding in Schmid's Au_55_ cluster utilized XAS.^[^ ^232^, ^270^ ^]^ A measurement using Au L_3_‐edge EXAFS revealed shorter A‐Au distances (2.76–2.78 Å) in Au_55_(PPh_3_)_12_Cl_6_ than in bulk gold and a mean coordination number of seven, which suggested a cuboctahedral structure.^[^ ^232b^ ^]^ This finding is in contrast with the result from powder X‐ray diffraction (XRD) that assigned an icosahedra structure to the same cluster. A later investigation that combined EXAFS, XANES, and wide‐angle X‐ray scattering (WAXS) revealed a face‐centered cubic structure characteristic of bulk gold.^[^ ^271^ ^]^ Marcus et al. performed temperature‐dependent EXAFS measurements on Au_55_(PPh_3_)_12_Cl_6_ and found that the cluster had 40% less thermal vibration compared to bulk gold due to the stiffening of the Au–Au bonds in the cluster.^[^ ^232a^ ^]^

Menard et al. provided evidence for the structure of highly monodisperse mixed‐ligand Au_13_(PPh_3_)_4_(SR)_2_Cl_2_ and Au_13_(PPh_3_)_4_(SR)_4_ clusters using XAS.^[^ ^272^ ^]^ They attributed the Au_13_ structure to an icosahedral structure with a size of 0.8 nm as observed by STEM. Changes in the EXAFS and XANES spectral features can provide a signature for the structural transformation or evolution of clusters. For example, Li et al. observed an icosahedral‐to‐cuboctahedral structural transformation of Au_13_ clusters for the first time using a solvent‐exchange method.^[^ ^273^ ^]^ The Fourier‐transformed *k* ^2^ *χ*(*k*) function in hexane showed a significant reduction of the Au‐ligand peak amplitude, a reduction in coordination number from 0.9 to 0.4, and a marked increase in the Au–Au peak intensity. These results suggest that changing the solvent from ethanol to hexane leads to rapid thiolate desorption from the Au_13_ core and then rearrangement of the core to a cuboctahedral structure.

An in situ XAS experiment by Kilmartin et al. followed the removal of phosphine ligands from Au_6_(Ph_2_P‐*o*‐tolyl)_6_(NO_3_)_2_ clusters at low temperature by organic hydrogen peroxide.^[^ ^274^ ^]^ It was observed that after the addition of peroxide, the coordination number of Au–P decreased from 1.6 to ≈0.35 and that of Au–Au increased from 3.5 to 8.7, suggesting removal of the ligands and the appearance of metallic gold. The authors concluded that the removal of the ligand had occurred gradually.

Doping of Au clusters with a transition metal atom can change the fluxional nature of Au clusters. For instance, structural isomerization of [Au_9_(PPh_3_)_8_]^3+^ between the crown and the butterfly structures is inhibited by substituting the central Au atom of [Au_9_(PPh_3_)_8_]^3+^ with a single Pd atom to produce a preferred AuPd_8_ crown structure.^[^ ^23^ ^]^ This was demonstrated by analyzing the Debye−Waller factors of the radial and lateral Au−Au(Pd) bonds as a function of temperature using Fourier transformed EXAFS. It was found that the bond strength for both the radial Au–Pd and lateral Au‐–Au bonds in PdAu_8_ are stiffened compared to the Au−Au bonds in Au_9_ due to the central Pd atom doping.

One XAS study by Liu et al. investigated the correlation between the electronic and geometric structure of Au_25_(PPh_3_)_10_(SR)_5_Cl_2_ and Au_25_(SR)_18_ clusters and their catalytic activity.^[^ ^275^ ^]^ The authors found that the d‐bond electrons of the clusters are affected by the variation of the ligands. It was suggested that the differences in the d‐band unoccupied‐state populations are correlated with the differences in catalytic activity and selectivity of these clusters. More recently, the ligand effect on the Au 5d electronic state in [Au_9_(PPh_3_)_8_]^3+^ and [Au_25_(SC_2_H_4_Ph)_18_]^−^ has been reported by Matsuyama et al.^[^ ^276^ ^]^ The authors concluded that the interaction between the unoccupied 5d orbitals with the S/P 3s+3p orbitals lead to different peak positions in the XANES spectra of both clusters; the white‐line peak of Au_9_ is higher by 3 eV than that of Au_25_.

**Figure 11:**
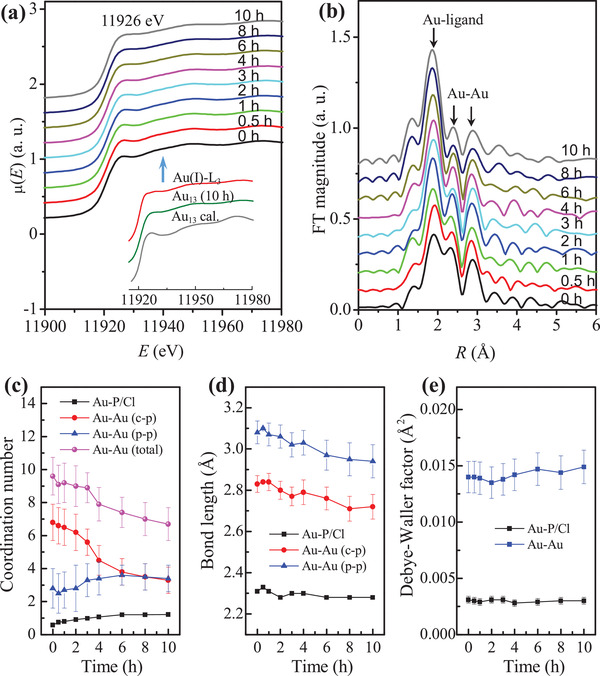
Time‐dependent a) XANES and b) EXAFS spectra, c) coordination number, d) bond distance R, and e) Debye‐Waller factor for the Au−P/Cl and Au−Au coordination pairs extracted from EXAFS curve‐fitting against reaction time. Reproduced with permission.^[^ ^144a^ ^]^ Copyright 2015, Royal Society of Chemistry.

Understanding the etching mechanism during the formation of Au clusters is central for rational design and synthesis of clusters in future. The research group of Wei reported a number of studies using in situ XAS to study the structure and composition of Au clusters during cluster formation.^[^ ^144^, ^277^ ^]^ The formation process of monodisperse Au_13_(L_3_)_4_Cl_4_ with HCl etching of a polydisperse mixture was traced using in situ XAS. The XANES spectra in **Figure** **11**a show the white line peak at 11926 eV, which emerges from the excitation of Au 2p_3/2_ electrons to the unoccupied Au 5d state, is strengthened immediately after addition of HCl (0 to 0.5 h). This is assumed to be due to the charge transfer from Au atoms to the Cl^−^ ligands. Figure 11b shows EXAFS spectra with the Au‐ligand peak at 1.90 Å intensified and the Au–Au peaks at 2.36 and 2.88 Å decreased after the addition of HCl. This indicates the decomposition of the larger Au clusters into smaller intermediates. The changes in the XANES and EXAFS spectra continued over the reaction time but not as markedly as the first hour. The same group showed the formation process of monodisperse Au_13_(L_3_)_2_(SR)_4_Cl_4_ (Au_13_) mixed‐ligand clusters,^[^ ^144b^ ^]^ observing that the formation of Au_13_ clusters occurred in three steps: etching, growth, and rearrangement.

Several studies have used XAS to determine the change in size of supported clusters after removal of the ligands.^[^ ^15^, ^248^, ^267^ ^]^ It has been demonstrated that the properties of the support play a crucial role in the stability of surface‐supported gold phosphine clusters. Donoeva and co‐workers demonstrated that thermal treatment of Au_9_ clusters on Brønsted acidic supports such as carbon and SiO_2_ leads to fragmentation into isolated Au‐ligand species as manifested by the absence of an Au–Au bond in the EXAFS spectra.^[^ ^28d^ ^]^ In contrast, phosphine ligand migration from Au_9_ clusters to CeO_2_ was signified by the absence of an Au–P peak, which also resulted in the formation of an active catalyst.

### 4.5 Mass Spectrometry

In the absence of crystal structures by single‐crystal X‐ray diffraction (XRD), mass spectrometry (MS) becomes an indispensable and powerful tool to determine molecular formula, composition, core size nuclearity, charge state, and structural information of gold clusters. Advances in MS with improved instrumentation, ionization, sensitivity, and resolution have enabled the acquisition of high‐resolution mass spectra. The ionization technique in MS is key to determining what samples can be analyzed. Soft‐ionization techniques such as fast atom bombardment (FAB),^[^ ^278^ ^]^ matrix‐assisted laser desorption/ionization (MALDI), and electrospray ionization (ESI) are commonly used in mass spectrometric analysis of atomically precise gold clusters. ESI‐MS is more prevalent nowadays and has the advantages of producing little fragmentation and being able to be coupled to other techniques such as ion mobility separation, capillary electrophoresis, collision‐ and surface‐induced dissociations.

The generic formula [Au_n_L_s_X_m_]*^q+^* of gold–phosphine clusters renders suitability of analysis by mass spectrometry and the technique has confirmed the exact composition and molecular formula of many gold clusters. Gold–phosphine clusters often carry a positive charge with a definite number of counterions such as NO_3_^−^, Cl^−^, Br^−^, PF_6_^−^ and BF_4_^−^. This intrinsic charge allows straightforward and sensitive determination by ESI‐MS in positive‐ion mode. In a mass spectrum, peaks are shown as a mass‐to‐charge (m/z) ratio. For two or more clusters with the same m/z ratio (e.g., the peaks for both [Au_6_(PPh_3_)_6_]^2+^ and [Au_3_(PPh_3_)_3_]^+^ appear at 1377.77), isotopic patterns can distinguish between the two species.

Identifying initial species and intermediates at the early stage of synthesis is key to understanding nucleation, growth mechanism, and selective formation of Au clusters. These species determine the growth pathway, size distribution, and relative yield of the final clusters. Nonetheless, a complete knowledge of the early stages is obscured owing to inherent complexities such as high reduction rate, large range of intermediates, and solution equilibrium. Over 100 different clusters were found in the range of 250–4000 m/z in the synthesis of PPh_3_‐ligated Au clusters by Hewitt et al.^[^ ^279^ ^]^ Using mild reducing agents and/or diphosphine ligands to slow down the reaction kinetics, several initial species including Au(PPh_3_)_2_^+^, Au(PPh_3_)L^+^, AuL_2_^+^ and Au_2_L_2_^2+^, chlorinated and oxygenated complexes were observed in ESI‐MS data of a Au(PPh_3_)Cl:L (L = diphosphine) mixture prior to reduction.^[^ ^27^, ^280^ ^]^

**Figure 12:**
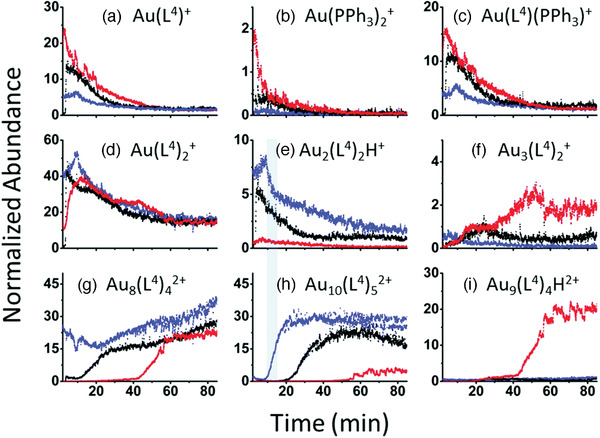
Ion chronograms of intermediates and product clusters under nonoxidative conditions; blue curve: ratio BTBA:Au(PPh_3_)Cl at 50:1, black curve: ratio of BTBA:Au(PPh_3_)Cl at 25:1, and red curve: ratio of BTBA:Au(PPh_3_)Cl at 8;1. BTBA and Au(PPh_3_)Cl are borane *tert*‐butyl amine (reducing agent) and the gold precursor, respectively. Reproduced with permission.^[^ ^26d^ ^]^ Copyright 2017, Royal Society of Chemistry.

Real‐time monitoring offers a means to correlate the initial species with the corresponding final clusters. For example, using a combination of UV–visible and mass spectra recorded prior to reduction and after 14 days, Pettibone and Hudgens associated the initial complexes [Au_2_(L^6^)_2_]^2+^, Au(PPh_3_)^2+^ and chlorinated complexes, and reduced chlorinated digold to the formation of octagold [Au_8_(L^6^)_4_]^2+^, nonagold [Au_9_(L^6^)_4_]^2+^ and decagold [Au_10_(L^6^)*_x_*]^2+^ (*x* = 4, 5), respectively.^[^ ^27a^ ^]^ In other work by Ligare et al., time‐dependent studies of ESI‐MS revealed that a significant degradation rate of Au_2_(L^4^)_2_H^+^ was concurrent with the growth rate of even‐numbered clusters, [Au_8_(L^4^)_4_]^2+^ and [Au_10_(L^4^)_5_]^2+^.^[^ ^26d^ ^]^ Meanwhile, the formation of [Au_9_(L^4^)_3_H]^2+^ was ascribed to two possible pathways: i) odd‐numbered [Au_3_(L^4^)_2_]^+^ and [Au_6_(L^4^)_3_]^2+^ intermediates, and ii) chlorinated Au_9_ and Au_11_ intermediates that decay in abundance coinciding with the growth of [Au_9_(L^4^)_3_H]^2+^, as evidenced in the ion chronograms (see **Figure** **12**). Furthermore, cluster transformation can be probed by mass spectrometry by identifying short‐lived and metastable species to provide understanding of cluster reactivity, stability, and identifying degradation pathways.^[^ ^53^ ^]^ Such knowledge lays the foundation to afford size‐specific Au clusters by understanding kinetic control of the initial complexes and intermediates.

Detection of hydrogen in mass spectrometry is indispensable because it cannot be located and identified by single‐crystal XRD and XPS due to the lack of scattering power and lack of core electrons, respectively. Small hydride‐containing intermediates were detected in ESI mass spectra as building blocks for formation of larger clusters.^[^ ^281^ ^]^ The inclusion of hydrogen from the reductant is verified by the mass spectra of deuterated‐containing species obtained using NaBD_4_. The high sensitivity and resolution of mass spectrometry allow distinction by 1 Da in the mass spectra of deuterium‐adduct clusters; for example [PdAu_10_D(PPh_3_)_8_Cl_2_]^2+^ cf. [PdAu_10_H(PPh_3_)_8_Cl_2_]^2+^.^[^ ^56^, ^121^ ^]^

Fragmentation discloses a wealth of information including thermochemical data, size‐dependent reactivity, stability of parent and fragment ions, short‐lived intermediates, mechanism of fragmentation, and surface bonding. Gas‐phase fragmentation studies are of critical importance because it presents the true nature of clusters without any influence from solvents and can be compared to theoretical calculations. Advantageously, ESI‐MS can be easily coupled to collision‐induced dissociation (CID) and surface‐induced dissociation (SID) instruments. In dissociation studies, the cluster ions of interest are mass‐selected and subjected to the collision to produce fragment ions. By analyzing the relative abundance of fragment ions, it is possible to deduce fragmentation channels and kinetics, reactivity, ligand stability, and binding energy of Au clusters.^[^ ^282^ ^]^ Fragmentation channels exhibited by gold–phosphine clusters are (asymmetric or symmetric) core fission, neutral ligand loss, and ligand activation. The fragments tend to favor even‐numbered electron ions consistent with a closed‐shell electronic configuration.

Collision‐induced dissociation (CID) mass spectrometry is a powerful tool to qualitatively investigate the relative binding energy of ligands, reactivity, and bonding nature of ligated Au clusters. Upon collision with neutral background gas (Ar, N_2_, He etc.), ions undergo unimolecular fragmentation. Importantly, fragmentation is not a random process but is determined by a set of factors such as electronic stability, number of Au atoms, and intact ligands. Such processes also permit synthesis of new gas‐phase cluster ions via fragmentation which is otherwise impossible to achieve in solution‐phase synthesis.^[^ ^283^ ^]^ For example, the CID spectra of disphosphine‐ligated octa‐, deca‐ and undecagold revealed the presence of [Au_3_L]^+^, which may serve as a building block for large clusters/nanoparticles, even though it has not been chemically prepared and isolated in solution.^[^ ^284^ ^]^

Tsukuda and co‐workers discovered that the steric influence of ligands (e.g., Cl vs C≡CPh) leads to different branching fragmentation ratios of two undecagold species, [Au_11_(PPh_3_)_8_(C≡CPh)_2_]^2+^ and [Au_11_(PPh_3_)_8_Cl_2_]^2+^.^[^ ^285^ ^]^ Dissociation of AuPPh_3_C≡CPh is inhibited in the former due to the bulkier ligand while a competitive loss of Au(PPh_3_)Cl and PPh_3_ occurs in the latter. Importantly, Johnson et al. successfully established the relative ligand binding energy of substituted monophosphines in CID experiments in the order PMe_3_ < PPhMe_2_ < PPh_2_Me < PPh_3_ < PPh_2_Cy < PPhCy_2_ < PCy_3_.^[^ ^26b^ ^]^ By measuring the rate of loss of neutral ligands in Au_8_(PPh_3_)_7‐_ *_n_* L*_n_* ^2+^ and Au_10_(PPh_3_)_8‐_ *_n_* L*_n_* ^2+^ clusters (where L = substituted monophosphines), it was found that cluster size, charge state, number of exchanged ligands, and number of substituted groups influence the ligand binding energy. This finding is crucial when choosing suitable ligands for the rational design of size‐specific Au clusters.^[^ ^38^ ^]^

Surface‐induced dissociation (SID) involves directing parent ions to collide with a massive flat surface, usually gold functionalized with self‐assembled alkanethiols, that can impart a large amount of internal energy into the ion. Compared to CID, SID allows better control of energy by depositing a narrower distribution of internal energies into the parent ions. This process has an efficient kinetic to internal energy conversion and enables collision energy‐dependent fragmentation, thus providing a useful way to extract the threshold energy and activation entropy. Additionally, the delay time in SID can be used to measure the fragmentation kinetics. While CID is restricted to qualitative data, fitting the SID data using Rice‐Ramsperger‐Kassel‐Marcus (RRKM) theory can provide quantitative thermodynamic and kinetic dissociation data that can be compared with state‐of‐the‐art theoretical calculations.

A useful feature of SID spectra is fragmentation curves, i.e., plots of normalized abundance in terms of collision energy, or collision energy per vibrational degrees of freedom, which is governed by the energetics and dynamics of dissociation. The relative position of this curve reflects the stability of clusters. For example, the survival curves of Au_7_(PPh_3_)_6_^2+^, Au_8_(PPh_3_)_7_^2+^, and Au_9_(PPh_3_)_7_^2+^ overlap exactly implying similar overall stability toward dissociation.^[^ ^286^ ^]^ In contrast, Au_8_(PPh_3_)_6_^2+^ exhibits markedly higher stability as indicated by the position of the survival curve at higher collision energy. Importantly, the SID experiments demonstrated that the stability of Au_8_(PPh_3_)_6_^2+^, Au_8_(PPh_3_)_7_^2+^, and Au_9_(PPh_3_)_7_^2+^ is largely determined by the neutral ligand binding energy while Au_7_(PPh_3_)_6_^2+^ is characterized by three competitive fragmentation channels: neutral ligand loss, asymmetric and symmetric core fissions.

Further useful information that can be extracted from SID experiments is activation entropy. Activation entropy provides information about the kinetics of dissociation/fragmentation in mass spectrometry. This activation entropy can be determined from RRKM modeling of the SID data. For the same charge state (+2) in PPh_3_‐ligated small Au clusters, the experimental threshold energies for dissociation of a PPh_3_ ligand from Au_7_(PPh_3_)_6_^2+^, Au_8_(PPh_3_)_7_^2+^, and Au_9_(PPh_3_)_7_^2+^ were determined to be 1.58, 1.44 and 1.53 eV, respectively, with corresponding activation entropies of 84, 90, and 93 cal mol^‐1^ K^‐1^, respectively.^[^ ^286^ ^]^ However, a substantially larger ligand dissociation energy (1.78 eV) was obtained for the more stable Au_8_(PPh_3_)_6_^2+^, consistent with its initial high abundance during synthesis and stability against fragmentation. The SID experiments also revealed that despite the low threshold energy for core fission, that the low activation entropy renders this fragmentation channel kinetically hindered for all clusters studied. For Au_7_(PPh_3_)_6_^2+^, a competition between neutral ligand loss and kinetically favored core fragmentation results in reduced overall stability. Interestingly, the loss of neutral Au(PPh_3_) from Au_9_(PPh_3_)_7_^2+^, characterized by low fragmentation energy (1.36 eV) to form minor yet stable Au_8_(PPh_3_)_6_^2+^, suggests that the reverse addition might also occur during the synthesis to yield Au_9_(PPh_3_)_7_^2+^.

Complexity in assessing the stability of ligated metal clusters is not only limited to ligand–core bonding, steric effects, core geometry, and charge transfer but also to ligand–ligand interaction, particularly *π*–*π* and CH–*π* interactions between phenyl (Ph) groups in adjacent PPh_3_ ligands.^[^ ^287^ ^]^ Importantly, each gold–ligand site in a cluster is energetically distinct. SID experiments on ligand‐exchanged Au clusters provide a means to study the effect on the stability of intra‐ and interligands in mixed ligand compositions. Recent work by Ligare et al. found a significant increase of the ligand dissociation energy in mixed PPh_3_/MePPh_2_‐ligated Au_8_ clusters, indicating higher stability than the fully PPh_3_‐ligated octagold cluster.^[^ ^288^ ^]^ Such large dissociation energies (1.4–2.2 eV) are counterintuitive because PPh_3_ has stronger bonding to the Au core and by the fact that mixed‐ligand octagold is never observed in a synthesis. The large binding energies are attributed to the electron‐donating ability of the two competing (PPh_3_ and MePPh_2_) ligands and increased van der Waals ligand–ligand interaction, where a weaker *π*–*π* interaction is converted into a stronger CH–*π* interaction.

Of importance, the binding energy parallels the activation entropy for neutral ligand dissociation. The fast PPh_3_ and MePPh_2_ dissociation from mixed‐ligand Au_8_ clusters are kinetically favored as manifested by the large activation entropy (50–170 cal mol^‐1^ K^‐1^).^[^ ^288^ ^]^ Major contributions to the activation entropy are from breaking the interligand interaction to release restricted modes and core charge distribution after ligand exchange, whereas many transition‐state structures contribute minimally to the entropy. Such findings demonstrate that the reactivity of octagold clusters in ligand exchange reactions is entropically driven at room temperature in spite of large ligand dissociation energy. The nature of ligands including steric effects, van der Waals interligand interaction, and electron‐donating ability, as well as core charge redistribution upon ligand exchange dominate the reactivity and stability of Au clusters.

Ion‐mobility mass spectrometry (IM‐MS) can disclose structural information including differing isomers of isolated ions, isomeric transformation, and structural dynamics of clusters in the gas phase.^[^ ^289^ ^]^ It separates gas‐phase ions depending on their mobility through a buffer gas. Therefore, mass‐selected ions having the same composition, yet different size, shape, and conformation display different collision cross‐sections (CCSs) and have different arrival time distributions (ATDs). Compact structures travel faster because of less interaction with the buffer gas than extended or disordered structures and thus display small ATDs. Structural elucidation is then made by comparing the experimental CCS values from ion‐mobility experiments with theoretically calculated ones. However the measurement and determination of CCS values are complicated by size, structure, charge state and charge distribution of gas ions, and electronic properties of the buffer gas.^[^ ^290^ ^]^

Ligare et al. determined the structure of a hydrogen‐containing metastable intermediate, Au_7_(PPh_3_)_7_H_5_^2+^_,_ using IM‐MS combined with DFT calculations. The proposed structure consists of three bridging Au–H–Au and two single‐bonded Au–H motifs.^[^ ^291^ ^]^ The experimental CCS value (421) matches spectacularly well with the theoretically derived value (422). By comparing the CCS values, it is feasible to identify the presence of different isomers/structures and track the structural transformation within the IM‐MS experiment. Isomeric transformation induced by ligand exchange in Au clusters has also been reported and verified by IM‐MS. It was observed that gradual ligand exchange of PPh_3_ with MePPh_2_ in Au_8_(PPh_3_)_7_^2+^ resulted in more extended structures due to the lower binding energy and steric hindrance of MePPh_2_ as indicated by the relative population of both structures in the arrival time spectra.^[^ ^26c^ ^]^ A plot of ATDs per ligand exchange shows similar slopes for the compact (−287 ± 32 µs per ligand) and extended (‐315 ± 32 µs per ligand) structures and thus the isomerization dynamics of Au_8_(PPh_3_)_7‐_ *_x_* (MePPh_2_)*_x_* (*x* = 1–3) can be attributed to ligand exchange.

**Figure 13:**
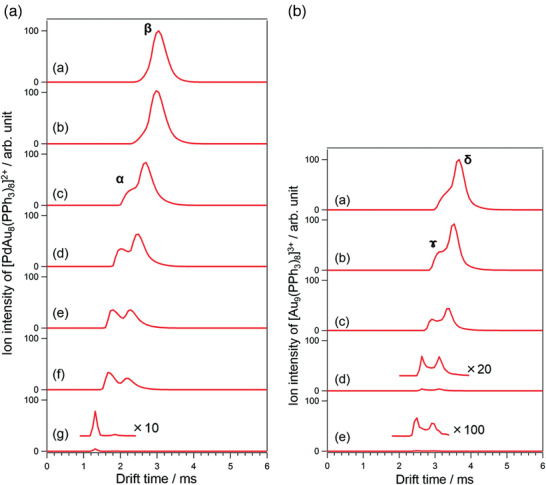
Arrival time distributions (ATDs) of a) [PdAu_8_(PPh_3_)_8_]^2+^ and b) [Au_9_(PPh_3_)_8_]^3+^ at fixed N_2_ flow rates and decreasing He flow rates from 150 a) to 0 mL min^−1^ g) and 80 a) to 35 mL min^−1^ e), respectively. Reproduced with permission.^[^ ^293^ ^]^ Copyright 2018, American Chemical Society.

The collision of ions with buffer gases (He, N_2_ etc) may impinge on structural transformation via momentum transfer.^[^ ^292^ ^]^ Using the oblate‐shape clusters Au_9_(PPh_3_)_8_]_2+_ and [PdAu_8_(PPh_3_)_8_]^2+^, Tsukuda and co‐workers demonstrated structural isomerization from a disordered to a packed ligand shell induced by collisional activation and cooling.^[^ ^293^ ^]^ **Figure** **13** shows that disordered structures (labeled *β* and *δ*) are converted into compact structures (labeled *α* and *τ*) with shorter ATDs as the He flow rate is reduced, thus indicating higher collisional energy with N_2_. This work suggests that the disordered ligand packing in solution is retained after ionization in the ESI source shortly before collision and is converted into a compact, more stable structure upon collision with N_2_ in the gas phase, which is similar to the crystallized structure. Such isomerization changes are consistent with the soft‐potential energy surfaces of Au clusters and may shed light on the effects of fluxionality‐dependent catalytic activity in gas‐phase catalysis.

The reactivity and stability of clusters on surfaces play a key role in catalysts, sensors, and electrodes. Ion soft landing (SL) is a technique that involves deposition of mass‐selected clusters onto conductive, semiconductive, or self‐assembled monolayer surfaces with controlled coverage and kinetic energy. Using this technique, deposition of high purity (without contamination from solvent) and monodisperse (size‐ and charge‐selective) ligated metal clusters can be achieved. Thus, SL is a precise approach to study the structure–property relationships such as structural transformation, charge retention, and reactivity as a function of size, as well as the interaction between clusters and surfaces. The Laskin group successfully demonstrated deposition of monodisperse Au_11_(dppp)_5_^3+^ cluster ions at high coverage on self‐assembled monolayers (SAM) on gold.^[^ ^294^ ^]^ It was found that the 3+ charge state was retained on perfluorodecanethiol (FSAM) while an instantaneous reduction to 2+ and 1+ charge states occurred on mercaptohexadecanoic acid (COOH‐SAM) and 1‐dodecanethiol (HSAM) surfaces.^[^ ^295^ ^]^ At higher coverage (≥10^12^ Au_11_(dppp)_5_^3+^ clusters distributed over a 5 mm diameter spot), a significant charge reduction was observed on FSAM.^[^ ^296^ ^]^ Such findings provide insight into selecting appropriate SAM surfaces and cluster coverage for preparing highly monodisperse samples. It was also demonstrated that a smaller cluster, Au_10_(dppp)_4_^2+^, was far more reactive compared to Au_11_(dppp)_5_^3+^ since it formed a species containing an additional Au atom on the FSAM and HSAM surfaces.^[^ ^297^ ^]^

### 4.6 Nuclear Magnetic Resonance (NMR)

Nuclear magnetic resonance (NMR) has been a pivotal tool for the structural elucidation of organic compounds over many decades. Additionally, NMR spectroscopy enables the probing of surface interactions and interfacial chemistry between ligands and a metal surface.^[^ ^298^ ^]^ However, its use in ligand‐protected metal clusters is not as prevalent as X‐ray crystallography. Many metal nuclei have low sensitivity toward magnetic fields due to their low spin‐active abundance. Commonly used nuclei (^1^H, ^13^C, ^17^O, ^31^P) are beneficial to probe the nature and dynamics of the stabilizing ligands attached to the surface of nanoparticles/clusters. In the case of phosphine‐ligated Au clusters, proton decoupled ^31^P{^1^H} NMR studies unveil valuable information about the structure, chemical bonding, dynamic properties, fluxionality, and surface chemistry of the ligands.

First, ^31^P NMR spectroscopy is an indispensable tool to study the fluxionality of gold–phosphine clusters. In general, a single, averaged resonance is observed in the ^31^P NMR solution spectra for many monophosphine‐ligated Au clusters at room temperature due to dynamic equilibration of nonequivalent phosphorous sites by rapid intermolecular exchange processes.^[^ ^20^, ^299^ ^]^ Such an effect gives rise to fluxionality in solution. While many gold–phosphine clusters display fluxionality down to low temperature, some clusters show rigidity at low temperature as evidenced in their low‐temperature ^31^P NMR spectrum. For example, the 2D homonuclear correlational spectroscopy (COSY) ^31^P NMR spectrum of [Au_8_(PPh_3_)_8_]^2+^ shows four resonances at 203 K, corresponding to four distinct Au–P sites instead of a single resonance at room temperature.^[^ ^300^ ^]^

Second, ^31^P NMR spectroscopy facilitates identification of Au clusters because phosphine ligands are influenced by the geometry/structure and composition of Au clusters.^[^ ^301^ ^]^ Different peak positions are associated with different clusters and thus ^31^P NMR spectra become a fingerprint for gold–phosphine clusters. **Table** **4** summarizes the ^31^P peak shift of several phosphine‐ligated Au clusters. Importantly, ^31^P NMR spectroscopy is useful to study the formation and to assess the purity of synthesized Au clusters.^[^ ^302^ ^]^ For example, Velden and co‐workers employed ^31^P NMR to probe the evolution of [Au_8_(PPh_3_)_8_]^2+^ from the starting material [Au_9_(PPh_3_)_8_]^3+^ upon addition of excess PPh_3_, which revealed the intermediate [Au_8_(PPh_3_)_7_]^2+^ and by‐product [Au(PPh_3_)_2_]^+^.^[^ ^11a^ ^] 1^H NMR spectroscopy has also been employed to distinguish different gold–phosphine clusters although it is less informative compared to ^31^P NMR.^[^ ^67^, ^303^ ^]^

**Table 4:** ^31^P{^1^H} NMR peaks for different Au clusters

Compound or cluster	*δ* [ppm]	Solvent	Refs.
AuPPh_3_Cl	33.5	CDCl_3_	[15a]
AuPPh_3_NO_3_	25.2	CDCl_3_	[15a]
Au_4_(PPh_3_)_4_I_2_	47.4	CD_2_Cl_2_	[6h]
Au_5_Cu_6_(dppf)_2_(SR)_6_BPh_4_	45.12	CD_2_Cl_2_	[45]
Au_6_(PPh_3_)_6_(NO_3_)_2_	53.3	CH_2_Cl_2_	[6, 180]
Au_8_(PPh_3_)_8_(NO_3_)_2_	55.0	CD_2_Cl_2_	[25c]
Au_9_(PPh_3_)_8_(NO_3_)_3_	56.9	CD_2_Cl_2_	[25a]
[Au_9_H(PPh_3_)_8_]^2+^	53.5	CD_2_Cl_2_	[53]
Au_11_(PPh_3_)_7_Br_3_	52.8	CD_2_Cl_2_	[162]
Au_11_(PPh_3_)_7_Cl_3_	52.9	CD_2_Cl_2_	[67]
[Au_11_(PPh_3_)_8_Cl_2_]Cl	52.2	CD_2_Cl_2_	[67]
Au_6_(dppp)_4_(NO_3_)_2_	60.24, 51.07	CD_2_Cl_2_	[6h]
Au_6_(dppp)_4_(BPh_4_)_2_	62.1, 55.5	(CD_3_)_2_CO	[131a]
Au_6_(dppb)_4_(BPh_4_)_2_	55.1, 51.4	(CD_3_)_2_CO	[131a]
Au_8_(dppp)_4_Cl_2_(PF_6_)_2_	55.4, 51.7, 33.5	CD_2_Cl_2_	[51]
Au_8_(dppp)_4_(NO_3_)_2_	62.0, 58.0	CD_2_Cl_2_	[51]
Au_10_(PPh_3_)_7_[S_2_C_2_(CN)_2_]_2_	47.9	n/a	[63]
Au_11_(dppe)_6_(SbF_6_)_3_	ca. 49, 52.7, 60.5	CD_2_Cl_2_	[74]
[Au_11_(DPEphos)_4_Cl_2_]Cl	47.75, 50.96, 52.67,	CDCl_3_	[72]
[Au_11_(Xantphos)_4_Cl_2_]Cl	49.26 (d), 54.76 (d), 57.78, 59.48	CDCl_3_	[72]
[Au_13_(PPhMe_2_)_10_Cl_2_](PF_6_)_3_	39.0, 36.8, 36.4, 24.7	CD_2_Cl_2_	[38]
[Au_13_(dppe)_5_Cl_2_]Cl_3_	67.2	CD_3_OD	[80]
E‐[Au_13_Ag_12_(PPh_3_)_10_Cl_8_]SbF_6_	54.80	CD_2_Cl_2_	[84]
S‐[Au_13_Ag_12_(PPh_3_)_10_Cl_8_]SbF_6_	57.36	CD_2_Cl_2_	[84]
[Au_19_(C≡CPh)_9_(Hdppa)_3_](SbF_6_)_2_	76.44	CD_2_Cl_2_	[88]
[Au_20_(PPhpy_2_)_10_Cl_4_]Cl_2_	52.15	CD_2_Cl_2_	[92]
Au_20_(PP_3_)_4_Cl_4_	66.26 (d), 57.05, 48.56 (q), 41.32, 39.78	CD_2_Cl_2_	[304]
[Au_20_(PPh_3_)_12_H_3_](SbF_6_)_3_	56.56	CD_2_Cl_2_	[20b]
[Au_23_(PPh_3_)_6_(C≡CPh)_9_](SbF_6_)_2_	49.17	CD_2_Cl_2_	[97]
[Au_24_(PPh_3_)_4_(C≡CPh)_14_](SbF_6_)_2_	40.22	CD_2_Cl_2_	[98]
[Au_24_(dppb)_6_Cl_4_]Cl_2_	87.3, 83.7, 78.0	CD_3_OD	[99]
[Au_32_(PPh_3_)_8_(dpa)_6_](SbF_6_)_2_	29.50	CD_2_Cl_2_	[107]
Au_54_(PEt_3_)_18_Cl_12_	96.93	CD_2_Cl_2_	[37]

Third, ligand dynamics including exchange processes and intramolecular rearrangement can be studied using both ^1^H and ^31^P NMR spectroscopy.^[^ ^156a^ ^]^ For example, Sharma et al. revealed that ligand exchange in 1.8 nm PPh_3_‐ligated Au nanoparticles displaced Au(PPh_3_)Cl complexes from the nanoparticle surface upon addition of d_15_‐PPh_3_ or Au(d_15_‐PPh_3_)Cl.^[^ ^305^ ^]^ In their work, surface‐bound PPh_3_ shows a broad resonance in the ^1^H and ^31^P spectra due to heterogeneous chemical environments on the surface. Solid‐state ^2^H NMR has also been employed to study intramolecular dynamic processes of surface‐bound ligands. Fast *π* flips of phenyl rings (*k* ≥ 7 × 10^7^ s^−1^) has been observed to occur only in PPh_3_‐capped Au nanoparticles due to well‐separated ligands on the nanoparticle surface, which is in contrast to the Au(PPh_3_)Cl complex.^[^ ^306^ ^]^

Large gold–phosphine nanoparticles display a broad resonance due to incomplete averaging of the surface‐bound PPh_3_ in different chemical environments. However, Marbella et al. recently reported that 1.8 nm PPh_3_‐capped Au nanoparticles showed a sharp resonance in the ^31^P NMR solution spectrum.^[^ ^307^ ^]^ Using ^1^H‐^31^P cross‐polarization magic‐angle spinning (MAS) solid‐state NMR, the authors attributed the observed sharp resonance to ^31^P−^197^Au coupling.

### 4.7 Infrared and Far‐Infrared Spectroscopy

Infrared (IR) spectroscopy is a key tool for studying vibrational transitions and for identification of functional groups of molecules. For example, changes in chemical bonding of ligands (i.e., free ligand vs chemically bonded ligand to the metal cores) have been detected using IR spectroscopy.^[^ ^308^ ^]^ For IR allowed transitions, a molecule/cluster must have a change in dipole moment upon IR light irradiation and because vibrational transitions are governed by quantum mechanical selection rules, the transitions probed by IR spectroscopy reflect the symmetry and thus the structure of metal clusters. Despite significant attention and progress in atomically precise metal clusters, detailed IR studies and vibrational transitions are difficult to find in the literature. Early reports on IR studies of atomically precise phosphine‐ligated gold clusters assigned vibrations due to PPh_3_ ligands and NO_3_^−^ counter ions.^[^ ^6^, ^57^ ^]^ Cariati and Naldina employed IR spectroscopy to verify the presence of covalent bonds between coordinating ligands such cyanide (CN^−^) or thiocyanide (SCN^−^) and Au atoms.^[^ ^309^ ^]^

Owing to the exact number of atoms and well‐defined structure, atomically precise Au clusters offer an opportunity to study metal–ligand and metal–core bonding. Au core vibrations in clusters are expected to occur at low frequency with very weak intensity.^[^ ^310^ ^]^ Such vibrations lie in the far region of the IR spectrum between 50 to 500 cm^−1^. Thus, far‐IR spectroscopy is a powerful and critical tool for studying metal core vibrations even though it is relatively practically unexplored. The earliest account of the use of far‐IR spectroscopy to study vibrational transitions in metal clusters was reported by Lever and Ramaswany.^[^ ^311^ ^]^ The authors probed metal–ligand vibrations in binuclear and polymeric compounds which occur below 400 cm^−1^. Adam and Taylor studied vibrational transitions in metal carbonyl clusters, i.e., M_3_(CO)_12_ (M = Ru, Os) using both far‐IR and Raman spectroscopy.^[^ ^312^ ^]^ Dolamic et al. presented far‐IR spectra of thiolate‐protected Au clusters (Au_144_, Au_40_, Au_38_ and Au_25_) and assigned the spectra to different staple units.^[^ ^310^ ^]^ Nonetheless, none of these earlier works focused on metal core vibrations in metal clusters. The first difficulty stems from the low intensity, low energy of metal core vibrations below 250 cm^−1^. Second, it is difficult to obtain high‐quality far‐IR spectra across a wide range of frequencies (wavenumbers).

**Figure 14:**
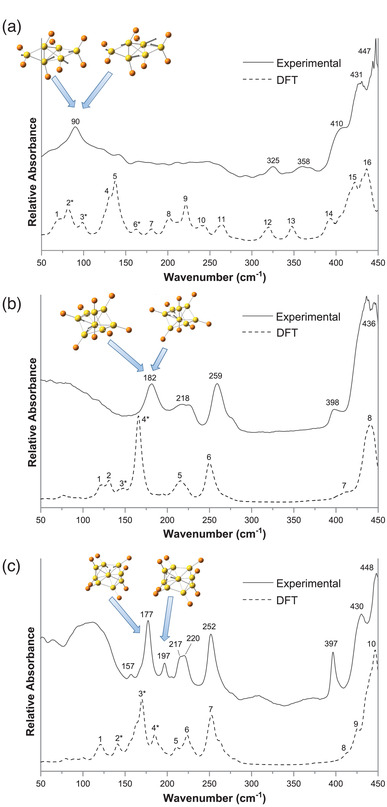
Experimental and DFT‐simulated far‐IR spectra of a) Au_6_(dppp)_4_(NO_3_)_2_, b) Au_8_(PPh_3_)_8_(NO_3_)_2_, and c) Au_9_(PPh_3_)_8_(NO_3_)_3_ clusters. Adapted with permission.^[^ ^313^ ^]^ Copyright 2013, Royal Society of Chemistry.

Alvino et al. investigated vibrational modes of a series of chemically synthesized atomically precise gold–phosphine clusters (Au_6_, Au_6_Pd, Au_8_ and Au_9_) using synchrotron far‐IR spectroscopy.^[^ ^313^ ^]^ By comparing the experimental far‐IR spectra with DFT‐simulated spectra calculated for fundamental vibrations only (excluding overtones and combination bands), the authors were able to unambiguously assign specific vibrational modes, including Au–Au core vibrations in the clusters. **Figure** **14** shows the experimental (top) and DFT‐simulated (bottom) far‐IR spectra of Au_6_(dppp)_4_(NO_3_)_2_ (referred to Au_6_), Au_8_(PPh_3_)_8_(NO_3_)_2_ (Au_8_), and Au_9_(PPh_3_)_8_(NO_3_)_3_ (Au_9_) clusters together with the most intense metal core vibrations.^[^ ^313^ ^]^ A very low and broad peak at 90 cm^−1^ in Figure 14a is attributed to a group of Au core distortions in the Au_6_ cluster. In the Au_8_ cluster, Au core distortions are predicted to occur at 166 cm^−1^ with the highest intensity, and this is assigned to the experimental peak at 182 cm^−1^ in Figure 14b. For the Au_9_ cluster, two sharp, distinct peaks at 177 and 197 cm^−1^ in the experimental spectrum in Figure 14c are attributed to the Au core distortions which match closely to the DFT‐simulated peaks at 170 and 185 cm^−1^. The DFT simulated spectra display a general trend towards longer bonds because the optimized geometry used for calculations was performed in the gas phase while the experimental spectra were recorded in the solid state where lattice packing and *π*–*π* interactions (from PPh_3_ ligands) result in minor differences.

Substitution or/and addition of heteroatoms to metal clusters results in change of geometry, composition, and bonding, thus affecting vibrational transitions. Mixed‐metal Au clusters offer a fascinating opportunity to study fundamental vibrational modes. The far IR spectrum of PdAu_6_(PPh_3_)_7_(NO_3_)_2_ displays three metal core vibrations at 174.5, 197.7 and 218.9 cm^−1^.^[^ ^313^ ^]^ The DFT calculations confirmed that significant motions of Au atoms around the central Pd atom contribute to the core vibrations. Bennett et al. presented a comprehensive study of vibrational modes in Ru_3_(µ‐AuPPh_3_)(µ‐Cl)(CO)_10_ (referred to as AuRu_3_).^[^ ^251a^ ^]^ Two metal core vibrational modes were identified at 177 (theoretical 165 cm^−1^) and 299 cm^−1^ (theoretical 285 cm^−1^) corresponding to the Cl‐Ru_3_‐Au distortion and RuCl_2_ stretching, respectively. The force constants for the distortion and stretching were calculated to be 0.37 and 1.65 mdyne Å^‐1^, respectively.

The structure of atomically precise chemically synthesized ligated Au clusters can be constructed from single‐crystal XRD provided the clusters can be grown into sufficiently large crystals. In contrast, for Au clusters generated in the gas phase without ligands, it is impossible to establish the structure from single‐crystal XRD. Fortunately, far‐IR spectroscopy combined with DFT calculations offers the possibility of structure elucidation of metal clusters in the gas phase.^[^ ^314^ ^]^ Fielicke and co‐workers have studied the structure of several of Au clusters in the gas phase using far‐IR multiple‐photon dissociation (FIR‐MPD).^[^ ^315^ ^]^ Briefly, clusters are prepared by laser ablation with the molecular formula analyzed by time‐of‐flight mass spectrometry, and the far‐IR light is delivered by Free Electron Laser for Infrared eXperiments (FELIX). The authors compared the experimental far‐IR and DFT‐simulated spectra to determine the plausible structure and symmetry of Au clusters. The structures of neutral Au_19_ and Au_20_ clusters were determined to be tetrahedral with *T*_d_ symmetry and truncated trigonal pyramidal with *C*_3_ *_v_* symmetry, respectively.

## 5 Quantum Chemical Calculations

As early as the 1990s, there have been attempts to model phosphine‐stabilized Au clusters.^[^ ^316^ ^]^ A restricted MP2 calculation of a simplified Au_2_(PH_3_)_2_ system showed the importance of relativistic effects in the energetic calculations of Au clusters.^[^ ^316^, ^317^ ^]^ With the continuous development of density functional theory, this formalism became the established method for ground‐state property calculations of Au clusters. Since then, there have been very few studies that have employed the wavefunction theory due to the computational expense, despite the development of high‐performance computers.^[^ ^206^ ^]^

To maintain progress in modeling atomically precise Au clusters, improved computational studies are required. Crucially, joint experimental and computational studies provide important physical insights into observed properties and behaviors. The stability, geometric and electronic structures, charge transfer, ligand effects, reaction mechanisms, and selective formation of gold–phosphine clusters can be computed using density functional theory. This section discusses the key findings to date for quantum chemical calculations on gold–phosphine clusters.

### 5.1 Ligand Effects on the Geometric and Electronic Structures

#### 5.1.1 Metal Core Geometry

Pykkö extensively reviewed the theoretical chemistry of gold compounds earlier in this millennium, which summarized the main conclusions garnered from a large body of appropriate calculations.^[^ ^318^ ^]^ According to Gilb et al., the lowest‐energy and most stable structures of several bare Au*_n_* ^+^ (*n* < 7) cations are usually planar. However, crystallographic data of phosphine‐stabilized Au clusters shows that the Au core prefers a spherical‐like structure, as discussed in Section 3.1, indicating that ligands play an important role in their geometry. Using computational methods, the effect of ligation on the relative stability of structural isomers of Au clusters has been investigated. One of the interesting studies is an analysis of charge transfer from Au–Au to Au–P bonds that leads to a stronger Au–ligand covalent bond and weakens the Au–Au bonds due to compressive strain.^[^ ^319^ ^]^ This means that the introduction of ligands not only causes steric effects but also electronic ones. For example, Burgos et al. concluded that the planar structures are distorted once ionized, as observed in Au_8_. Moreover, they found that the capacity of small Au nanoclusters to accept or donate electrons is greatly influenced by the number and spatial configuration of the phosphine ligands.^[^ ^320^ ^]^

To simplify matters, ligand effects on the metallic core are usually studied using truncated ligands to reduce computational expense. For example, use of the PH_3_ model instead of PPh_3_. This model ligand was found to be good enough to replicate the Au core structure.^[^ ^316b^ ^]^ However, it has been found that the slightly larger model PMe_3_ is needed for better relative energies and dipole moment calculations.^[^ ^321^ ^]^

#### 5.1.2 Electronic Stability

While Häkkinen and co‐workers provided the details on why Au clusters with magic numbers are stable,^[^ ^173a^ ^]^ there have been several other reports describing why phosphine‐stabilized Au clusters have electronic stability. Lugo et al. reported that charge redistribution between the gold core and the ligand shell can be controlled by the electron acceptor or donor character of the ligands and their spatial distribution around the metallic core.^[^ ^322^ ^]^ Although Au_70_S_20_(PPh_3_)_16_ does not satisfy the superatom complex theory of Häkkinen, a truncated model of the gold metalloid, Au_70_S_20_(PH_3_)_12_, approximated the ligand effect on the electronic stability of the molecule. Kenzler et al. found that the Au_58_S_20_ core is already stable but the PPh_3_ ligands stabilize the metalloid further by increasing the HOMO–LUMO gap.^[^ ^112^ ^]^ They reported a similar conclusion with an even bigger metalloid, Au_108_S_24_(PPh_3_)_16,_ using the truncated model.^[^ ^323^ ^]^ Moreover, Tian et al. supported the reported electronic stability of this metalloid by using the nucleus‐independent chemical shift analysis (NICS) proposed by Stanger,^[^ ^324^ ^]^ whereby they found that the inner Au_4_ cluster has its own strong aromaticity that helps provide the stability.^[^ ^325^ ^]^ The effects of icosahedral symmetry and ligands on how the clusters merge to form larger clusters via wrapping, bonding, or vertex sharing have also been reported recently.^[^ ^183^ ^]^

### 5.2 Ligand Bond Dissociation Energies

Goel et al. conducted a study regarding the preference of Au clusters for phosphine‐based ligands rather than amino‐stabilized ligands, concluding that the binding energies of phosphines are stronger than amines. Additionally, they also found that there is an increase in binding energy when the number of ligands decreases as a result of extra electron delocalization.^[^ ^326^ ^]^ This theoretical finding is important in the study of ligand bond removal of phosphine‐stabilized clusters.^[^ ^15^ ^]^ Furthermore, the presence of ligands such as PPh_3_ make contributions to the binding energies, not just by charge transfer but also due to the ligand–ligand interactions themselves.^[^ ^327^ ^]^

The removal of ligands is known to be an important method to enhance the reactivity of Au clusters in applications such as catalysis.^[^ ^274^ ^]^ Experiments showed that at least 150 °C is needed to thermally remove phosphine ligands.^[^ ^15^ ^]^ It had been previously reported that the binding energies increase as ligands are sequentially removed due to the electron delocalization.^[^ ^326^ ^]^ Mixed‐ligand clusters offer a different story—clusters stabilized by both PMePh_2_ and PPh_3_ have different ratios of binding energies, depending on their order of dissociation.^[^ ^288^ ^]^ However, the main conclusion is still the same; ligand–ligand interactions heavily affect the binding energies, ergo, dispersion corrections of fully ligated clusters are needed for simulations.^[^ ^288^, ^327^ ^]^ Using a plane wave PBE level of theory, the dissociation energy of PPh_3_ from AuCl was estimated to be 59.8 kcal mol^‐1^ and compared to an experimental value of 57.9 kcal mol^‐1^.^[^ ^158^ ^]^ The reported values are close to previously calculated dissociation energies using a local density functional with a linear combination of Gaussian‐type orbitals^[^ ^316b^ ^]^ and MP2/LANL2DZ^[^ ^317^ ^]^ levels of theory.

**Figure 15:**
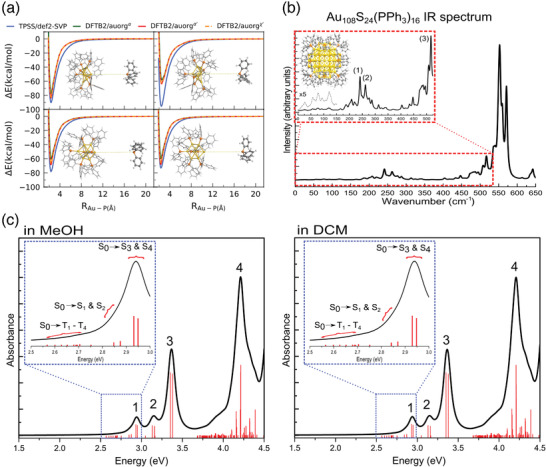
Quantum chemical simulations of the different properties of phosphine‐stabilized Au clusters where a) rigid Au–P bond dissociation energy curves of [Au_8_(PPh_3_)_8_]^2+^ were calculated using DFT and DFTB2/auorg parameters, b) optimized geometry (inset structure) of the Au_108_S_24_(PPh_3_)_16_ cluster and the predicted far‐IR spectra (right) calculated using DFTB2/auorg^*α*’^. Reproduced with permission.^[^ ^328^ ^]^ Copyright 2020, Royal Society of Chemistry. c) Calculated SO‐TDDFT UV−vis absorption spectra of the [Au_9_(PPh_3_)_8_]^3+^ cluster in both DCM and MeOH. Reproduced with permission.^[^ ^215^ ^]^ Copyright 2021, American Chemical Society.

A notable combined DFT and experimental study conducted by Kilmartin et al. showed an increase in binding energies after each ligand removal from the [Au_6_(Ph_2_P‐*o*‐tolyl)_6_]^2+^ cluster, wherein the binding energies increased from 150 up to 540 kJ mol^‐1^. Moreover, they found significant rearrangements of the Au cluster core after each successive removal of a ligand. Using in situ XAS, they showed partial removal of phosphine ligands at ≈90 °C, as well as a possible reorganization of the cluster core. Their findings also showed that naked Au particles are necessary to initiate catalytic oxidation of benzyl alcohol.^[^ ^274^ ^]^ Recently, tight‐binding parameters for the Au–P interactions were generated and tested.^[^ ^328^ ^]^ One of the applied benchmarks was the analysis of ligand bond dissociation of [Au_8_(PPh_3_)_8_]^2+^ compared with DFT (**Figure** **15**a). This work demonstrated that using these parameters in DFTB formalism, ligand removal energy pathways can be calculated with computational ease for any phosphine‐stabilized Au cluster that is commonly de‐ligated for catalytic applications.

### 5.3 Simulation of Far‐Infrared and Raman Spectroscopy

The use of infrared spectroscopy has applications beyond the determination and characterization of Au cluster structures—it also can be used to elucidate ligand–ligand interaction effects^[^ ^329^ ^]^ and adsorption binding to other molecules such as cis‐platin.^[^ ^330^ ^]^ Several studies have already reported on ligand‐stabilized Au clusters, and normal modes of vibration have been assigned to Au–Au core distortion and Au–P stretches.^[^ ^313^, ^329^, ^331^ ^]^ Notably, Alvino et al. provided the first far‐IR study of phosphine‐stabilized Au clusters using combined synchrotron experiments and IR simulations based on DFT. Their work established the Au metal core vibrations to be in the <200 cm^−1^ region, while the Au–P stretches have characteristic peaks at 450 and 500–600 cm^−1^ regions.^[^ ^313^ ^]^ An additional study by Bennett et al. showed that the addition of counterions in the simulation might be necessary for replicating some peaks in the far‐IR spectrum.^[^ ^331c^ ^]^

There are only a handful of Raman experiments done on Au clusters. Tlahuice‐Flores and co‐workers simulated both IR and Raman spectra of Au clusters, mostly in the fingerprint region.^[^ ^329^, ^330^, ^331^, ^332^ ^]^ In particular, they reported the possibility of charge effects on the simulated Raman spectrum of [Au_13_(dmpe)_5_Cl_2_]^z+^ due to the structural distortion and electronic structure differences upon reduction of the charges. This may be useful in drug delivery applications, such as cis‐platin chemotherapy, due to the enhanced IR/Raman signals after interaction with the Au cluster.^[^ ^330^, ^332^ ^]^ Another report on the simulation of Raman spectra, while not exactly a quantum chemical simulation, applies Horace Lamb theory to free homogeneous elastic spheres. The mass load model approximation is added to Lamb's system of equations to identify breathing‐like and quadrupolar‐like modes for small Au clusters.^[^ ^333^ ^]^ Their results were compared to DFT and previous experimental work.^[^ ^313^ ^]^ Only recently, Kato et al. reported the terahertz Raman spectrum of [Au_8_X_2_(dppp)_4_]^2+^ in the gold cluster fingerprint region from 50 to 150 cm^−1^. They assigned their observed bands to vibrational modes predicted by DFT.^[^ ^334^ ^]^ Following the development of the DFTB parameters for Au–P interactions, the simulations of ground‐state properties such as IR and Raman spectra of very large phosphine‐stabilized clusters can be made 1000× faster than before. Figure 15b shows the optimized geometry of Au_108_S_24_(PPh_3_)_16_ and the predicted far‐IR spectra calculated using the DFTB2/auorg^*α*’^ developed by Vuong et al.^[^ ^328^ ^]^

### 5.4 Calculations of Optical Properties

To simulate the optical properties of any material requires a detailed understanding of its electronic levels (i.e., the description of molecular orbitals). Simulations of molecular orbital topological plots via population analyses aid in the analysis of optical properties. One such example is the study by Tsukuda and co‐workers regarding the crown and butterfly isomers of [Au_9_(PPh_3_)_8_]^3+^.^[^ ^23^ ^]^ They reported that the oblate superatom centered crown geometry of [Au_8_Pd(PPh_3_)_8_]^2+^ has a forbidden HOMO → LUMO transition due to a superatomic P → P transition.^[^ ^55^ ^]^ This provides insight into the structural isomerism of [Au_9_(PPh_3_)_8_]^3+^ which has two distinct isomeric structures, with the crown core having a similar electronic structure to Au_8_Pd and the butterfly core with an allowed HOMO→LUMO transition due to P→D characteristics.

Simulated absorption spectra can be done using time‐dependent formalisms such as time‐dependent density functional theory (TD‐DFT), or its tight‐binding counterpart (TD‐DFTB). For much smaller systems, one can employ the use of multireference methods such as CASSCF and MRCI but these are usually not possible for Au clusters due to the large computational costs. Therefore, truncating the phosphine‐based ligands to simpler models such as PH_3_ or PMe_3_ is often employed for simulations of absorbance spectra. However, Ivanov et al. have reported that while these simpler models might be enough for understanding the geometric and electronic structures of Au clusters, especially the metal core, the full ligand PPh_3_ is necessary to fully model the electronic spectra of Au clusters.^[^ ^335^ ^]^

The asymptotically corrected semi‐empirical functional (CAM‐B3LYP) seems to be the immediate choice for most researchers,^[^ ^322^, ^336^ ^]^ with LB94 and GRAC being the desired functionals for Aikens and co‐workers.^[^ ^337^ ^]^ To reduce computational costs, lower rung functionals such as BP86 and TPSS are used for geometry optimization and frequency calculations. Subsequently, functionals that work well for the simulations of absorbance spectra would be employed.^[^ ^211^, ^336^ ^]^ Karimova and Aikens have simulated the absorption spectra of both the D_2h_ core isomer of [Au_9_(PPh_3_)_8_]^3+^ and [Au_8_(PPh_3_)_8_]^2+^.^[^ ^337^, ^338^ ^]^ While the experimental spectrum agrees with the theoretical result for [Au_8_(PPh_3_)_8_]^2+^, the experimental spectrum of [Au_9_(PPh_3_)_8_]^3+^ in solution represents the alternative C_4_ isomer.^[^ ^215^ ^]^ Yao and Tsubota simulated the spectra for the two isomers of the Au_9_ core using the truncated ligand PH_3_ for their study of the [Au_9_(TPPS)_8_]^5−^ cluster; however, due to the symmetry constraints in the simulations, the experimental spectrum was compared to the D_2h_ isomer as well,^[^ ^339^ ^]^ despite the fact that the experimental crystal structure clearly shows the C_4_ core isomer.^[^ ^340^ ^]^ With the help of the experimental and electronic structure findings of Tsukuda and co‐workers,^[^ ^23^, ^55^ ^]^ recent work by us showed that the experimental UV–visible spectra of [Au_9_(PPh_3_)_8_]^3+^ in both MeOH and DCM should be assigned to the C_4_ core isomer (Figure 15c).^[^ ^215^ ^]^ Moreover, a spin–orbit correction is required to the TD‐DFT simulated spectra to understand the cluster's absorption tail and the fast intersystem crossing observed in the transient absorption spectrum of this Au cluster. Fagan et al. compared the high‐resolution experimental spectra^[^ ^198^, ^341^ ^]^ of [Au_8_(PPh_3_)_7_]^2+^ and [Au_9_(PPh_3_)_8_]^3+^ measured in the gas phase with that simulated by TD‐DFT using the GRAC model. While there is a striking resemblance to the [Au_8_(PPh_3_)_7_]^2+^ cluster, no similar match is reported for the [Au_9_(PPh_3_)_8_]^3+^ cluster using the structures of the two isomers, which the authors suggest is caused by either deficiency in the theoretical model used or the presence of another isomer.^[^ ^337c^ ^]^

Charge density distribution studies will be helpful to identify future applications of these clusters such as predicting reactivity with other molecules.^[^ ^342^ ^]^ A correlation between the increase of electronic charge in the Au core and a red‐shift of the optical absorption band was observed by Lugo et al. for Au_13_, Au_25_, and Au_28_.^[^ ^322^ ^]^ Similarly, Muñoz‐Castro reported that using N‐heterocyclic carbenes (NHCs) as ligands can shift the absorption band to lower energies.^[^ ^343^ ^]^ It is known that differences in symmetry alter the optical response of phosphine‐stabilized Au_25_ cluster, and the addition of halogens as a ligand to the metal core also causes red‐shifting by increasing ligand‐to‐metal charge transfer (LMCT).^[^ ^344^ ^]^

The increasing popularity of TD‐DFT for modeling absorption spectra of Au clusters has been a strong driver in extending its use to explain other photoinduced phenomena, such as fluorescence. Goel et al. have previously proposed that amino‐based ligands could have better fluorescence activity than phosphine‐based ligands based on calculated oscillator strengths.^[^ ^336^ ^]^ A cutting‐edge simulation of photoluminescence was performed by Weerawardene et al. when they identified Jahn‐Teller distortion of the S_1_ state of [Au_13_(dppe)_5_Cl_2_]^3+^ as the cause of the long fluorescence lifetime, revealed by optimizing the geometry of the first singlet excited state.^[^ ^211^ ^]^ Furthermore, they calculated nonadiabatic relaxation dynamics using the simplified model of the cluster to simulate the decay time constants of the excited states in their population studies, coupled with transient absorption spectra. Zhou et al. also pointed out that intramolecular charge transfer (ICT) from the Au core to the ligands was necessary to explain the long photoluminescence activity of [Au_20_(PPhpy_2_)_10_Cl_4_]Cl_2_. Furthermore, they revealed that solvent dynamics play a role in the surface state trapping of this cluster, as studied by transition density mapping and charge density differences (CDD).^[^ ^214^ ^]^ Wu et al. assigned cause of the photoluminescence of ultrasmall Au*_n_* clusters (*n* = 6–8) to the fast intersystem crossing (ISC) to the triplet state due to strong spin orbit coupling (SOC) in their CASSCF results.^[^ ^206^ ^]^ Quite recently, Tsukuda and his group reported the room‐temperature phosphorescence of doped gold clusters, M@Au_12_ (M = Ru, Rh, and Ir), with Ru@Au_12_ exhibiting the highest quantum yield of 0.37. Using the DFT‐optimized structures and energies of the first triplet and singlet excited states, they proclaimed that the possible low energy difference is responsible for the fast intersystem crossing from S_1_ → T_1_, resulting in efficient phosphorescence.^[^ ^345^ ^]^ Lastly, Dominguez‐Castro and Frauenheim employed our Au–P DFTB parameters in the Ehrenfest molecular dynamics simulations of pyrene‐functionalized Au_70_S_20_(PR_3_)_11_PH_2_Pyr and Au_108_S_24_(PR_3_)_15_PPh_2_Pyr based on the real‐time time‐dependent density functional tight‐binding (RT‐TDDFTB) approach.^[^ ^346^ ^]^ They reported an irreversible charge transfer from the gold core to the pyrene derivate not seen in other ligands (R = H, CH_3_, C_2_H_5_, and C_6_H_5_), thus extending the list of phosphine‐ligated Au clusters that can be applied to photonics and nanodevices.

### 5.5 Simulation of Circular Dichroism and Chirality Measure

Circular dichroism (CD) can be simulated using the same principle as absorption spectra and previous reviews of the computation of optical rotation by TD‐DFT are available elsewhere.^[^ ^347^ ^]^ For Au clusters, the CD spectra of the D_2h_ core isomer of [Au_9_(PPh_3_)_8_]^3+^ and [Au_8_(PPh_3_)_8_]^2+^ have been simulated alongside their UV–visible absorption spectra.^[^ ^337^, ^338^ ^]^ Moreover, Karimova and Aikens also reported that certain clusters with BINAP ligands, such as Au_11_ and Au_8_, could have CD activity resulting from i) the core deformation due to the ligation, ii) the chiral nature of BINAP ligands, and (iii) the placements of Cl counterions.^[^ ^337b^ ^]^ Sato et al. have shown the possibility of two types of metal core in chiral Au_9_ with BINAP ligands that could lead to the cancellation of chiroptical response in the experiment.^[^ ^221b^ ^]^ They have also used the Hausdorff chirality measure (HCM) aside from the usual TD‐DFT formalism. In their recent study, they contend using HCM values that the chirality of Au_13_ is dominated by the DIOP ligand arrangement.^[^ ^348^ ^]^ On the other hand, Shichibu et al. reported very recently that the inner Au_5_ core of Au_13_ also contributes to chirality via transition state density and torsion analysis using DFT.^[^ ^219^ ^]^ Thus, while the chiral ligands definitely has a dominant effect in the CD activity, Au_13_ itself has an intrinsic metal core chirality.

## 6 Application in Catalysis

Although gold has a rich history of coordination and organometallic chemistry, it was originally considered to be catalytically inactive. Catalysis by small Au particles was first demonstrated by Bond et al. in the hydrogenation and isomerization of olefins in 1973.^[^ ^349^ ^]^ Bond and Sermon attributed the catalytic activity of gold to either surface defects or the small crystallite size, with the latter being the most plausible explanation.^[^ ^350^ ^]^ However, their works were forgotten for more than a decade until Haruta et al. later discovered the catalytic activity of small Au nanoparticles ≈5 nm in CO oxidation in 1987.^[^ ^351^ ^]^ These supported Au nanoparticles outperformed platinum group catalysts with higher stability and activity even at low temperature. Around the same time, Hutchings independently discovered Au chloride was catalytically active in hydrochlorination of acetylene.^[^ ^352^ ^]^ Since then, numerous studies have reported the catalytic activity of gold nanoparticles in a plethora of chemical reactions including selective oxidation, hydrogenation, cross coupling reactions, and CO_2_ conversion.^[^ ^353^ ^]^

There are numerous factors contributing to the high catalytic activity of Au catalysts: particle size, type of supports, metal–support interaction and charge transfer from/to the support, impurity doping, and dynamical fluxionality.^[^ ^354^ ^]^ Interestingly, the reduced Au–Au bond lengths and the quantum size effect in small Au clusters also contribute to high catalytic activity of Au clusters (cf. Au nanoparticles) providing favorable advantages.^[^ ^342^, ^355^ ^]^ A key advantage of employing Au clusters in heterogeneous catalysis is facile deposition onto metal oxides by manipulating electrostatic interaction between ligands and supports. Mousavi et al. demonstrated that Au_101_ clusters can be deposited on reduced graphene oxide (rGO) at high loading (5 wt%) without aggregation.^[^ ^258a^ ^]^ This deposition method is proven to produce a highly disperse and homogeneous distribution of Au clusters on the support.^[^ ^249^, ^356^ ^]^

Importantly, the weak Au–P bond advantageously allows ligand removal under mild conditions. It has been observed that the removal of phosphine ligands from the gold core can be achieved by calcination at 200 °C.^[^ ^15^ ^]^ Kilmartin et al. reported the removal of phosphine ligands from the Au_6_ core using organic peroxide (TBHP) at 95 °C.^[^ ^274^ ^]^ Moreover, Adnan et al. observed that the catalytic reaction mixture contained OPPh_3_ ligands (as confirmed by ^31^P NMR) indicating the removal of PPh_3_ ligands during the catalytic oxidation of benzyl alcohol at 80 °C.^[^ ^357^ ^]^ In these three cases, the growth of gold clusters into larger particles coincides with the removal of phosphine ligands.

Fluxionality of clusters is proposed to benefit catalysis by increasing the presence of more reactive metastable isomers which can potentially occur if the isomerization barrier is relatively low. Fluxionality has been demonstrated to dictate the catalytic activity of metal clusters on surfaces and the kinetics of catalysis.^[^ ^358^ ^]^ In most DFT studies of catalytic reaction mechanisms, the potential energy surface is not sampled thoroughly wherein only global minimum structures, a few intermediates, and transition state are calculated. However, clusters on surfaces have been reported to undergo restructuring,^[^ ^359^ ^]^ thus exhibiting dynamic fluxionality instead. For example, the CO oxidation of Au/CeO_2_ catalysts was found to arise from a transient Au^+^–CO species that lowers CeO_2_ reduction barriers.^[^ ^360^ ^]^ The ab initio molecular dynamics simulations show the dynamic formation of catalytic active sites that a straightforward DFT calculation would not be able to consider. Alexandrova and co‐workers reported that cluster catalysts should be considered as statistical ensembles in calculations to provide useful and complementary insights to experimental data.^[^ ^358a,b^ ^]^ The paradigm of cluster modeling is extended by including low energy metastable isomers present under catalytic conditions and the resulting ensemble of states could also dynamically evolve during the reaction. To calculate for the ensemble of distinct structural isomers, global optimization methodologies, and subsequent Monte Carlo simulations are required.^[^ ^361^ ^]^

### 6.1 Oxidation Reactions

Styrene oxidation is commonly used as a probe for testing catalytic activity of Au catalysts. Turner et al. demonstrated selective oxidation of styrene using Au catalysts derived from Au_55_ clusters (even though the synthesis of the clusters followed the protocol for Au_101_ clusters).^[^ ^257^ ^]^ The active species of the Au catalyst was ascribed to small size (<2 nm) Au clusters whereas larger (>2 nm) Au particles were found to quench the catalytic activity. The smaller cluster was suggested to have an altered electronic structure that enable Au clusters to chemisorb O_2_ molecules and dissociate them into oxygen adatoms for subsequent catalytic chemistry. Crucially, other works have also reported that such a quantum size effect is responsible for O_2_ activation in ultrasmall Au clusters.^[^ ^362^ ^]^

Considerable efforts utilizing computational calculations have also been undertaken to reveal the reaction mechanism and pathway, and the nature of the active sites of Au_55_(PPh_3_)_12_Cl_6_ clusters, in styrene oxidation. Pei et al. showed that the catalytic activity of Au_55_ clusters in styrene oxidation originates from the negative charge due to the electron back donation from PPh_3_ and the low coordination of the magic‐number Au_55_ core structure.^[^ ^342b^ ^]^ The authors attributed the selective formation of benzaldehyde to the spatial confinement of oxametallacycle intermediates containing C—O bonds by PPh_3_ ligands, with four mechanistic pathways leading to benzaldehyde as the final product. In contrast, Gao et al. showed that O_2_ reacts directly with styrene on the surface of Au_55_ instead of dissociating into O atoms.^[^ ^363^ ^]^ Using bi‐icosahedral [Au_25_(PPh_3_)_10_(SR)_5_Cl_2_]^2+^ supported on SiO_2_, Liu et al. proposed that the higher selectivity to form benzaldehyde (75%) over styrene oxide (16%) is associated with the relatively electropositive Au sites that result from charge transfer to ligands, as evidenced by the d‐band shift in UPS and Au L_3_‐edge and P K‐edge XANES results.^[^ ^275^ ^]^

The existence of isostructures in PPh_3_‐ligated undecagold gives rise to different chemical reactivity and stability, thus it is imperative to understand the impact of isostructure in catalysis. Wang et al. investigated this effect using Au_11_(PPh_3_)_7_Cl_3_ and [Au_11_(PPh_3_)_8_Cl_2_]Cl in styrene oxidation.^[^ ^303^ ^]^ For a wide range of Au loading (0.05–1.2 wt%) on SiO_2_ as‐prepared catalysts, Au_11_(PPh_3_)_7_Cl_3_ clusters showed a slightly higher catalytic activity than [Au_11_(PPh_3_)_8_Cl_2_]Cl. One possible explanation suggested by the authors is that the extra PPh_3_ in the latter cluster hinders the access of styrene to the catalytically active Au core, resulting in the observed lower catalytic activity. The Au_11_(PPh_3_)_7_Cl_3_/SiO_2_ catalyst was found to be more robust as it showed no decline in catalytic activity after six catalytic test cycles even though it has less steric hindrance.

Tsukuda and co‐workers demonstrated size‐dependent catalytic activity of undecagold clusters using alcohol oxidation as a model reaction.^[^ ^226^, ^227^ ^]^ The authors observed that the catalytic activity decreased with increasing Au particle size. A similar observation was also made by Kilmartin et al. using supported Au_6_(Ph_2_P‐o‐tolyl)_6_](NO_3_)_2_ clusters.^[^ ^274^ ^]^ However, Adnan et al. reported contrary results using a series of PPh_3_‐ligated Au clusters (Au_8_, Au_9,_ and Au_101_) supported on TiO_2_.^[^ ^357^, ^364^ ^]^ The catalytic activity only appeared for large Au nanoparticles (>2 nm) while small clusters were completely inactive for benzyl alcohol oxidation. The formation of large Au nanoparticles coincides with removal of PPh_3_ ligands by heat treatment or during catalysis which exposes the Au core to reactants/substrates. The inactivity of catalysts derived from Au_8_(PPh_3_)_8_(NO_3_)_2_ and Au_9_(PPh_3_)_8_(NO_3_)_3_ clusters was suggested to be due to NO_3_^−^ counter ions that inhibited the oxidation of benzyl alcohol. Surprisingly, the catalysts displayed improved activity after heat treatment under H_2_ atmosphere at 200 °C. The fate of NO_3_^−^ remains unclear because the ultra‐low loading of Au (0.17 wt%) impedes spectroscopic measurements but it was reported by Hirayama and Kamiya that NO_3_^−^ decomposed into N_2_ and N_2_O under a stream of H_2_, which may account for the increased catalytic activity.^[^ ^365^ ^]^ Nonetheless, no clear explanation was provided for the mechanistic action of NO_3_^−^ in the catalyzed reaction.

Donoeva et al. investigated the size effect in catalytic aerobic oxidation of cyclohexene by comparing Au_9_(PPh_3_)_8_(NO_3_)_3_ and Au_101_(PPh_3_)_21_Cl_5_ clusters supported on SiO_2_.^[^ ^366^ ^]^ By combining kinetic experiments with catalyst size evolution, as monitored using TEM and UV–visible DRS, the authors observed that there is an induction period where catalytic activity only appeared after large Au nanoparticles (>2 nm) formed during the catalytic reaction. Small, intact and phosphine‐free clusters (<2 nm) were found to be inactive. The induction period depends on how fast Au clusters grow into large Au particles; as such the induction period for Au_101_ is shorter than that of Au_9_ clusters. However, it should be noted that the origin of catalytic activity is not due to the plasmonic effect of Au nanoparticles since the catalytic reaction in the dark showed similar results.

A natural question to ask, given that partial ligand removal is a key step to substrate binding, is whether the uncoordinated sites are inherently useful for catalysis? Both theoretical and experimental works report promotional effects of uncoordinated/unsaturated Au sites. It has been demonstrated that Au_22_(L^8^)_6_ (L^8^ = 1,8‐bis(diphenylphosphino) supported on metal oxides (CeO_2_, TiO_2_, Al_2_O_3_) catalyzed CO oxidation without ligand removal owing to the uncoordinated Au sites providing active centers for CO adsorption and O_2_ activation.^[^ ^367^ ^]^ DFT calculations also reinforce that the uncoordinated Au sites are the active centers for strong O_2_ chemisorption, activation and dissociation by forming a peroxo state in Au_22_(L^8^)_6_O_4_.^[^ ^368^ ^]^ Wu et al. observed that the as‐synthesized Au_22_(L^8^)_6_ supported on rod‐shaped CeO_2_ (Au_22_(L^8^)_6_/CeO_2_‐r) readily achieved >80% CO conversion at 308 K.^[^ ^369^ ^]^ In situ EXAFS results showed that the Au–P distance and coordination number are similar in the as‐synthesized Au_22_(L^8^)_6_/CeO_2_‐r composite and the unsupported cluster, thus verifying that the ligands remain intact. It was found that CO oxidation proceeds via the Mars‐van Krevelen mechanism where the adsorbed CO on the Au sites is oxidized by the lattice oxygen of the support, in line with the previous finding using Au_144_(SR)_60_/CeO_2_.^[^ ^370^ ^]^

The nonscalable behavior of clusters makes it challenging to predict their catalytic performance. It has been found that small Au clusters exhibit even–odd alternating behavior in the electronic structures, optical properties, and oxygen binding energies.^[^ ^371^ ^]^ It is interesting and worth investigating if this oscillatory behavior is translated into catalysis. Donoeva and co‐workers demonstrated that Au_9_/CeO_2_ displays 2.2‐fold higher catalytic activity than Au_8_/CeO_2_ in CO oxidation.^[28d]^ Considering that one atom has a dramatic effect in catalysis, this finding is in line with the alternating even–odd electronic properties known for Au clusters.

For clusters, every atom counts and imparts significant effects on the fundamental properties including catalytic reactivity. Therefore, heteroatom substitution offers a viable strategy to tune the catalytic properties and study the structure–property relationships at atomic precision. It is expected that heterometallic clusters offer a superior catalytic performance due to the synergistic effects that improve the activity, kinetics, selectivity and stability compared to homogold clusters.^[^ ^39^, ^372^ ^]^ Recent work by Xu et al. reported that SiO_2_‐supported Au_8_Pd cluster achieved complete conversion and selectivity to benzaldehyde in benzyl alcohol oxidation whereas Au_9_, Au_25_ and Au_24_Pd were inactive.^[^ ^373^ ^]^ According to DFT calculations, O_2_ activation occurs at the central Pd atom while benzyl alcohol is activated at the edge Au sites, similar to a ligand exchange process, and that intracluster proton transfer catalyzes the oxidation. Such electron and hole mediation were not found for the other three clusters, which explains their inactivity.

### 6.2 Hydrogenation Reactions

Chemisorption and activation of H_2_ molecules on small Au particles offer a potential application of supported Au clusters in catalytic hydrogenation reactions.^[^ ^353^, ^374^ ^]^ Reactivity toward molecular H_2_ is often used as a gauge for hydrogenation reaction. Activation of H_2_ is often inferred from formation of HD in a H_2_−D_2_ exchange reaction to measure the catalytic activity of supported Au catalysts.^[^ ^375^ ^]^ Fujitani et al. reported that the rate of H_2_−D_2_ exchange increased as the size of the Au particle decreased, thus indicating a beneficial use of atomically precise Au clusters.^[^ ^376^ ^]^ DFT calculations by Hu et al. showed that unsaturated Au_22_(dppo)_6_ exhibits stronger hydrogen adsorption than Pt metal thus highlighting its potential as an alternative to conventional Pt catalysts in the hydrogen evolution reaction (HER).^[^ ^377^ ^]^

Platinum group metals (Pt, Pd, Ru, Rh) are extremely efficient catalysts for hydrogenation reactions.^[^ ^378^ ^]^ Numerous works have reported promising catalytic activity in the H_2_−D_2_ equilibration reaction at ambient pressure and temperature employing several gold–platinum clusters such as PtAu_6_, PtAu_8_, PtAu_9_ in solution, solid state, and on supports.^[^ ^66^, ^379^ ^]^ It was found that Pt−Au bonds give rise to more favorable active sites for H_2_ activation than the corresponding monometallic clusters. The catalytically active site has been ascribed to the Pt atom in the cluster core moiety.^[^ ^375^, ^380^ ^]^ A theoretical study by Xu et al. validated the role of Pt as the active center to bind H_2_ while Au atoms activate H_2_, illustrating the synergistic effects of both Au and Pt atoms in the PtAu_6_ cluster.^[^ ^381^ ^]^ Interestingly, hydrido mixed Au clusters demonstrated a better catalytic activity in H_2_−D_2_ equilibration.^[^ ^379^, ^382^ ^]^ These findings provide exciting opportunities to utilize hydrido clusters such as HPdAu_10_(PPh_3_)_8_Cl_2_, [H_3_Au_20_(PPh_3_)_12_](SbF_6_)_3_ and [Au_5_Re(H)_4_(PPh_3_)_7_](PF_6_)_2_ as hydrogenation catalysts.

Reduction of CO_2_ into high‐added value fuels and chemicals is a potentially viable approach to the synthesis of renewable energy sources and mitigation of global warming. Using three different clusters (Au_9_, Au_11_ and Au_36_) supported on metal oxides, Yang et al. recently reported that the core nuclearity strikingly influenced the selectivity of product formation in CO_2_ hydrogenation.^[^ ^353e^ ^]^ Au_9_, Au_11_ and Au_36_ primarily form methane, ethanol, and formic acid in high yield (>80%), respectively. The same group also demonstrated the superior catalytic activity and stability of montmorillonite supported PdAu_8_ over Au_9_, and the higher selectivity towards ethane cf. Au_9_ favoring formation of methane.^[^ ^383^ ^]^ DFT calculations revealed that Pd atom substitution inhibits structural reconstruction of the hexagonal motif during catalysis, which enhances the catalytic stability and activity. In the case of [Au_24_(PPh_3_)_10_(SR)_5_Cl_2_]^2+^, the presence of an internal vacancy (from the lack of a core Au atom) gives structural flexibility which prevents aggregation and consequently contributes to the high activity of CO_2_ hydrogenation to form dimethyl ether.^[^ ^384^ ^]^ These findings highlight that core nuclearity, composition, and structural geometry play critical roles in defining catalytic activity, selectivity, and stability.

**Figure 16:**
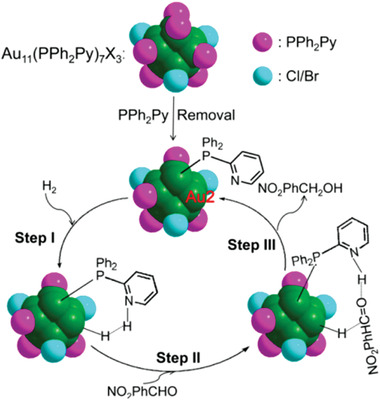
Mechanism for catalytic hydrogenation of nitrobenzyldehyde using supported Au_11_(PPh_2_Py)_7_Br_3_. Reproduced with permission.^[^ ^69^ ^]^ Copyright 2016 American Chemical Society.

Stabilizing ligands impart substantial influence on the catalytic performance of Au clusters owing to their different chemical reactivity. A fundamental question of how different ligands affect the catalytic performance has become the subject of recent studies. Haruta and co‐workers reported a markedly higher catalytic of Au_11_(PPh_2_Py)_7_Br_3_ for hydrogenation of nitrobenzaldehyde than the corresponding PPh_3_‐ligated homolog.^[^ ^69^ ^]^ Removal of one PPh_2_Py ligand was found to be imperative to expose the Au core (i.e., Au_11_(PPh_2_Py)_6_Br_3_) to substrates while the remaining, intact PPh_2_Py ligands were responsible for H−H bond activation, similar to the role of free amines. The proposed mechanism of hydrogenation catalyzed by Au_11_(PPh_2_Py)_7_Br_3_ is shown in **Figure** **16**. Wan et al. compared two different Au clusters, [Au_38_(PPh_3_)_4_(L)_20_)]^2+^ (L = alkylnyl or thiolate), in the semihydrogenation of alkynes and found a contribution of the alkylnyl ligand in activating molecular H_2,_ whereas complete ligand removal resulted in little activity of the cluster catalysts.^[^ ^385^ ^]^ Another study showed that intact ligands in the rod‐shaped Au_25_ cluster were found to preferentially catalyze hydrogenation of terminal alkynes with almost complete conversion and selectivity, while the ligand‐off cluster catalyzes internal alkynes to Z‐alkenes.^[^ ^386^ ^]^ DFT calculations suggested that terminal alkynes are activated and deprotonated at the waist sites of the cluster. To sum up, these findings conclude that partial ligand removal is a key step to allow substrate adsorption, while the remaining ligands define the catalytic activity and selectivity.

Large surface area and high porosity materials are often employed as supports to prevent sintering of metal clusters/nanoparticles. A special type, metal‐organic frameworks, are a promising support for preparation of robust and efficient catalysts.^[^ ^387^ ^]^ Additionally, defects are found to be beneficial for trapping and encapsulating clusters.^[^ ^200^ ^]^ The survival of unaggregated clusters on supports is critical for maintaining high catalytic activity as severe aggregation leads to decreased activity. Au_25_(PPh_3_)_10_(SR)_5_Cl_2_ encapsulated in microporous silica displays strong resistance against aggregation after calcination at 500 °C.^[^ ^388^ ^]^ The catalyst showed almost complete conversion and selectivity to ethylene at 300°C in acetylene hydrogenation with a low activation energy of 38.8 kJ mol^‐1^. Moreover, sintering and aggregation can be minimized or prevented by employing supports that have strong metal–support interactions (SMSI) such as CeO_2_, ZnO, TiO_2_, or hydroxyapatite (HAP).^[^ ^389^ ^]^ Recent work by Nesbitt et al. demonstrated that decent catalytic activity of H_2_‐activated HAP‐supported Au_19_, Au_20,_ and Au_22_ in the hydrogenation of nitroaromatics is due to the SMSI and possible charge transfer from the support to the clusters.^[^ ^390^ ^]^ The catalytic activity was found to increase linearly with the cluster size for Au*_n_*/HAP‐H_2_ (*n* = 19, 20, 22) catalysts, therefore featuring the effect of “one‐atom‐counts” in catalysis. However, the same clusters supported on Degussa P25, TiO_2_ anatase, and CeO_2_ display different trends. It is possible that other nontrivial factors such as cluster geometry and charge state are at play.

### 6.3 Photocatalysis

Recently, photocatalytic systems incorporating Au clusters have gathered attention owing to their ability to act as both a narrow bandgap photosensitizer and cocatalyst.^[^ ^391^ ^]^ The presence of HOMO–LUMO gaps in Au clusters renders them similar to narrow bandgap semiconductors which may have merit for efficient photocatalysis.^[^ ^392^ ^]^ Many photocatalytic reactions including water splitting, photodegradation of dyes and photoreduction of aromatic nitro compounds utilize Au clusters as the key component.^[^ ^393^ ^]^ However, research on photocatalysis utilizing Au clusters is still in its infancy.^[^ ^394^ ^]^ Fundamental questions including the effect of ligand, charge state and electron transfer, and core size nuclearity must be addressed to understand the photocatalytic mechanism and activity.

Singlet oxygen (^1^O_2_) is a highly reactive oxygen species that acts as an oxidant in catalytic reactions. A typical method to produce ^1^O_2_ is via energy transfer from an excited triplet state of a photosensitizer to the ground state of triplet oxygen (^3^O_2_) and electron exchange to yield ^1^O_2_. The [Au_13_(dppe)_5_Cl_2_]Cl_3_ cluster has been found to display a low energy gap (1.9 eV) and possess a high quantum yield (0.71) in ^1^O_2_ photogeneration, which is much higher than some thiolate‐protected counterparts.^[^ ^253^ ^]^ Similar results have been found for the crown isomer of [Au_9_(PPh_3_)_8_]^3+^ photogenerating ^1^O_2_ that leads to the degradation of 1,3‐diphenylisobenzofuran (DPBF).^[^ ^215^ ^]^ It has also been shown that generation of singlet (^1^O_2_) by photoexcited thiolate‐protected Au_25_ and Au_38_ clusters catalyzed photocatalytic oxidation of phenyl methyl sulfides and benzylamine under visible light irradiation.^[^ ^395^ ^]^

While coupling Au nanoparticles with semiconductors generates hot electrons and facilities charge transfer via plasmonic effects, Au clusters behave differently. The presence of a HOMO–LUMO energy gap enables visible light absorption by clusters and thus allows them to behave like excitonic materials. As a result, supported Au clusters on semiconductor photocatalysts like TiO_2_ have advantageous characteristics such as strong visible light absorption, efficient electron–hole separation and transfer, and generation of singlet oxygen (^1^O_2_).^[^ ^391a^ ^]^

**Figure 17:**
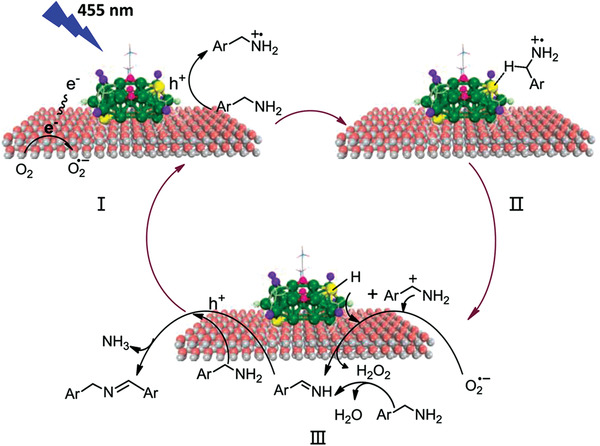
Mechanism of photooxidation of benzylamine using a Au_25_/P25 photocatalyst. Reproduced with permission.^[^ ^396^ ^]^ Copyright 2017 American Chemical Society.

Using a series of PPh_3_‐ligated Au clusters (Au_11_, Au_25_, Au_101_ core) supported on P25 (TiO_2_), Chen et al. investigated their performance in the photooxidation of amines to imines.^[^ ^396^ ^]^ The mechanism proceeds through generation of radicals (O_2_^●−^ and benzylamine radical cations) and formation of Au−H intermediates (**Figure** **17**). However, there was no explanation provided as to why Au_25_ was more active than Au_11_ and Au_101_. More recently, a Z‐scheme mechanism was proposed to contribute to the high catalytic activity and stability of Au_25_(PPh_3_)_10_(SRSi(OEt)_3_)_5_Cl_2_ supported on ultrathin 2D BiOCl, (Au_25_/2D‐BiOCl).^[^ ^397^ ^]^ The photoexcited electrons from the conduction band of 2D‐BiOCl recombine with the holes from Au_25_ to facilitate effective charge separation and transfer, and prevent self‐oxidation of the Au_25_ from the intrinsic holes.

Synergistic effects in heterometallic clusters bring multifunctional uses to photocatalysis such as narrow optical gap, improved photostability, multiple adsorption sites, and efficient charge transfer.^[^ ^394^ ^]^ For example, Ag doping in Au_11_(PPh_3_)_7_Cl_3_ results in two‐fold higher catalytic activity for the photooxidation of benzylamine, attributed to the reduced HOMO–LUMO gap of bimetallic Au_8_Ag_3_(PPh_3_)_7_Cl_3_ compared to Au_11_(PPh_3_)_7_Cl_3_ (1.67 and 2.06 eV, respectively).^[^ ^58^ ^]^ Other merits of doping are enhanced photothermodynamic and electrochemical stability, which paves the way towards robust photocatalysts. Recently, Qin et al. studied the effect of single Ag atom exchange in Au_13_Ag_12_(PPh_3_)_10_Cl_8_ for photocatalytic ethanol oxidation.^[^ ^254^ ^]^ It was found that the conversion of ethanol increased from 23% to 34% due to the difference in the electronic properties after the single‐atom exchange.

Ru clusters have been widely studied for their catalytic abilities.^[^ ^398^ ^]^ AuRu_3_/TiO_2_ photocatalysts derived from Ru_3_(µ‐AuPPh_3_)(µ‐Cl)(CO)_10_ showed superior photocatalytic activity in degradation of methylene blue under visible light compared to the homogold counterparts (Au_9_ cluster and Au nanoparticles).^[^ ^399^ ^]^ The improved performance is ascribed to the combined effects of LSPR‐active (due to aggregated AuRu_3_ clusters), which enabled visible light absorption, and unaggregated clusters. Recent work employing Au_4_Ru_2_(PPh_3_)_2_(SR)_8_ on TiO_2_ with rich oxygen vacancies demonstrated rapid photocatalytic reduction of N_2_ due to efficient exciton generation and charge transfer. The inclusion of Ru atoms plays a dual role in the photocatalysis: i) as an adsorption site for N_2_ and ii) to inject electrons into N_2_. Additionally, it has been shown that stabilizing ligands has little effect on the photocatalytic performance.^[^ ^400^ ^]^

Ligands can have a dominant influence in the photophysics and photochemistry of Au clusters. For example, the mixed‐ligand Au_23_(PPh_3_)_9_(SR)_4_ cluster assembled from tetrahedral building blocks generates more excitons than the Au_23_(SR’)_16_ homolog due to the structural variation induced by the metal–ligand bonding and thus leads to improved dye photodegradation.^[^ ^401^ ^]^ The secondary phosphine oxide (SPO) ligand also affects photocatalytic reactions; it has been demonstrated that a cooperative effect between Au and SPO leads to higher catalytic activity and selectivity toward C═O hydrogenation in the photocatalytic hydrogenation of benzaldehyde.^[^ ^402^ ^]^

## 7 Perspective and Future Prospects

Atomically precise phosphine‐ligated gold clusters offer opportunities for fundamental studies and have potential in many technological applications. Progress in cluster chemistry has enabled the synthetic preparation of a wide‐range of size‐selective gold clusters, including mixed‐metal and mixed–ligand composition, forming clusters with a high precision in the composition and number of atoms. The rich chemistry of gold–phosphine clusters offers promise as functional materials with a plethora of applications such as catalysis, sensing, and imaging. However, their practical realization is restricted by several significant challenges and further work is required to address these issues to materialize their full potential.

While crystallization and structural determination are less of a problem, the unsatisfactory yield for some gold–phosphine clusters such as Au_14_(PPh_3_)_8_(NO_3_)_4_ and [Au_6_(P(*p*‐tol)_3_)_6_](BPh_4_)_2_ pose a challenge for further studies. A lack of knowledge about their chemistry including formation mechanism, reactivity, and stability impedes the preparative work of high‐yield gold–phosphine clusters. A major focus should be directed to exploring novel synthetic strategies and routes that produce high yields and allows complete characterization. For example, Tsukuda and co‐workers successfully enhanced the yield of PdAu_8_(PPh_3_)^2+^ from 58% to 80% through kinetic control of the reduction and postsynthesis steps.^[^ ^55^ ^]^ With guidance from mass spectrometric studies, the initial species and intermediates responsible for the formation of the final clusters have been identified. It is possible to perform real‐time monitoring on other low‐yield clusters, identify the initial species and kinetically control intermediates to direct the synthesis of size‐specific clusters.

Compared to thiolate‐protected Au clusters, gold–phosphine clusters lack larger core size nuclearity. The metal‐to‐nonmetal transition has been found to occur between Au_246_ and Au_279_ in thiolate‐protected Au clusters. However, this is not necessarily the case for gold–phosphine clusters because the electronic structure is largely defined by the metal–ligand interaction. Thus, the influence of phosphine ligands is nontrivial. The structure of Au_101_(PPh_3_)_21_Cl_5_ cannot be established by X‐ray diffraction due to the size polydispersity and the lack of single crystals.^[^ ^113^ ^]^ Yet this cluster displays an LSPR band around 520 nm indicating metallic character. The largest phosphine‐ligated gold cluster that has been synthesized and structurally characterized so far is Au_108_(PPh_3_)_16_S_24_.^[^ ^114^ ^]^ Hence, there is a need to develop the synthesis of gold–phosphine clusters beyond Au_100_ to probe the transition from non‐metal to metal behavior.

The reactivity and stability of gold–phosphine clusters are of particular interest and have not yet been fully explored. Reactivity has been studied in ligand exchange process and fragmentation in mass spectrometry. Two interesting outcomes from ligand exchange studies are formation of new clusters and mixed‐ligand composition. Understanding the reactivity of clusters is vital for preparation of new clusters by reacting existing clusters with appropriate reagents/ligands. For example, Murray and co‐workers reported that the reaction between Au_55_(PPh_3_)_12_Cl_6_ with hexanethiol produced Au_75_(SC_6_H_13_)_40_.^[^ ^8c^ ^]^ Jin et al. recently prepared three new clusters (Au_13_, Au_18_, Au_20_) by thiol‐induced synthesis that are stabilized with diphenylphosphinomethane (dppm). Ren et al. demonstrated that cluster transformation among diphenylphosphinopropane (dppp)‐ligated Au_6_, Au_8_ and Au_11_ was feasible by manipulating reductant, oxidant, and heating.^[^ ^73^ ^]^ These works highlight that synthesis of novel clusters can be achieved by understanding and exploiting the reactivity of gold–phosphine clusters. A future direction dedicated to study the reactivity and stability of these clusters will broaden the inventory of atomically precise gold clusters.

The use of phosphines as stabilizing ligands is not limited to gold. Numerous phosphine‐ligated metal clusters including Pt, Pd, Ir, Re, Ag, Cu and Ni have been synthesized and reported. It is interesting to note that early works on these clusters often involved co‐ligands such as carbonyl (CO), hydride, halides, C_2_H_4,_ and cyclooctadiene (COD). The inclusion of hydride or CO ligands in some clusters results from their use as a reducing agent. It also illustrates the high reactivity of these metal clusters toward small molecules, which might be beneficial in catalysis. Pignolet et al. suggested that hydride‐adduct clusters could serve as precursors for formation of larger clusters.^[^ ^403^ ^]^ It might be possible to extrapolate the findings from the synthesis of gold‐phosphine clusters into other clusters. For example, studies on bimetallic phosphine‐ligated gold–silver clusters had been done extensively by Teo and co‐workers over the past 35 years.^[^ ^87^, ^404^ ^]^ However, the progress in synthesis and properties of homonuclear silver clusters is still in its infancy.^[^ ^405^ ^]^ It is thus expected that the knowledge of gold–phosphine clusters could contribute to the expansion of research using other metal clusters.

Due to high computational cost and the time‐consuming process of both wavefunction and density functional theories, several assumptions must be made to simplify calculations of gold–phosphine clusters. In many cases, the PPh_3_ ligand is approximated by PH_3_ or PMe_3_ and assumes that changing the functional groups has little to no effect on the cluster stability, cluster–ligand and ligand–ligand interactions. However, this assumption is not always true and therefore more accurate and faster computational models are required that negates the need to truncate the ligands. The recent development of the density functional tight‐binding parameters for the Au–P interaction has paved the way for faster calculation of ground‐state properties such as geometric and electronic structures and energetics, alongside the simulation of infrared spectra for phosphine‐stabilized Au clusters.^[^ ^328^ ^]^ The accelerated calculation of the optical properties of these nanoclusters using time‐dependent DFTB could soon be the standard as the simulated absorption spectra of the large Au_70_S_20_(PPh_3_)_12_ and Au_108_S_24_(PPh_3_)_16_ clusters have recently been reported.^[^ ^346^ ^]^ Likewise, the development of tight‐binding parameters involving the interaction of Au and P with different substrate surfaces like titania is necessary to further improve material design and reaction tunability of cluster‐based heterogeneous catalysts. Furthermore, extending the use of DFTB in the systematic sampling of potential energy surfaces could provide faster and more efficient ways to calculate the metastable states necessary to consider the dynamics within heterogeneous catalysis.

Further research in catalysis using supported gold clusters offer the means to reduce the cost of catalyst fabrication by minimizing the amount of metal used whilst maintaining high catalytic performance. More importantly, the catalytic performance is highly dependent on the cluster size, electronic structure, fluxionality, and interaction between cluster and a support. A challenging problem is aggregation of clusters on a support after ligand removal due to reduced surface energy and chemical potential. Aggregation complicates the correlation between structure and activity relationships because of structural and size changes during a reaction. Strategies to prevent aggregation such as the use of large surface areas, highly porous supports, creation of defects, ultralow gold loading and introduction of functionalized sites have been reported.^[^ ^250^, ^258^, ^406^ ^]^ Alotabi et al. recently demonstrated that deposition of a thin (1.1 nm) layer of Cr_2_O_3_ onto Au_9_/TiO_2_ inhibits agglomeration of gold clusters after ligand removal by heating.^[^^]^ More recently, Kollmannsberger et al. encapsulated Au_8_ clusters inside ZIF‐8 via a bottle‐around‐ship approach.^[^ ^250^ ^]^ This strategy allowed deposition of high gold loading (up to 8 wt%) and calcination under vacuum up to 350 °C without agglomeration. Quite interestingly, the emergence of single‐atom catalysis paves the way towards rational catalyst design.^[^ ^407^ ^]^ Hence, there lies opportunities to borrow ideas and principles from supported single atom to cluster‐based catalysts.

## Conflict of Interest

The authors declare no conflict of interest.
